# Schizophrenia‐like topological changes in the structural connectome of individuals with subclinical psychotic experiences

**DOI:** 10.1002/hbm.22796

**Published:** 2015-04-02

**Authors:** Mark Drakesmith, Karen Caeyenberghs, Anirban Dutt, Stanley Zammit, C. John Evans, Abraham Reichenberg, Glyn Lewis, Anthony S. David, Derek K. Jones

**Affiliations:** ^1^ Cardiff University Brain Research Imaging Centre (CUBRIC), School of Psychology, Cardiff University Cardiff United Kingdom; ^2^ Neuroscience and Mental Health Research Institute (NMHRI), School of Medicine, Cardiff University Cardiff United Kingdom; ^3^ Department of Physical Therapy and Motor Rehabilitation Faculty of Medicine and Health Sciences University of Ghent Gent Belgium; ^4^ School of Psychology Faculty of Health Sciences Australian Catholic University Melbourne Australia; ^5^ Institute of Psychiatry Psychology and Neuroscience, King's College London DeCrespigny Park London United Kingdom; ^6^ Centre for Academic Mental Health School of Social and Community Medicine University of Bristol Bristol United Kingdom; ^7^ Department of Psychiatry Icahn School of Medicine Mount Sinai Hospital New York New York USA; ^8^ Division of Psychiatry Faculty of Brain Sciences University College London London United Kingdom

**Keywords:** psychosis, schizophrenia, connectomics, tractography, graph theory, structural connectivity, diffusion MRI, psychotic experiences, network efficiency, ALSPAC, birth cohort, psychosis risk, neuropsychiatry, epidemiology

## Abstract

Schizophrenia is often regarded as a “dysconnectivity” disorder and recent work using graph theory has been used to better characterize dysconnectivity of the structural connectome in schizophrenia. However, there are still little data on the topology of connectomes in less severe forms of the condition. Such analysis will identify topological markers of less severe disease states and provide potential predictors of further disease development. Individuals with psychotic experiences (PEs) were identified from a population‐based cohort without relying on participants presenting to clinical services. Such individuals have an increased risk of developing clinically significant psychosis. 123 individuals with PEs and 125 controls were scanned with diffusion‐weighted MRI. Whole‐brain structural connectomes were derived and a range of global and local GT‐metrics were computed. Global efficiency and density were significantly reduced in individuals with PEs. Local efficiency was reduced in a number of regions, including critical network hubs. Further analysis of functional subnetworks showed differential impairment of the default mode network. An additional analysis of pair‐wise connections showed no evidence of differences in individuals with PEs. These results are consistent with previous findings in schizophrenia. Reduced efficiency in critical core hubs suggests the brains of individuals with PEs may be particularly predisposed to dysfunction. The absence of any detectable effects in pair‐wise connections illustrates that, at less severe stages of psychosis, white‐matter alterations are subtle and only manifest when examining network topology. This study indicates that topology could be a sensitive biomarker for early stages of psychotic illness. *Hum Brain Mapp 36:2629–2643, 2015*.© **2015 TheAuthors Human Brain Mapping Published by Wiley Periodicals, Inc**.

## INTRODUCTION

Schizophrenia has long been conceptualized as a “dysconnectivity syndrome” [Friston, 1998] in that the interconnections between modular systems in the brain are awry [David, 1994]. Several neuroimaging studies using magnetic resonance imaging (MRI) have uncovered dysconnectivity in several neural pathways using both structural and functional neuroimaging [Pettersson‐Yeo et al., 2011; Shenton et al., 2001; Wheeler and Voineskos, 2014] and have suggested that they may arise early in the course of, or even predate the disorder [Carletti et al., 2012]. The heterogeneity of structures that are implicated in schizophrenia suggests that the associated abnormalities manifest globally, rather than being focal to one particular region or pathway.

Richer insights into dysconnectivity in schizophrenia have recently been achieved using graph theory (GT), a powerful mathematical framework that quantifies topological features of networks [Bullmore and Sporns, 2009; Guye et al., 2010; Rubinov and Sporns, 2010; Stam and Reijneveld, 2007]. GT can be used to measure aspects of network integration, segregation, centrality, and other architectural features and has been applied in numerous studies examining structural and functional networks in clinical groups [Griffa et al., 2013; Petrella, 2011]. The approach provides more insights into structural changes that can take place in less severe forms of the disease, which may be too subtle to be detected at the local level.

Previous studies have reported several structural connectome changes in schizophrenia patients by applying GT to tractography data from which clear patterns have emerged [Griffa et al., in press; Van den Heuvel et al., 2010, 2013; Wang et al., 2012; Zalesky et al., 2011; Zhang et al., 2015] (summarized in Table 1—See also [Fornito and Bullmore, 2014; Rubinov and Bullmore, 2013]). Although there is variability in the findings, there are also some clear trends. In particular, there are reductions in measures of network integration, (specifically, reductions in density, strength, efficiency, and increases in path length—see methods for descriptions of these measures). These findings suggest that the structural layout of the brains of schizophrenia patients predispose them to the functional disturbances associated with schizophrenia. Other measurements such as mean clustering coefficient and smallworldness were generally found to be conserved.

**Table 1:** Summary of previous GT studies on structural connectomes in schizophrenia

Study	Sample	Acquisition parameters			Analysis				Results	
		Field strength (T)	# gradient directions	*b*‐value (mms^−2^)	Tractography method	network parcellation	Edge weights	Edge thresholding	Network‐level	Node‐level
Van den Heuvel et al. [2010]	40 Sz 40 HC	1.5	30	1,000	DTI (FACT)	AAL (108 WB)	FA MTR	*n.a*.	= PL, Clust. SW	↑ PL, ↓BC (frontal, cingulate and temporal regions)
										= Clust.
Zalesky et al. [2011]	74 Sz	1.5	64	1,000	DTI (FACT)	AAL (82 CC)	B	0‐8 SLs (independently tested)	↓ GE. Den.	None tested
	32 HC								= PL, Clust, SW	
Wang et al. [2012]	79 Sz	1.5	13	900	DTI (FACT)	AAL (90 WB exc. Cerebel.)	SL	1‐10 SL (such that full connectivity is maintained)	↓ GE	↓E. (frontal, paralimbic/limbic and subcortical regions)
	79 HC									
Zhang et al. [2014]	30 Sz (1^st^EpDN)	1.5	15	1,000	DTI (FACT)	AAL (90 WB exc. Cerebel.)	SL	1‐6 SL (independently tested)	↓ Str., GE, Den.	↓ E (cingulate and parietal cortices, basal ganglia, and limbic‐visual system systems).
									↑ PL	
	35 HC								= SW	
Van den Heuvel et al. [2013]	48 Sz	1.5	30	1,000	DTI (FACT)	DK (68 CC, 14 SC & RCN)	SL	*n.a*.	↓ Den.,(in RCN)	None tested
	45 HC						Den.		= GE, SW	
Ottet et al. [2013]	46 22q11DS 48 HC	3	30	1,000	*unspecifed*	AAL‐82 (CC & SCC)	B	*unspecifed*	↓ GE Den ↑ PL = SW	↓ Deg. (prefrontal/inferior frontal, parietal, Hippocampus, thalamus. and caudate)
										E ∝ AH (left frontal regions)

Collin et al. [2014]	40 S,	1.5	32	1,000	DTI (FACT)	DK (38 CC & RCN).	SL	5 SL	↓ Den. (in RCN)	↓Str, E, Clust (frontal, anterior cingulate, orbitofrontal and inferior temporal)
	54 H1^st^DR								↓ Str. GE, Clust.	
	51 HC									
Griffa et al. [in press]	16 Sz	3	128	Up to 8,000	DSI (FACT)	DK (CC & SCC)	nSD	none	↓ GE, Trans.,	↓ E Trans. (frontal, temporal, parietal and cingulate regions. Several subcortical structures).
	15 HC								= Clust. CC	

Abbreviations**: Sz**: Schizophrenia patients; **HC**: Healthy controls; **1** ^st^ **EpDN**: First episode drug‐naïve schizophrenia patients; **H1** ^st^ **DR**: First degree relatives of schizophrenia patients; **22q11DS**: 22q11 deletion syndrome patients; **DTI**: Diffusion tensor imaging [Basser et al., 1994]; **DSI**: Diffusion spectrum imaging [Wedeen et al., 2005]; **FACT**: Fiber Assignment by Continuous Tracking [Mori et al., 1999]; **AAL** Automated atlas labeling [Tzourio‐Mazoyer et al., 2002]; **DK**: Desikan–Killiany atlas [Desikan et al., 2006] **CC**: Cortica connections; **WB**: Whole‐brain; **SCC**: Subcortical connections; **RCN**: Rich‐club network **B**: Binary weights; **SL**: Streamlines; **nSD**: Normalized streamline density; **FA**: Fractional anisotropy; **MTR**: Magnetization transfer ratio [Wolff and Balaban, 1989]; **↑**: Significant increase; **↓**: significant decrease; **=**: no significant effects; ∝: Significantly correlates with; **Den**.: Density;: Significantly correlates with; **GE**: Global efficiency; **PL**: Path length; **Clust**.: Clustering coefficient **SW**: Smallworldness; **Str**.: Strength; **Deg**.: Node degree; **E**: Efficiency; **BC**: Betweeness centrality. **Trans**.: Transivity; **CC**: Closeness centrality; **AH**: Auditory hallucinations.

The literature also reveals some trends in network topology at the local level. [Van den Heuvel et al., 2010] showed that path length was higher in frontal and cingulate cortices, where Wang et al., [2012] and Zhang et al., [2015] also found reduced efficiency. A recent study [Van den Heuvel et al., 2013] shows that there is specific dysfunction in the “rich club” network in individuals with schizophrenia—a set of nodes that have a high tendency to be densely connected with each other [Van den Heuvel and Sporns, 2011]. This subnetwork comprises the superior parietal cortex, cuneus, frontal cortices, hippocampus, thalamus, and putamen. This also overlaps with the well‐defined default mode network (DMN) [Raichle et al., 2001] and the structural core identified by Hagmann et al., [2008].

The findings summarized in Table 1 were mostly derived from studies of patients with established illness who had been treated for many years with antipsychotic medication. It is, therefore, unclear whether the findings represent a core process in the development of the condition or are a downstream consequence of confounding factors. Zhang et al., [2015] identified network deficits in drug‐naïve first‐episode schizophrenia patients, making an important distinction between early and more chronic stages of the disease. Another study looked at network topology in individuals with 22q11 deletion syndrome [Ottet et al., 2013], which confers significantly increased risk of psychotic symptoms and found similar reduction in network integration, while other graph indices were preserved. Finally, Collin et al., [2014] have recently reported reduced rich club connectivity in first degree relatives (siblings) of patients with schizophrenia, mirroring the findings of Van den Heuvel et al., [2013].

Although these findings demonstrate reduced network integration in the connections in different manifestations of the disease, no study has yet performed the same analysis on an epidemiologically derived sample of individuals with psychotic experiences (PEs), who have not received a clinical diagnosis or sought any clinical intervention. Identifying topological changes in such a group would be a powerful means of identifying (a) neurological correlates of psychosis unconfounded by treatment. (b) biomarkers of disease risk and progression. Several studies have shown that PEs confer increased risk of developing clinically significant psychosis and are highly correlated with other risk factors. [Kelleher and Cannon, 2011; Poulton et al., 2000; Welham et al., 2009].

In this study, we investigated the network topology of structural connectomes in individuals with PEs from a large UK geographical birth cohort assessed systematically and prospectively rather than in help‐seeking individuals with psychotic disorders. Our aim was to test the hypothesis that such individuals had similar topological deficits to those described in the research literature, investigating patients presenting with psychotic disorder. We computed GT measurements of both global and local topology and also investigated a subset of functional networks to identifying particular functional modes that may be vulnerable to dysfunction.

## MATERIALS AND METHODS

### Subjects

We employed a population‐based sampling approach. Such an approach minimizes biases that can occur in recruiting subjects from clinical environments. Source of such bias include the reliance on self‐reporting, referral, and selection biases.

Subjects were recruited from the Avon Longitudinal Study of Parents and Children (ALSPAC) [Golding et al., 2001] cohort. The original sample consisted of pregnant women whose expected dates of birth were between April 1991 and December 1992. Thirteen thousand nine hundred and seventy one of the children survived until their first birthday, and detailed information has been collected periodically for health research. Four thousand three hundred and twenty subjects from the cohort were assessed for PEs using the Psychotic‐like symptoms semi‐structured interview [Horwood et al., 2008; Zammit et al., 2008], conducted at 17/18 years of age by trained psychologists. The definite or suspected presence of PEs was judged according to clinical criteria of the Schedule for Clinical Assessment in Neuropsychiatry [Wing et al., 1990] and excluded any such experiences occurring due to waking, falling asleep, fever, or drug consumption. Four hundred and thirty three subjects (about 10% of those tested) were found to have one or definite or suspected PE, all of whom were invited to undergo scanning, of whom 126 (29.1% of those invited) were scanned. Three thousand eight hundred and eighty seven subjects showed no signs of PEs, of whom 126 (3.24% of those eligible) were invited to undergo scanning. The control group were selected at random until the numbers in each groups were balanced. At the time of scanning, all subjects were around 20 years old. Further demographics for the sample are provided in Table 2.

**Table 2:** Demographic data for the sample and statistic tests to identify significant group effects in demographic variables

	With PEs	Without PEs	Statistics
**n**	123	125	
**Age (years)**	20.05 ± 0.002	20.10 ± 0.002	*F*_(1)_=0.479, *P* = 0.49
**Gender**			*χ* ^2^_(1)_ = 2.275, *P* = 0.19
Male	37	49	
Female	86	76	
**Handedness**			*χ* ^2^_(2)_ = 0.234, *P* = 0.89
Right	92	92	
Left	9	9	
No dominant hand	22	25	

Informed consent was obtained prior to scanning. Ethical approval was granted by the Cardiff University School of Psychology Ethics Committee and the ALSPAC Ethics and Law Committee. Of the subjects initially scanned, three PE subjects and one control were unable to complete the full MRI acquisition, reducing the sample sizes of the two groups to *n* = 123 and *n* = 125, respectively.

### MRI Acquisition

MRI data were acquired on a 3T General Electric HDx MRI system (GE Medical Systems, Milwaukee, WI) using an eight‐channel receive‐only head RF coil. A cardiac‐gated diffusion‐weighted spin‐echo echo‐planar imaging sequence was used to acquire high angular resolution diffusion weighted images (HARDI) [Jones et al., 1999]. Sixty gradient orientations and six unweighted (*b* = 0 s/mm^2^) images were acquired with the following parameters: TE = 87 ms, *b* = 1,200 s/mm^2^, 60 slices, slice thickness = 2.4 mm, FoV = 230 × 230 mm, Acquisition matrix = 96 × 96, resulting in data acquired with a a 2.4 × 2.4 × 2.4 mm isotropic resolution. Following zero‐filling to a 128 × 128, in‐plane matrix for the fast Fourier transform. The final image resolution was, therefore, 1.8 × 1.8 × 2.4 mm.

In addition, T1‐weighted structural images were acquired with a 3D fast spoiled gradient echo sequence (TR = 7.8 ms, TE = 3.0 ms, voxel size = 1 mm³ isomorphic).

### MRI Preprocessing

HARDI data were preprocessed in ExploreDTI v4.8.3 [Leemans et al., 2009]. Data were corrected for motion, eddy currents, and field inhomogeneities prior to tractography. Motion artefacts and eddy current distortions were corrected with B‐matrix rotation using the approach of Leemans and Jones [2009]. A comparison of subject motion between the two groups is shown in Supporting Information.

Field inhomogeneities were corrected using the approach of Wu et al. [2008]. Each diffusion‐weighted image (DWI) was nonlinearly warped to the T1‐weigted image using the fractional anisotropy (FA) map from the DWIs as a reference. Warps were computed using Elastix [Klein et al., 2010] using normalized mutual information cost function and constraining deformations to the phase‐encoding direction. The corrected DWIs are, therefore, in the same (undistorted) space as the T1‐weighted structural images.

### Tractography

Whole‐brain tractography was performed using the damped Richardson–Lucy algorithm [Dell'acqua et al., 2010], This is a modified spherical deconvolution method which is more robust to spurious peaks in the fiber orientation distribution (FOD) than standard spherical deconvolution methods [Tournier et al., 2007]. The tractography algorithm used is that of Basser et al. [2000] which uses a uniform step size. Seed points were arranged in a 3 × 3 × 3 mm grid in white matter, step size = 1 mm, angle threshold = 45°, length threshold = 20–500 mm, FOD threshold = 0.05, *β* = 1.77, *λ* = 0.0019, *η* = 0.04, number of iterations = 200 (See Dell'acqua et al., [2010] for full details of these parameters).

Additional anatomical constraints were introduced to ensure minimal contamination from spurious streamline trajectories through gray matter. A segmentation of the T1 weighted images was performed using FSL‐FAST and was used to apply a clipping mask to the streamlines, such that streamlines were forced to terminate when they entered gray matter. There was no explicit masking of cerebro‐spinal fluid (CSF), however, the termination criteria used for the dRL algorithm, which is based on the amplitude of the FOD peak, ensures that no streamlines enter isotropic regions such as CSF.

### Connectivity Matrices

**Figure 1:**
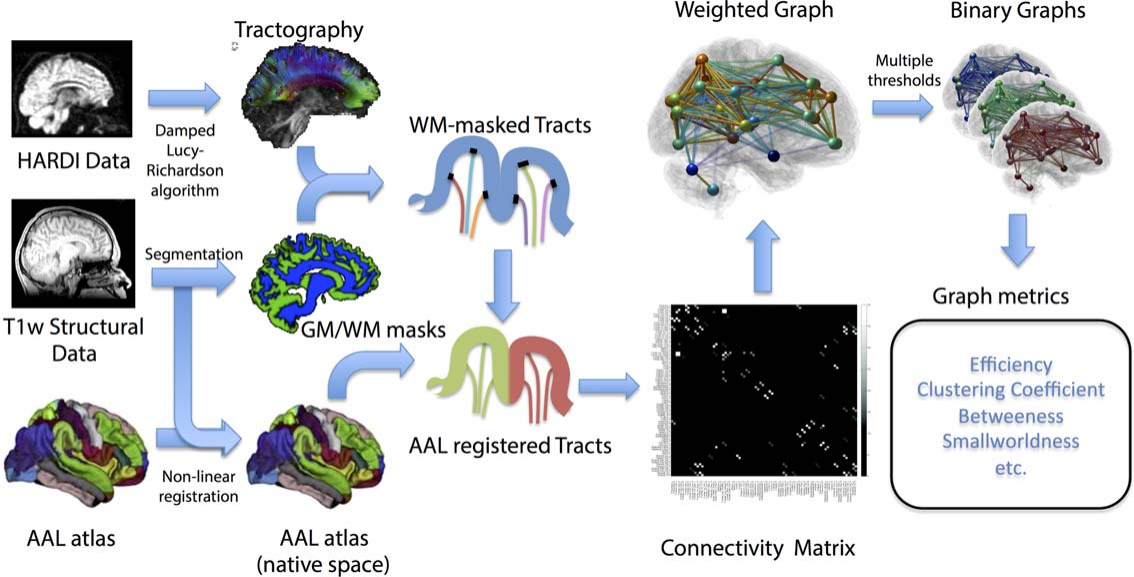
Flowchart of derivation of weighted graphs from tractography data. [Color figure can be viewed in the online issue, which is available at http://wileyonlinelibrary.com.]

Figure 1 shows a flowchart for the process of obtaining connectivity matrices. The automated atlas labeling (AAL) atlas [Tzourio‐Mazoyer et al., 2002] was registered to the HARDI data using a nonlinear transformation [Klein et al., 2010]. The streamline termination points were coregistered to each AAL region. The numbers of streamlines connecting each pair of AAL regions were aggregated into a 116 × 116 connectivity matrix. This matrix was then binarized at a range of thresholds based on streamline count, in the range of 0–20 streamlines. This was deemed the upper limit for which edges are unlikely to be due to false positives.

**Figure 2:**
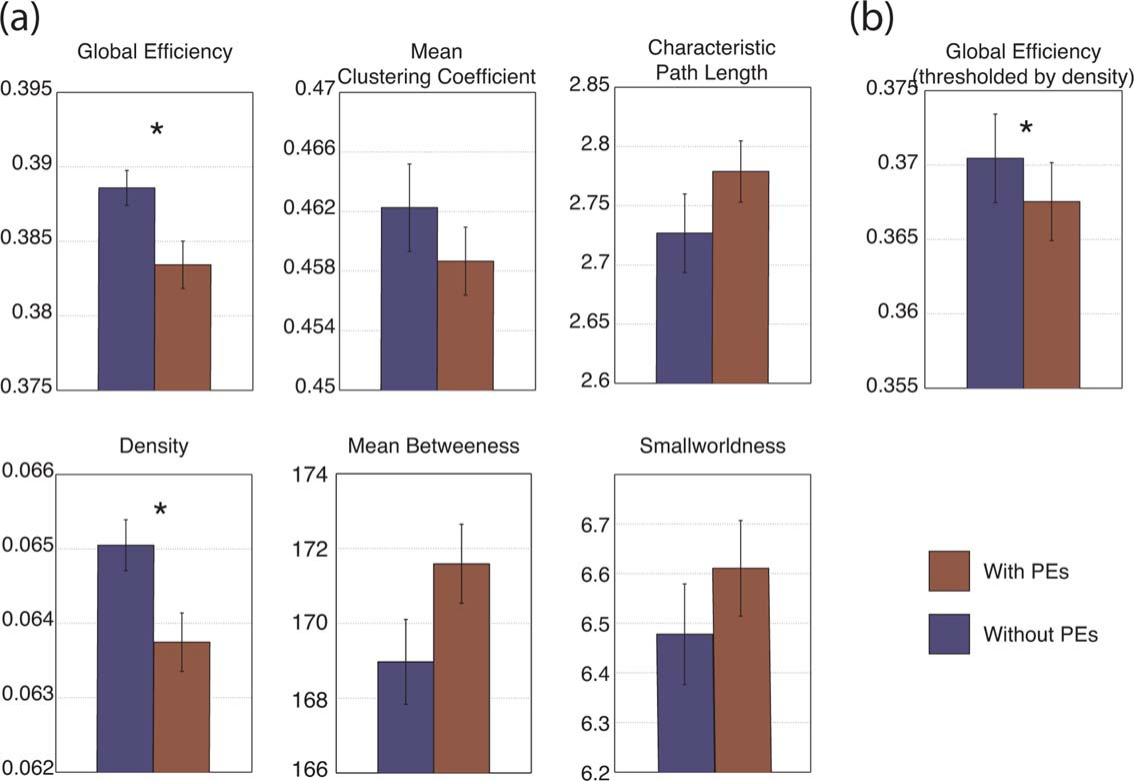
a: All results for network‐level analysis obtained using standard streameline thresholds. *P_corr_<0.05. **b**: Results for global efficiency obtained using density thresholds. (Results across all thresholds tested are shown in Supporting Information). [Color figure can be viewed in the online issue, which is available at http://wileyonlinelibrary.com.]

### GT Metrics

A range of commonly used graph theoretical measurements were used to characterize network topology at the global and local level. All GT metrics were computed using the Brain Connectivity Toolbox [Rubinov and Sporns, 2010]. The following network level GT metrics were computed:

#### Density

Density is a basic measure of network integration. It is the total number of edges in the network as a proportion of the total number of possible connections. Density is bounded by [0,1]. To node‐level equivalent is node degree, which is the number of edges connected to a node. Node degree is bounded by [0,*n*], where *n* is the number of nodes.

#### Global efficiency and characteristic path length

Global efficiency and characteristic path length [Latora and Marchiori, 2001] are measures of network integration. Global efficiency is formally the inverse of the characteristic path length, which is the mean minimum (geodesic) distance required to travel from one node to another. It represents how efficiently information can be transmitted across the network. Local efficiency and minimum distance are the equivalent node‐level metrics. Local efficiency of a node is the inverse of its minimum distance, which is the minimum (geodesic) distance required to travel from one node to another, while traversing the node of interest. Minimum distance and characteristic path length are bounded by [1, *m*] where *m* is the number of edges (assuming the network is fully connected). Efficiency measures are bounded by [1/*m*, 1].

Given that global efficiency is heavily dependent on density [Ginestet et al., 2011; Van Wijk et al., 2010], we also computed global efficiency with variable thresholds, such that density is kept constant across subjects. Densities of 0.05 to 0.1, in increments of 0.005 were tested, which is approximately the range of densities observed for this sample. This additional analysis will enable differentiation of effects on efficiency due to the quantitative effects of density and those that are due to more qualitative factors, such as network reorganization.

#### Mean clustering coefficient

Mean clustering coefficient [Onnela et al., 2005; Watts and Strogatz, 1998] is a measure of functional segregation. It is the tendency of the network to organize into functionally distinct clusters, with numerous and strong edges within clusters and few and weak edges between clusters. Formally, the clustering coefficient of a node is the ratio of the sum of the weights across all complete triangles around the node, to the number of edges connecting the node. Clustering coefficient is bounded by [0,1]. Modularity [Newman, 2006] is a more precise measure of functional segregation but is sensitive to communities of more complex configurations, with large number of edges within modules but small numbers of edges between modules. Modularity values are bounded by [0,1].

#### Mean betweenness

Mean betweenness [Freeman, 1979] is a measure of centrality. Betweeness of a node is defined as the fraction of all shortest paths in the network that pass through a given node. This measures the importance of nodes to overall network integrity. The mean betweenness [Van den Heuvel et al., 2010; Tijms et al., 2013] is a measure of overall network integrity. Formally, betweenness of a node is the sum of ratios of shortest paths traversing the node, versus the number of shortest paths in the whole network. Betweeness is bounded by [0,1].

#### Smallworldness

Smallworldness [Humphries and Gurney, 2008; Watts and Strogatz, 1998] is characterized by a high degree of clustering but each node can be reached easily by a low path length. It is typically used as a measure of “wiring‐efficiency” and is a structure found in many naturally occurring systems. Formally, smallworldness is the ratio of the normalized clustering coefficient vs. the normalized path length. The normalized versions of these two measurements are the original measure, normalized to the equivalent measure from a randomly generated network with the same density. The clustering coefficient of the random network was obtained using the analytic solution given by [Watts and Strogatz, 1998]. The mean path length of the random network was obtained using the analytic solution of Fronczak et al., [2004]. The final smallworldness value is bounded by [0,∞].

### Statistical Analysis

Subjects with PEs and without PEs were compared using a 1‐way ANCOVA model, covarying for gender, age, and handedness. Handedness data were obtained from previous measurements obtained from the ALSPAC data dictionary (details are available at: http://www.bris.ac.uk/alspac/researchers/data-access/data-dictionary).

Post‐hoc *t*‐tests were performed where significant *F*‐statistics were found. To correct for bias and instability in inferred group differences, and to address the multiple comparison problem, multithreshold permutation correction (MTPC) [Drakesmith et al., in press] was applied to the Fstatistics. This correction method is based on permutation based correction methods applied in more traditional neuroimaging analysis [Maris and Oostenveld, 2007; Nichols and Holmes, 2002; Smith and Nichols, 2009]. In this case, correction is applied by computing a distribution of null test statistics across thresholds. The full MTPC process is detailed below:

1. Apply thresholds, *τ*, to the networks, from 0 to *T* and compute GT metrics for all networks across all thresholds.
2. Compute test statistic (in this case *F*) on GT metrics with the correct group assignments for each threshold.
3. Permute the group assignments across *n* iterations and recompute test statistics for each permutation and each threshold, giving a distribution of null test statistics at each threshold.
4. Take the maximum test statistic across all thresholds for each permutation, resulting in one summarized null statistic for each permutation. To additionally correct for multiple comparisons of node‐level metrics across regions, take the maximum across nodes as well as thresholds.
5. Identify the critical value (denoted here as *F*_crit_) for the test‐statistic from the top *α*th percentile of the null test statistics, where *α* is the desired confidence level (e.g., 5%).
6. Identify clusters where the true test statistic is higher than the critical value for each threshold and compute the AUC for these clusters (denoted *A*_MTPC_). The peak statistic and the corresponding threshold are denoted *F*_MTPC_ and *τ*_MTPC_, respectively.
7. Compute a critical AUC from the mean of the super‐critical AUCs for the permuted tests (denoted *A*_crit_).
8. Reject the null hypothesis if the AUC of the significant clusters exceeds the critical AUC (*A*_MTPC_>*A*_crit_).

The 95th percentile of this distribution is treated as the critical value (*P* < 0.05). Any clusters of super‐critical *F*‐statistics exceeding a critical cluster size were treated as evidence of genuine group differences and lead to rejection of the null hypothesis. Correction was performed across thresholds of 0 to *m* = 20 streamlines and *n* = 500 permutations. In the case of node‐level statistics, correction for multiple comparisons was also performed by additionally taking the maximum of the null test statistic across regions as well as thresholds.

### Network‐Based Statistics

Network‐based statistics (NBS) [Zalesky et al., 2010] was used to test for local effects in the edges of the networks. This method performs a statistical test on connection‐by‐connection basis. All 248 connectivity matrices were concatenated and tested with the same design employed for the GT. NBS was run with an initial *F* threshold of 3.1 and 2,500 permutations.

### Analysis of Subnetworks

In addition to examining the topology of the whole‐brain network, the topologies of 23 structural subnetworks were also tested. This allowed us to examine how the structural connectivity underlying specific functional modes differs between groups, while excluding that of regions that do not show little or no involvement in the functional mode.

Twenty one subnetworks were obtained by masking the structural connectivity matrices with maps of temporally functional modes (TFMs) [Smith et al., 2012], downloaded from http://www.fmrib.ox.ac.uk/analysis/TFMs/. These maps identify spatially and temporally independent networks, which were derived using independent components analysis (ICA) from fMRI data. The mask was obtained by thresholding the TFMs at *z* > 4 and registering them to the AAL atlas. Any AAL regions with a proportional overlap of 50% or more with the thresholded TFM were deemed to be part of the TFM. This mask of regions was then used to derive a subnetwork of the full structural connectome. The AAL regions were not uniquely assigned to TFMs. TFM5 and TFM15 were excluded from the analysis as these networks are likely to be components related to physiological noise rather than true brain function [Smith et al., 2012].

In addition to the 21 TFMs, subnetworks defined by the DMN and the rich‐club network were also tested. The DMN was derived by the method described in Smith et al., [2012]: Each TFM was multiplied by the average activity in the posterior cingulate cortex across all TFMs and then summed across all TFMs. This was then masked in the same way as the TFNs. The rich‐club network was derived by selecting the same nodes reported to be part of the rich‐club network by Van den Heuvel and Sporns, [2011] and masked in the same way as the TFNs.

## RESULTS

### Network‐Level Topology

Of all network level measurements, density and mean efficiency showed significant differences following correction for instability and multiple comparisons (figure 2). Density (*F* = 7.843, *P* = 0.006, *P*_corr_ = 0.020 at *τ* = 10) and global efficiency (*F* = 7.981, *P* = 0.005, *P*_corr_ = 0.025 at *τ* = 4) were significantly reduced in the PE group compared to the control group. No significant effects were observed in mean betweenness (*F* = 3.219, *P* = 0.074 *P*_corr_ = 0.361), mean clustering coefficient (*F* = 5.012, *P* = 0.026, *P*_corr_ = 0.144), characteristic path length (*F* = 1.376, *P* = 0.242, *P*_corr_ = 0.569), and smallworldness (*F* = 1.376, *P* = 0.242, *P*_corr_ = 0.569) (see Supporting Information for full details of statistics).

When testing for differences in global efficiency across density thresholds, a smaller, but still significant, effect was observed (*F* = 4.849, *P* = 0.012, *P*_corr_ = 0.021 at *τ* = 0.08).

### Node‐Level Topology

**Figure 3:**
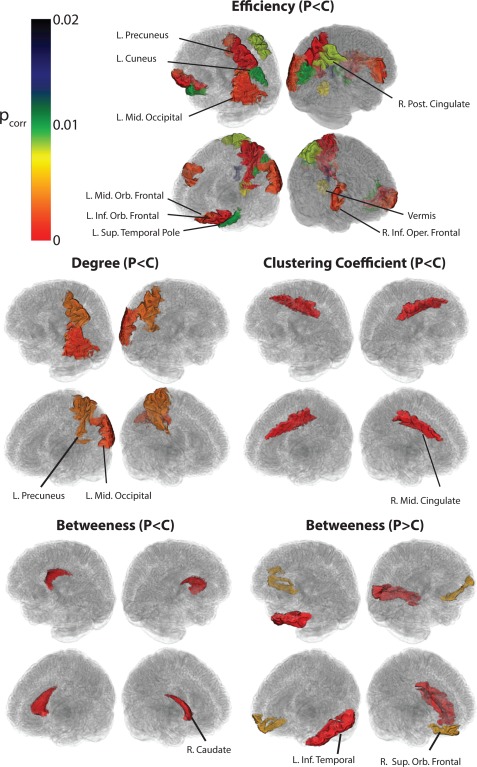
Results for node‐level analysis found significant at *P*_corr_ <0.05. Significant reductions (*P* < *C*) were found in efficiency, degree, clustering coefficient and betweenness. Significant increases (*P* < *C*) were found in betweenness. [Color figure can be viewed in the online issue, which is available at http://wileyonlinelibrary.com.]

Nodes showing significant effects in local network topology are illustrated in Figure 3 (Full results of all statistical tests are provided in Supporting Information). The PE group showed significant reductions in local efficiency of the left occipital lobe, cuneus and posterior cingulate gyrus and the right operculum. Reductions were also found in the left anterior temporal and inferior frontal regions. Reductions in node degree were identified in the left precuneus and occipital cortex. Clustering coefficient showed selective significant reductions in the right middle cingulate cortex. Betweeness was found to be reduced in the right caudate and cuneus but increased in the left inferior temporal and right orbitofrontal cortices.

### Network‐Based Statistics

NBS analysis did not show any significant effects in any network components.

### Topology of Subnetworks

**Figure 4:**
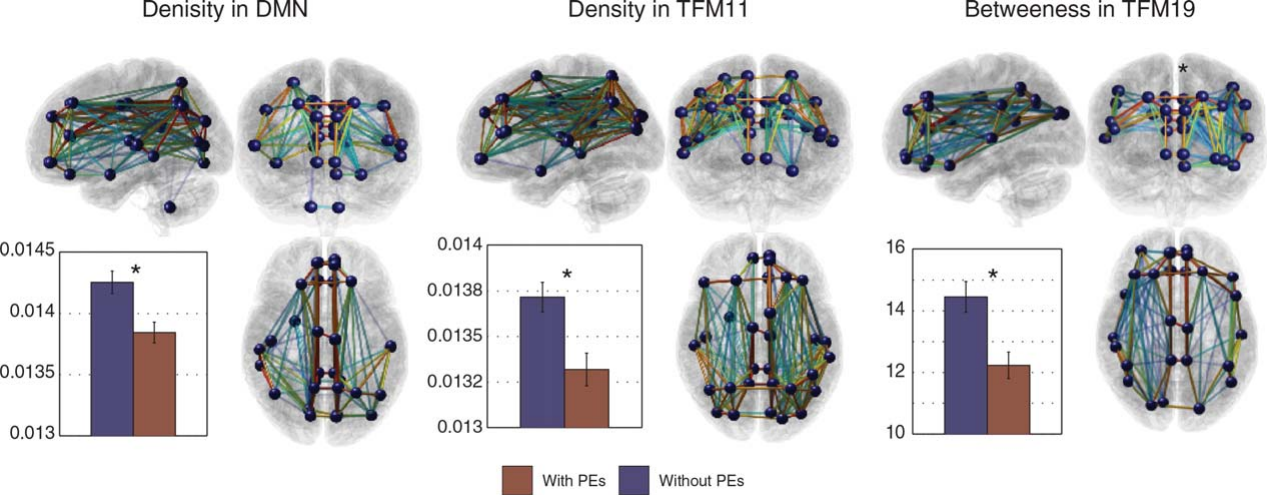
Subnetworks where significant effects were identified *P_corr_<0.05. Significant reductions in mean efficiency were identified in the DMN and TFM11. Significant reduction in mean betweenness was identified in TFM19 (see text for descriptions of these networks). [Color figure can be viewed in the online issue, which is available at http://wileyonlinelibrary.com.]

Results for significant differences in subnetwork topology are shown in Figure 4 (Full statistics for all subnetworks tested are provided in Supporting Information). The PE group showed significant reduction of density in the DMN (*F* = 9.226, *P* = 0.003, *P*_corr_ = 0.016) and TFM11 (*F* = 9.617, *P* = 0.002, *P*_corr_ = 0.002). There was also a significant reduction in mean betweenness in TFM19 (*F* = 12.325, *P* = 0.001, *P*_corr_ = 0.01). Both TFM11 and TFM19 have strong overlap with the DMN, comprising of particularly strong connections between cuneus, posterior cingulate and anterior cingulate cortices. All other subnetworks and the rich‐club network failed to show any significant effects.

## DISCUSSION

Dysconnectivity is a hypothesized factor in the development of schizophrenia and several previous neuroimaging studies have identified altered connectivity in schizophrenia patients [Pettersson‐Yeo et al., 2011; Wheeler and Voineskos, 2014]. It is likely that symptoms of the disorder are preceded by underlying structural abnormalities [Fusar‐Poli et al., 2013]. This opens the possibility of using structural connectivity as a predictive biomarker for future development of psychosis. In this study, we have identified differences in the topology of structural brain networks in individuals who have PEs but have not sought any clinical intervention. Such an approach eliminates the bias that occurs when sampling groups from schizophrenia patients or clinical high‐risk groups and enables the investigation of the core features of psychosis without contamination from effects of treatment and disease complications. Using GT, it is possible to elucidate more subtle connectomic alterations which may be too subtle to be detected using more traditional techniques (see Supporting Information of an analysis conducted on this sample using standard Diffusion tensor imaging (DTI) methods).

Decreased network density and efficiency indicates there is a diffuse nonspecific reduction of connectivity in the brains of individuals with PEs, suggesting that the axonal pathways connecting brain regions is likely impaired. These results conform to previous results for schizophrenia patients. In particular, Zalesky et al., [2011] also showed reduced density and mean efficiency in schizophrenia. The structural alterations in these pathways may have a causal effect on the mediated functional interactions [Honey et al., 2010]. This can have implications for potential functional changes at the network‐level.

Of particular note is that there are still detectable differences in global efficiency when density is kept constant across subjects. Changes in density are known to lead to changes to global efficiency [Ginestet et al., 2011; Van Wijk et al., 2010]. Our results indicate there is some additional network restructuring in connectomes of individuals with PEs which cannot be explained solely by a general reduction of connectivity.

Regions showing reduced node‐level efficiency in the present study overlap with many regions previously implicated by Van den Heuvel et al., [2010]. Of particular importance is the reduction of local efficiency in the posterior cingulate and parietal regions. Several other studies have identified these regions as key hubs of the “structural core” of the healthy human connectome [Gong et al., 2009; Hagmann et al., 2008; Van den Heuvel and Sporns, 2011]. The reduction of efficiency of structural connections to and from these regions will have a particularly strong impact on the polysynaptic interactions mediated by these connections. This is likely to lead to high levels of dysfunction throughout the rest of the brain. Another interesting parallel is a study of 22q11 deletion syndrome [Ottet et al., 2013]. Although this study failed to find any significant effects in local GT metrics, it did find negative correlations between severity of auditory hallucinations and local efficiency in the same left inferior frontal regions implicated in the present study. Alterations of the white‐matter of language pathways have been implicated in auditory hallucinations, particularly those projecting to the inferior frontal and anterior temporal cortices [Allen and Modinos, 2012; Catani et al., 2011; Seok et al., 2007; Shergill et al., 2007]. It is possible that alterations in the routing through these language systems contributes to the auditory hallucinations experienced by individuals with PEs. In our sample, the largest contributors to PEs were those associated with auditory hallucinations (see Supporting Information).

Betweenness centrality is higher in the PE group in a number of regions but lower in others. This is in contrast to [Van den Heuvel et al., 2010], who showed consistently reduced betweenness centrality in a number of regions. It is likely that the networks undergo alterations during the course of the illness, such as reduction in network integration in the network hubs. These changes will alter the routes required for optimal information transfer, causing some regions to become more or less integral (manifesting in increases or decreases in betweenness centrality). In our results, only the caudate shows decreased betweenness centrality in the PE group, which suggests a restructuring of the network such that there is decreased reliance on subcortical hubs such as the caudate. Similar patterns of change observed for clustering coefficient also suggests that the local topology around these regions has also changed. Again, it is likely that these measurements will vary as different network components become impaired at different levels of disease severity. The alterations are consistent with growing consensus that a prominent feature of schizophrenia is “hubopathy” [Rubinov and Bullmore, 2013].

In our analysis of functionally independent subnetworks, we identified structural connectomes underpinning particular functional states. This includes networks associated with language, perceptual and motor processing. Selecting networks derived from an ICA ensures the subnetworks tested are optimized for their functional independence, without relying on a priori assumptions of the structure of networks subserving different functions. We found generally, most functional subnetworks did not show differences between individuals with PEs and controls. However, the subnetwork comprising the DMN showed reduced density. The DMN has been the subject of much interest in the psychopathology literature, particularly regarding functional connectivity. Lack of suppression of the DMN is associated with hallucinations and faulty self‐processing [Van der Meer et al., 2010; Whitfield‐Gabrieli and Ford, 2012], hallmarks of psychosis and dysfunction in this system is a prime candidate for core disturbances which are early precursors to psychosis [Brent et al., 2014]. We compliment this, for the first time, with structural connectivity data from young people with PEs showing reduced density which may in turn lead to reduced functional connectivity. As noted previously regarding the local efficiency, the DMN comprises several core hubs of the healthy connectome and, therefore, altered topology in this subnetwork confers a particular risk of dysfunction. Having shown that network topology is impaired in specific subnetworks, further analysis of subnetworks in patients who develop full psychosis may implicate a more extensive set of subnetworks. Nevertheless, we would speculate that it is this combination of subnetwork dysfunction in core midline structures underlying self‐related processes with more diffuse changes likely to compromise cognitive efficiency, subtly, [Meier et al., 2014; Woodberry et al., 2008; Zammit et al., 2004] that then leads to the cluster of experiences that constitutes psychosis. In future work we aim to elucidate further the functional consequences of the topological impairments in this group, via cognitive testing and functional neuroimaging.

NBS is an approach to statistical analysis analogous to statistical parametric mapping of volumetric data. In NBS, the statistical mapping is performed across the edges of the network. This analysis failed to identify any significant effects on individual connections, indicating that the changes that occur in psychosis are distributed throughout the network and are too subtle to be detected by analysis of pair‐wise connections. Our results contrast with previous NBS analyses on schizophrenia patients [Zalesky et al., 2011], which found connections comprising the inter‐hemispheric occipital and parietal connections and parietofrontal connections were significantly impaired. Despite this discrepancy, the parietal component of this network overlaps significantly with the regions showing reduced local efficiency. It is, therefore, likely that the reduction in efficiency is a milder variant of the more severe structural abnormalities found in schizophrenia patients.

The work presented here is unique in a number of ways. This is the first investigation of structural brain topology in PEs in a population sample with a substantial sample size with population‐based controls (122 PEs, 125 controls). This study also applies a rigorous statistical correction of the GT metrics to ensure that any genuine group differences in network topology are identified robustly. Thresholding can have a large impact on the inferred topology and how topologies of different groups compare [Drakesmith et al., in press].

In contrast to previous MRI‐based studies of structural network topology that use the single tensor model for tractography (with the notable exception of the recent study by Griffa et al. [in press], who used diffusion spectrum imaging) we used a spherical deconvolution approach. The diffusion tensor model is incapable of resolving more than one fiber orientation within a voxel, and thus, can only reconstruct one fiber pathway through a voxel. Given that 90% of voxels contain crossing fibers [Jeurissen et al., 2013] this is a severe limitation that will likely lead to large numbers of false negative/false positive edges, biasing the GT analysis. Here, we employed the damped Richardson‐Lucy algorithm [Dell'acqua et al., 2010] which is able to resolve crossing fiber populations, allowing more complete and robust recovery of fiber trajectories, and therefore, of network topologies. This difference in approaches may explain the differences between our observations on node‐level connectivity and those reported in previous studies of schizophrenia.

A difficult issue that persists with graph theoretical analysis is the large difference in acquisition and analysis pipelines. The high dependency of GT metrics on these parameters makes comparisons of GT findings from different studies difficult. This raises a similar issue in the literature that concern more established FA imaging. Like FA, GT appears to have high sensitivity to conditions but often lack specificity [Griffa et al., 2013]. Further standardization of pipelines and more robust statistical testing may improve the specificity of GT analysis. However, making wider inferences on GT analysis should always be done with caution.

Finally, as with studies that compare clinical groups with controls, there are factors that could potentially confound or explain the relationship between PEs and imaging measures in our nonclinical cohort, such a substance misuse, genetic factors, and cognitive ability, and these should be the subject of further research.

## CONCLUSIONS

Our work is the first to demonstrate alterations in network topology in a population of individuals with PEs specifically identified from a population‐based cohort rather than in people seeking help from clinical services. Such experiences confer increased risk of developing a clinical disorder. The changes are diffuse across the network and too subtle to be detected when looking at individual network components but are detectable when exploring network topology. Network‐wide reductions in density and efficiency were detected as well as numerous regional alterations. The alterations in the structural connections, particularly in core network hubs, are likely to contribute to significant dysfunction throughout the brain. The findings indicate that topology of structural connectomes is a potential biomarker for the development of clinical psychosis.

## CONFLICTS OF INTEREST

None of the authors have any conflicts of interest to declare.

## Supporting information

Supporting Information

Supporting Information
