# Supplementary material for: Schizophrenia‐like topological changes in the structural connectome of individuals with subclinical psychotic experiences
Source: Hum Brain Mapp. 2015 Apr 2;36(7):2629–43. doi: 10.1002/hbm.22796 (PMC4479544; doi:10.1002/hbm.22796)
Supplement: Supplementary file 1 — Supporting Information [file HBM-36-2629-s001.pdf]

Significant GT metrics (network-level metrics)

	GT METRIC	PE descriptives		Control descriptives		ANOVA results		MTPC results					Post-hoc t-tests		pseudo-z sco
		Mean	SE	Mean	SE	F	p	F crit	P corr	tau	AUC	AUCcrit	t	p	
	Density	0.064	0.000	0.065	0.000	7.843	0.006	5.909	0.020	10	14.324	9.005	-2.499	0.013	-2.326
	Global Efficiency (thresho	0.383	0.002	0.389	0.001	7.981	0.005	6.548	0.025	4	6.970	7.394	-2.613	0.010	-2.241
	Global Efficiency (thresho	0.368	0.003	0.370	0.003	6.427	0.012	4.849	0.021	0.08	0.0039	0.0013	-0.731	0.466	-2.308

Significant GT metrics (node-level metrics)

AAL REGION	GT METRIC	PE descriptives		Control descriptives		ANOVA results		MTPC results					Post-hoc t-tests		pseudo-z sco
		Mean	SE	Mean	SE	F	p	F crit	P corr	tau	AUC	AUCcrit	t	p	
Occipital_Mid_L'	Degree	15.293	0.215	16.432	0.252	13.322	0.000	7.609	0.002	4	26.346	7.237	-3.437	0.001	-3.143
Precuneus_L'	Degree	15.764	0.248	16.808	0.225	11.240	0.001	7.609	0.003	14	27.016	3.196	-3.118	0.002	-2.935
Frontal_Mid_Orb_L'	Efficiency	0.303	0.003	0.316	0.002	11.181	0.001	7.653	0.002	14	10.230	6.610	-3.208	0.002	-3.143
Frontal_Inf_Oper_R'	Efficiency	0.360	0.002	0.370	0.002	12.739	0.000	7.653	0.002	8	17.330	5.549	-3.438	0.001	-3.143
Frontal_Inf_Orb_L'	Efficiency	0.383	0.002	0.394	0.002	14.687	0.000	7.653	0.000	4	46.408	4.644	-3.708	0.000	-Inf
Cingulum_Post_R'	Efficiency	0.355	0.002	0.364	0.002	9.391	0.002	7.653	0.017	12	5.569	4.126	-2.756	0.006	-2.393
Cuneus_L'	Efficiency	0.355	0.003	0.366	0.003	10.104	0.002	7.653	0.010	12	8.876	5.780	-2.867	0.005	-2.575
Occipital_Mid_L'	Efficiency	0.450	0.002	0.462	0.003	14.856	0.000	7.653	0.002	4	31.040	5.525	-3.511	0.001	-3.143
Parietal_Sup_R'	Efficiency	0.408	0.002	0.417	0.002	9.414	0.002	7.653	0.008	12	4.646	1.815	-2.803	0.005	-2.638
Precuneus_L'	Efficiency	0.494	0.002	0.507	0.002	16.596	0.000	7.653	0.000	6	73.328	2.082	-3.876	0.000	-Inf
Temporal_Pole_Sup_L'	Efficiency	0.325	0.005	0.341	0.003	8.964	0.003	7.653	0.010	10	7.584	5.048	-2.872	0.004	-2.575
Vermis_4_5'	Efficiency	0.108	0.009	0.149	0.009	10.824	0.001	7.653	0.007	18	8.308	4.428	-3.315	0.001	-2.712
Frontal_Sup_Orb_R'	Betweenness	359.623	20.011	270.877	15.845	10.939	0.001	7.825	0.005	10	10.604	9.306	3.483	0.001	2.806
Caudate_R'	Betweenness	83.233	8.242	144.370	14.234	13.275	0.000	7.825	0.000	8	24.475	8.858	-3.702	0.000	-Inf
Temporal_Inf_L'	Betweenness	314.046	19.667	229.371	17.269	12.360	0.001	7.825	0.000	0	8.771	2.102	3.238	0.001	Inf
Cingulum_Mid_R'	Clustering Coefficient	0.466	0.005	0.494	0.005	16.153	0.000	8.293	0.000	2	45.384	6.009	-3.916	0.000	-Inf

Significant GT metrics (subn 06/03/2015

SUB-NETWORK	GT METRIC	PE descriptives		Control descriptives		ANOVA results		MTPC results					Post-hoc t-tests		pseudo-z sco
		Mean	SE	Mean	SE	F	p	F crit	P corr	tau	AUC	AUCcrit	t	p	
DMN	Density	.014	8.4525031126	.014	9.1837850570	9.226	.003	6.325	.016	4	10.961	9.712	-3.297	.001	-2.409
TFM11	Density	.013	8.9959917677	.014	8.1738818896	9.617	.002	5.714	.002	6	22.319	10.769	-3.272	.001	-3.090
TFM19	Mean Betweeness	17.202	.498	19.994	.555	12.325	.001	8.157	.010	8	14.836	4.101	-3.742	.000	-2.576

re

re

re

Significant GT metrics (per-node metrics)		SIGNIFICANT RESULTS (p<0.05) IN BOLD												
**GT METRIC**	**PE descriptives**		**Control descriptives**		**ANOVA results**		**MTPC results**					**Post-hoc t-tests**		**pseudo-z sco**
	**Mean**	**SE**	**Mean**	**SE**	**F**	**p**	**F crit**	**P corr**	**tau (stramlin**	**AUC**	**AUCcrit**	**t**	**p**	
Characteristic Path Length	2.779	0.0259	2.727	0.0332	1.376	0.242	6.202	0.569	2					0.569
Mean Betweenness	171.590	1.0564	168.968	1.1341	3.219	0.074	7.134	0.361	0					0.913
Smallworldness	6.611	0.0962	6.478	0.1015	1.285	0.258	6.420	0.775	18					0.285
Mean Clustering Coefficient	0.453	0.0017	0.459	0.0019	5.012	0.026	7.079	0.144	4					-1.463
**Density**	**0.064**	**0.0004**	**0.065**	**0.0003**	**7.843**	**0.006**	**5.909**	**0.020**	**10**	**14.324**	**9.005**	**-2.499**	**0.013**	**-2.326**
**Global Efficiency**	**0.383**	**0.0016**	**0.389**	**0.0012**	**7.981**	**0.005**	**6.548**	**0.025**	**4**	**6.970**	**7.394**	**-2.613**	**0.010**	**-2.241**
									**tau (density)**					
**Global Efficiency (thresholded by density)**	**0.368**	**0.0026**	**0.370**	**0.0030**	**6.427**	**0.012**	**4.849**	**0.021**	**0.08**	**0.0039**	**0.0013**	**-0.731**	**0.466**	**-2.308**

ore

Significant GT metrics (per-node metrics) **SIGNIFICANT RESULTS (p<0.05) IN BOLD**

AAL REGION	GT METRIC	PE descriptives		Control descriptives		ANOVA results		MTPC results					Post-hoc t-tests		pseudo-z score
		Mean	SE	Mean	SE	F	p	F crit	P corr	tau	AUC	AUCcrit	t	p	
Precentral_L'	Degree	7.463	0.181	7.648	0.179	0.804	0.371	7.609	0.825	20					-0.221
Precentral_R'	Degree	8.285	0.176	7.824	0.195	3.161	0.077	7.609	0.257	14					1.133
Frontal_Sup_L'	Degree	15.065	0.205	15.632	0.234	3.984	0.047	7.609	0.204	4					-1.271
Frontal_Sup_R'	Degree	20.390	0.249	20.024	0.250	2.137	0.145	7.609	0.434	2					0.782
Frontal_Sup_Orb_L'	Degree	6.813	0.178	7.088	0.162	1.189	0.277	7.609	0.721	10					-0.357
Frontal_Sup_Orb_R'	Degree	9.008	0.215	8.128	0.192	9.289	0.003	7.609	0.003	10	2.679	5.392			2.935
Frontal_Mid_L'	Degree	8.528	0.213	8.920	0.187	1.678	0.196	7.609	0.489	14					-0.692
Frontal_Mid_R'	Degree	7.187	0.171	7.384	0.161	0.718	0.398	7.609	0.833	20					-0.211
Frontal_Mid_Orb_L'	Degree	4.602	0.115	5.072	0.114	9.016	0.003	7.609	0.020	16	5.031	6.653			-2.326
Frontal_Mid_Orb_R'	Degree	9.862	0.260	9.664	0.267	0.585	0.445	7.609	0.888	0					0.141
Frontal_Inf_Oper_L'	Degree	9.122	0.156	9.320	0.145	1.199	0.275	7.609	0.721	10					-0.357
Frontal_Inf_Oper_R'	Degree	9.764	0.191	10.408	0.193	5.965	0.015	7.609	0.082	4					-1.740
Frontal_Inf_Tri_L'	Degree	7.650	0.192	8.224	0.195	4.339	0.038	7.609	0.124	20					-1.540
Frontal_Inf_Tri_R'	Degree	10.585	0.207	10.784	0.171	0.628	0.429	7.609	0.866	6					-0.168
Frontal_Inf_Orb_L'	Degree	6.350	0.172	7.040	0.168	8.645	0.004	7.609	0.015	10	0.943	7.683			-2.432
Frontal_Inf_Orb_R'	Degree	7.138	0.178	8.016	0.202	10.243	0.002	7.609	0.010	8	4.834	11.707			-2.575
Rolandic_Oper_L'	Degree	11.805	0.178	12.056	0.162	1.563	0.212	7.609	0.591	0					-0.537
Rolandic_Oper_R'	Degree	8.959	0.182	9.312	0.171	2.000	0.159	7.609	0.513	2					-0.655
Supp_Motor_Area_L'	Degree	17.992	0.244	18.272	0.236	1.366	0.244	7.609	0.619	0					-0.497
Supp_Motor_Area_R'	Degree	10.431	0.229	9.944	0.211	2.439	0.120	7.609	0.334	12					0.966
Olfactory_L'	Degree	4.691	0.113	4.200	0.124	8.444	0.004	7.609	0.028	6	0.842	5.196			2.192
Olfactory_R'	Degree	7.146	0.214	6.576	0.203	4.131	0.043	7.609	0.165	0					1.388
Frontal_Sup_Medial_L'	Degree	11.634	0.226	11.816	0.192	0.677	0.412	7.609	0.836	16					-0.207
Frontal_Sup_Medial_R'	Degree	10.943	0.178	11.304	0.206	1.354	0.246	7.609	0.663	8					-0.436
Frontal_Med_Orb_L'	Degree	4.098	0.153	4.384	0.140	2.372	0.125	7.609	0.429	20					-0.791
Frontal_Med_Orb_R'	Degree	4.862	0.138	4.504	0.140	3.637	0.058	7.609	0.259	16					1.129
Rectus_L'	Degree	8.171	0.176	8.528	0.183	2.094	0.149	7.609	0.484	2					-0.700
Rectus_R'	Degree	4.065	0.146	4.168	0.126	0.256	0.613	7.609	0.990	10					-0.013
Insula_L'	Degree	12.268	0.254	11.448	0.227	5.441	0.020	7.609	0.088	6					1.703
Insula_R'	Degree	17.431	0.256	17.112	0.269	0.662	0.417	7.609	0.875	2					0.158
Cingulum_Ant_L'	Degree	9.154	0.166	9.504	0.183	2.320	0.129	7.609	0.427	10					-0.794
Cingulum_Ant_R'	Degree	9.220	0.157	8.944	0.179	1.049	0.307	7.609	0.803	10					0.249
Cingulum_Mid_L'	Degree	8.374	0.105	8.824	0.111	7.495	0.007	7.609	0.045	12					-2.004
Cingulum_Mid_R'	Degree	14.545	0.158	14.248	0.152	2.009	0.158	7.609	0.528	2					0.632
Cingulum_Post_L'	Degree	17.033	0.277	16.840	0.240	0.325	0.569	7.609	0.978	0					0.027
Cingulum_Post_R'	Degree	6.528	0.145	7.024	0.170	5.893	0.016	7.609	0.062	12					-1.868
Hippocampus_L'	Degree	12.276	0.255	11.920	0.279	0.851	0.357	7.609	0.760	4					0.306
Hippocampus_R'	Degree	9.780	0.218	10.248	0.232	2.051	0.153	7.609	0.446	6					-0.763
ParaHippocampal_L'	Degree	3.951	0.161	4.296	0.163	2.783	0.097	7.609	0.327	16					-0.980
ParaHippocampal_R'	Degree	5.520	0.171	5.800	0.163	1.800	0.181	7.609	0.514	8					-0.652
Amygdala_L'	Degree	9.106	0.283	8.392	0.238	3.707	0.055	7.609	0.222	0					1.221
Amygdala_R'	Degree	2.837	0.120	2.704	0.107	0.605	0.437	7.609	0.896	12					0.130

Calcarine_L'	Degree	14.537	0.321	14.984	0.330	0.936	0.334	7.609	0.738	2					-0.335
Calcarine_R'	Degree	6.317	0.161	6.912	0.162	7.542	0.006	7.609	0.023	16					-2.267
Cuneus_L'	Degree	6.707	0.170	7.248	0.191	5.110	0.025	7.609	0.092	10					-1.686
Cuneus_R'	Degree	14.805	0.310	15.232	0.346	1.301	0.255	7.609	0.603	0					-0.521
Lingual_L'	Degree	8.553	0.204	8.848	0.230	1.397	0.238	7.609	0.611	18					-0.509
Lingual_R'	Degree	11.593	0.240	11.888	0.250	1.186	0.277	7.609	0.688	8					-0.402
Occipital_Sup_L'	Degree	14.537	0.269	15.288	0.240	4.130	0.043	7.609	0.135	2					-1.494
Occipital_Sup_R'	Degree	9.911	0.220	10.368	0.213	2.142	0.145	7.609	0.417	12					-0.811
**Occipital_Mid_L'**	**Degree**	**15.293**	**0.215**	**16.432**	**0.252**	**13.322**	**0.000**	**7.609**	**0.002**	**4**	**26.346**	**7.237**	**-3.437**	**0.001**	**-3.143**
Occipital_Mid_R'	Degree	12.626	0.266	12.992	0.213	1.595	0.208	7.609	0.553	2					-0.594
Occipital_Inf_L'	Degree	7.049	0.172	6.744	0.157	1.122	0.291	7.609	0.718	8					0.361
Occipital_Inf_R'	Degree	4.528	0.123	4.896	0.137	4.294	0.039	7.609	0.172	18					-1.366
Fusiform_L'	Degree	7.179	0.189	7.792	0.206	5.243	0.023	7.609	0.083	10					-1.731
Fusiform_R'	Degree	7.138	0.192	7.344	0.160	1.218	0.271	7.609	0.674	16					-0.420
Postcentral_L'	Degree	8.122	0.156	8.264	0.152	0.451	0.503	7.609	0.947	20					-0.067
Postcentral_R'	Degree	18.846	0.214	19.448	0.236	3.239	0.073	7.609	0.269	0					-1.106
Parietal_Sup_L'	Degree	20.545	0.333	21.056	0.337	1.320	0.252	7.609	0.638	0					-0.471
Parietal_Sup_R'	Degree	10.846	0.199	11.448	0.202	5.864	0.016	7.609	0.090	14					-1.695
Parietal_Inf_L'	Degree	11.772	0.235	12.120	0.240	0.898	0.344	7.609	0.755	0					-0.313
Parietal_Inf_R'	Degree	8.423	0.212	8.688	0.232	0.744	0.389	7.609	0.821	0					-0.226
SupraMarginal_L'	Degree	8.756	0.178	8.392	0.173	3.298	0.071	7.609	0.202	2					1.276
SupraMarginal_R'	Degree	6.984	0.151	6.624	0.139	3.650	0.057	7.609	0.185	10					1.325
Angular_L'	Degree	7.187	0.158	7.360	0.158	1.079	0.300	7.609	0.763	4					-0.302
Angular_R'	Degree	8.089	0.171	8.456	0.187	1.976	0.161	7.609	0.417	12					-0.811
**Precuneus_L'**	**Degree**	**15.764**	**0.248**	**16.808**	**0.225**	**11.240**	**0.001**	**7.609**	**0.003**	**14**	**27.016**	**3.196**	**-3.118**	**0.002**	**-2.935**
Precuneus_R'	Degree	19.203	0.274	19.640	0.249	1.860	0.174	7.609	0.529	6					-0.629
Paracentral_Lobule_L'	Degree	10.276	0.207	10.472	0.183	0.565	0.453	7.609	0.891	2					-0.136
Paracentral_Lobule_R'	Degree	4.764	0.137	5.344	0.113	10.251	0.002	7.609	0.008	20	2.718	3.525			-2.638
Caudate_L'	Degree	7.358	0.243	7.984	0.245	3.381	0.067	7.609	0.222	10					-1.221
Caudate_R'	Degree	10.065	0.242	10.976	0.266	6.102	0.014	7.609	0.050	4					-1.959
Putamen_L'	Degree	17.236	0.320	17.688	0.280	0.963	0.327	7.609	0.728	8					-0.348
Putamen_R'	Degree	15.642	0.291	16.136	0.233	2.268	0.133	7.609	0.389	16					-0.861
Pallidum_L'	Degree	2.821	0.122	3.008	0.125	1.453	0.229	7.609	0.666	10					-0.431
Pallidum_R'	Degree	2.252	0.109	2.136	0.108	0.904	0.343	7.609	0.790	20					0.267
Thalamus_L'	Degree	6.805	0.225	7.320	0.241	2.164	0.143	7.609	0.417	18					-0.811
Thalamus_R'	Degree	6.911	0.212	7.240	0.228	1.209	0.273	7.609	0.673	20					-0.422
Heschl_L'	Degree	1.130	0.068	1.280	0.070	2.278	0.133	7.609	0.544	12					-0.606
Heschl_R'	Degree	1.049	0.047	1.152	0.052	2.732	0.100	7.609	0.434	10					-0.782
Temporal_Sup_L'	Degree	7.179	0.186	7.624	0.186	3.610	0.059	7.609	0.217	16					-1.234
Temporal_Sup_R'	Degree	12.431	0.234	12.744	0.197	1.514	0.220	7.609	0.574	2					-0.562
Temporal_Pole_Sup_L'	Degree	6.000	0.218	6.824	0.210	7.905	0.005	7.609	0.032	10	0.079	5.262			-2.148
Temporal_Pole_Sup_R'	Degree	7.260	0.225	7.048	0.203	0.480	0.489	7.609	0.945	4					0.069
Temporal_Mid_L'	Degree	10.138	0.214	10.560	0.209	1.937	0.165	7.609	0.431	20					-0.788
Temporal_Mid_R'	Degree	12.829	0.265	13.304	0.272	2.047	0.154	7.609	0.392	4					-0.855
Temporal_Pole_Mid_L'	Degree	6.154	0.180	6.344	0.173	0.370	0.543	7.609	0.963	16					-0.046
Temporal_Pole_Mid_R'	Degree	7.789	0.204	8.528	0.201	7.414	0.007	7.609	0.040	10					-2.053

Temporal_Inf_L'	Degree	8.780	0.214	8.392	0.201	2.027	0.156	7.609	0.399	20					0.843
Temporal_Inf_R'	Degree	9.244	0.211	9.304	0.210	0.349	0.555	7.609	0.957	12					-0.054
Cerebelum_Crus1_L'	Degree	10.618	0.249	11.160	0.242	3.188	0.075	7.609	0.247	4					-1.157
Cerebelum_Crus1_R'	Degree	5.122	0.161	5.840	0.194	8.411	0.004	7.609	0.027	10	0.870	4.767			-2.216
Cerebelum_Crus2_L'	Degree	12.602	0.232	12.248	0.219	0.862	0.354	7.609	0.805	0					0.247
Cerebelum_Crus2_R'	Degree	13.463	0.250	14.104	0.243	3.433	0.065	7.609	0.215	0					-1.239
Cerebelum_3_L'	Degree	0.301	0.053	0.480	0.068	4.019	0.046	7.609	0.167	16					-1.382
Cerebelum_3_R'	Degree	0.179	0.040	0.288	0.057	3.001	0.084	7.609	0.284	20					-1.072
Cerebelum_4_5_L'	Degree	6.610	0.191	7.224	0.200	4.104	0.044	7.609	0.179	4					-1.345
Cerebelum_4_5_R'	Degree	0.805	0.080	1.056	0.099	3.429	0.065	7.609	0.227	20					-1.208
Cerebelum_6_L'	Degree	9.390	0.267	9.928	0.231	2.713	0.101	7.609	0.366	2					-0.905
Cerebelum_6_R'	Degree	4.894	0.196	5.144	0.162	1.845	0.176	7.609	0.521	8					-0.642
Cerebelum_7b_L'	Degree	3.024	0.141	3.368	0.133	3.105	0.079	7.609	0.255	18					-1.137
Cerebelum_7b_R'	Degree	9.000	0.204	9.424	0.198	2.529	0.113	7.609	0.371	0					-0.895
Cerebelum_8_L'	Degree	10.992	0.219	10.568	0.248	1.123	0.290	7.609	0.736	2					0.337
Cerebelum_8_R'	Degree	7.146	0.192	7.592	0.188	3.517	0.062	7.609	0.194	14					-1.300
Cerebelum_9_L'	Degree	2.024	0.088	1.928	0.087	0.353	0.553	7.609	0.977	20					0.029
Cerebelum_9_R'	Degree	6.447	0.217	7.000	0.221	3.731	0.055	7.609	0.194	0					-1.300
Cerebelum_10_L'	Degree	7.106	0.174	7.736	0.185	6.443	0.012	7.609	0.047	0					-1.989
Cerebelum_10_R'	Degree	5.350	0.158	5.584	0.174	1.114	0.292	7.609	0.768	0					-0.295
Vermis_1_2'	Degree	0.195	0.041	0.160	0.037	0.440	0.508	7.609	0.972	18					0.036
Vermis_3'	Degree	0.439	0.079	0.488	0.082	0.537	0.464	7.609	0.925	6					-0.094
Vermis_4_5'	Degree	12.821	0.276	14.032	0.271	8.537	0.004	7.609	0.020	0	0.889	11.608			-2.326
Vermis_6'	Degree	4.984	0.261	5.576	0.236	2.911	0.089	7.609	0.357	0					-0.921
Vermis_7'	Degree	2.285	0.165	2.608	0.163	2.948	0.087	7.609	0.284	6					-1.072
Vermis_8'	Degree	8.122	0.245	8.552	0.217	1.683	0.196	7.609	0.598	0					-0.528
Vermis_9'	Degree	2.309	0.127	1.976	0.116	2.694	0.102	7.609	0.361	8					0.914
Vermis_10'	Degree	1.220	0.093	1.408	0.108	2.755	0.098	7.609	0.316	14					-1.004
Precentral_L'	Efficiency	0.336	0.002	0.341	0.002	2.086	0.150	7.653	0.459	16					-0.740
Precentral_R'	Efficiency	0.477	0.002	0.479	0.002	0.771	0.381	7.653	0.871	0					-0.162
Frontal_Sup_L'	Efficiency	0.453	0.002	0.459	0.002	5.925	0.016	7.653	0.075	4					-1.780
Frontal_Sup_R'	Efficiency	0.435	0.002	0.441	0.002	5.065	0.025	7.653	0.107	8					-1.613
Frontal_Sup_Orb_L'	Efficiency	0.341	0.003	0.348	0.003	3.103	0.079	7.653	0.262	10					-1.121
Frontal_Sup_Orb_R'	Efficiency	0.449	0.003	0.441	0.003	4.245	0.040	7.653	0.149	2					1.445
Frontal_Mid_L'	Efficiency	0.347	0.003	0.354	0.002	3.574	0.060	7.653	0.215	14					-1.239
Frontal_Mid_R'	Efficiency	0.437	0.002	0.433	0.002	1.071	0.302	7.653	0.738	2					0.335
**Frontal_Mid_Orb_L'**	**Efficiency**	**0.303**	**0.003**	**0.316**	**0.002**	**11.181**	**0.001**	**7.653**	**0.002**	**14**	**10.230**	**6.610**	**-3.208**	**0.002**	**-3.143**
Frontal_Mid_Orb_R'	Efficiency	0.350	0.004	0.355	0.002	1.646	0.201	7.653	0.489	8					-0.692
Frontal_Inf_Oper_L'	Efficiency	0.351	0.002	0.355	0.002	2.048	0.154	7.653	0.501	12					-0.673
**Frontal_Inf_Oper_R'**	**Efficiency**	**0.360**	**0.002**	**0.370**	**0.002**	**12.739**	**0.000**	**7.653**	**0.002**	**8**	**17.330**	**5.549**	**-3.438**	**0.001**	**-3.143**
Frontal_Inf_Tri_L'	Efficiency	0.394	0.003	0.399	0.002	3.234	0.073	7.653	0.230	6					-1.199
Frontal_Inf_Tri_R'	Efficiency	0.390	0.002	0.395	0.002	3.493	0.063	7.653	0.249	6					-1.153
**Frontal_Inf_Orb_L'**	**Efficiency**	**0.383**	**0.002**	**0.394**	**0.002**	**14.687**	**0.000**	**7.653**	**0.000**	**4**	**46.408**	**4.644**	**-3.708**	**0.000**	**-Inf**
Frontal_Inf_Orb_R'	Efficiency	0.357	0.003	0.368	0.002	9.608	0.002	7.653	0.007	8	2.598	6.297			-2.712
Rolandic_Oper_L'	Efficiency	0.347	0.002	0.353	0.002	4.377	0.037	7.653	0.170	8					-1.371
Rolandic_Oper_R'	Efficiency	0.355	0.002	0.360	0.002	4.315	0.039	7.653	0.172	8					-1.366

Supp_Motor_Area_L'	Efficiency	0.502	0.002	0.507	0.002	4.713	0.031	7.653	0.117	0					-1.568
Supp_Motor_Area_R'	Efficiency	0.351	0.003	0.347	0.002	0.478	0.490	7.653	0.928	18					0.090
Olfactory_L'	Efficiency	0.221	0.007	0.208	0.008	2.058	0.153	7.653	0.541	20					0.611
Olfactory_R'	Efficiency	0.207	0.008	0.195	0.008	1.298	0.256	7.653	0.760	16					0.306
Frontal_Sup_Medial_L'	Efficiency	0.408	0.003	0.415	0.002	5.130	0.024	7.653	0.142	12					-1.469
Frontal_Sup_Medial_R'	Efficiency	0.407	0.002	0.416	0.002	7.968	0.005	7.653	0.023	8	0.237	6.777			-2.267
Frontal_Med_Orb_L'	Efficiency	0.350	0.004	0.357	0.002	2.688	0.102	7.653	0.329	8					-0.976
Frontal_Med_Orb_R'	Efficiency	0.443	0.002	0.436	0.002	5.035	0.026	7.653	0.119	0					1.561
Rectus_L'	Efficiency	0.305	0.002	0.310	0.003	3.297	0.071	7.653	0.295	12					-1.046
Rectus_R'	Efficiency	0.311	0.004	0.317	0.002	2.010	0.158	7.653	0.528	8					-0.632
Insula_L'	Efficiency	0.453	0.002	0.450	0.002	0.568	0.452	7.653	0.923	2					0.096
Insula_R'	Efficiency	0.397	0.002	0.399	0.002	1.174	0.280	7.653	0.741	8					-0.330
Cingulum_Ant_L'	Efficiency	0.370	0.002	0.378	0.002	6.919	0.009	7.653	0.048	10					-1.974
Cingulum_Ant_R'	Efficiency	0.424	0.002	0.427	0.002	3.173	0.076	7.653	0.287	4					-1.064
Cingulum_Mid_L'	Efficiency	0.370	0.002	0.377	0.002	9.000	0.003	7.653	0.022	12	1.663	2.019			-2.296
Cingulum_Mid_R'	Efficiency	0.468	0.002	0.466	0.002	0.434	0.511	7.653	0.967	2					0.042
Cingulum_Post_L'	Efficiency	0.379	0.003	0.383	0.002	1.854	0.175	7.653	0.536	12					-0.619
**Cingulum_Post_R'**	**Efficiency**	**0.355**	**0.002**	**0.364**	**0.002**	**9.391**	**0.002**	**7.653**	**0.017**	**12**	**5.569**	**4.126**	**-2.756**	**0.006**	**-2.393**
Hippocampus_L'	Efficiency	0.362	0.004	0.367	0.003	1.188	0.277	7.653	0.706	12					-0.377
Hippocampus_R'	Efficiency	0.413	0.003	0.420	0.003	3.649	0.057	7.653	0.204	6					-1.271
ParaHippocampal_L'	Efficiency	0.306	0.005	0.321	0.004	5.184	0.024	7.653	0.098	14					-1.652
ParaHippocampal_R'	Efficiency	0.366	0.003	0.374	0.003	4.093	0.044	7.653	0.180	6					-1.340
Amygdala_L'	Efficiency	0.276	0.004	0.286	0.003	4.497	0.035	7.653	0.192	12					-1.305
Amygdala_R'	Efficiency	0.230	0.006	0.245	0.003	6.552	0.011	7.653	0.047	18					-1.989
Calcarine_L'	Efficiency	0.446	0.003	0.451	0.003	1.777	0.184	7.653	0.503	4					-0.671
Calcarine_R'	Efficiency	0.388	0.003	0.399	0.003	8.574	0.004	7.653	0.018	8	2.367	2.963			-2.358
**Cuneus_L'**	**Efficiency**	**0.355**	**0.003**	**0.366**	**0.003**	**10.104**	**0.002**	**7.653**	**0.010**	**12**	**8.876**	**5.780**	**-2.867**	**0.005**	**-2.575**
Cuneus_R'	Efficiency	0.321	0.003	0.328	0.003	4.093	0.044	7.653	0.172	18					-1.366
Lingual_L'	Efficiency	0.461	0.003	0.467	0.003	3.874	0.050	7.653	0.175	4					-1.355
Lingual_R'	Efficiency	0.415	0.003	0.424	0.003	5.918	0.016	7.653	0.063	8					-1.856
Occipital_Sup_L'	Efficiency	0.448	0.002	0.457	0.002	8.154	0.005	7.653	0.025	4	0.497	6.251			-2.241
Occipital_Sup_R'	Efficiency	0.413	0.003	0.422	0.003	6.265	0.013	7.653	0.055	8					-1.918
**Occipital_Mid_L'**	**Efficiency**	**0.450**	**0.002**	**0.462**	**0.003**	**14.856**	**0.000**	**7.653**	**0.002**	**4**	**31.040**	**5.525**	**-3.511**	**0.001**	**-3.143**
Occipital_Mid_R'	Efficiency	0.413	0.002	0.419	0.002	3.907	0.049	7.653	0.205	4					-1.266
Occipital_Inf_L'	Efficiency	0.318	0.003	0.318	0.003	0.275	0.600	7.653	0.992	14					-0.010
Occipital_Inf_R'	Efficiency	0.306	0.002	0.312	0.003	3.247	0.073	7.653	0.284	14					-1.072
Fusiform_L'	Efficiency	0.362	0.003	0.372	0.003	5.521	0.020	7.653	0.065	8					-1.845
Fusiform_R'	Efficiency	0.332	0.003	0.336	0.003	1.841	0.176	7.653	0.519	14					-0.645
Postcentral_L'	Efficiency	0.413	0.002	0.418	0.002	1.952	0.164	7.653	0.508	6					-0.663
Postcentral_R'	Efficiency	0.434	0.002	0.440	0.002	3.707	0.055	7.653	0.215	6					-1.239
Parietal_Sup_L'	Efficiency	0.532	0.002	0.536	0.002	1.792	0.182	7.653	0.561	0					-0.581
**Parietal_Sup_R'**	**Efficiency**	**0.408**	**0.002**	**0.417**	**0.002**	**9.414**	**0.002**	**7.653**	**0.008**	**12**	**4.646**	**1.815**	**-2.803**	**0.005**	**-2.638**
Parietal_Inf_L'	Efficiency	0.338	0.002	0.343	0.002	2.253	0.135	7.653	0.417	12					-0.811
Parietal_Inf_R'	Efficiency	0.312	0.002	0.316	0.002	3.461	0.064	7.653	0.250	12					-1.149
SupraMarginal_L'	Efficiency	0.418	0.002	0.415	0.002	1.933	0.166	7.653	0.469	0					0.724
SupraMarginal_R'	Efficiency	0.327	0.002	0.329	0.002	1.086	0.298	7.653	0.775	12					-0.286

Angular_L'	Efficiency	0.308	0.002	0.312	0.002	2.229	0.137	7.653	0.426	14					-0.797
Angular_R'	Efficiency	0.342	0.002	0.349	0.002	4.298	0.039	7.653	0.145	12					-1.457
**Precuneus_L'**	**Efficiency**	**0.494**	**0.002**	**0.507**	**0.002**	**16.596**	**6.26471565**	**7.653**	**0.000**	**6**	**73.328**	**2.082**	**-3.876**	**0.000**	**-Inf**
Precuneus_R'	Efficiency	0.489	0.002	0.495	0.002	4.051	0.045	7.653	0.169	6					-1.377
Paracentral_Lobule_L'	Efficiency	0.417	0.002	0.423	0.002	5.500	0.020	7.653	0.090	4					-1.695
Paracentral_Lobule_R'	Efficiency	0.315	0.003	0.325	0.002	6.404	0.012	7.653	0.047	20					-1.989
Caudate_L'	Efficiency	0.349	0.003	0.358	0.003	6.544	0.011	7.653	0.065	10					-1.845
Caudate_R'	Efficiency	0.369	0.002	0.379	0.002	11.610	0.001	7.653	0.007	8	11.276	12.296			-2.712
Putamen_L'	Efficiency	0.444	0.003	0.451	0.003	3.859	0.051	7.653	0.155	8					-1.421
Putamen_R'	Efficiency	0.455	0.003	0.464	0.002	8.030	0.005	7.653	0.015	8	0.240	2.136			-2.432
Pallidum_L'	Efficiency	0.328	0.003	0.335	0.003	4.428	0.036	7.653	0.174	8					-1.361
Pallidum_R'	Efficiency	0.309	0.002	0.317	0.003	4.073	0.045	7.653	0.222	12					-1.221
Thalamus_L'	Efficiency	0.343	0.003	0.349	0.004	1.415	0.235	7.653	0.641	18					-0.466
Thalamus_R'	Efficiency	0.384	0.003	0.392	0.003	3.843	0.051	7.653	0.174	12					-1.361
Heschl_L'	Efficiency	0.202	0.009	0.226	0.008	3.961	0.048	7.653	0.269	12					-1.106
Heschl_R'	Efficiency	0.170	0.009	0.190	0.008	3.594	0.059	7.653	0.311	16					-1.014
Temporal_Sup_L'	Efficiency	0.320	0.003	0.325	0.003	3.079	0.081	7.653	0.270	16					-1.102
Temporal_Sup_R'	Efficiency	0.380	0.003	0.385	0.003	1.814	0.179	7.653	0.494	6					-0.684
**Temporal_Pole_Sup_I**	**Efficiency**	**0.325**	**0.005**	**0.341**	**0.003**	**8.964**	**0.003**	**7.653**	**0.010**	**10**	**7.584**	**5.048**	**-2.872**	**0.004**	**-2.575**
Temporal_Pole_Sup_R'	Efficiency	0.249	0.005	0.255	0.005	0.869	0.352	7.653	0.865	18					-0.170
Temporal_Mid_L'	Efficiency	0.370	0.003	0.376	0.002	2.550	0.112	7.653	0.349	12					-0.937
Temporal_Mid_R'	Efficiency	0.373	0.003	0.381	0.002	5.250	0.023	7.653	0.092	8					-1.686
Temporal_Pole_Mid_L'	Efficiency	0.307	0.003	0.314	0.003	2.870	0.092	7.653	0.346	16					-0.943
Temporal_Pole_Mid_R'	Efficiency	0.378	0.003	0.386	0.003	5.403	0.021	7.653	0.077	6					-1.770
Temporal_Inf_L'	Efficiency	0.509	0.003	0.501	0.003	4.585	0.033	7.653	0.164	0					1.393
Temporal_Inf_R'	Efficiency	0.349	0.003	0.353	0.003	1.835	0.177	7.653	0.497	12					-0.678
Cerebelum_Crus1_L'	Efficiency	0.353	0.006	0.368	0.005	4.667	0.032	7.653	0.120	6					-1.554
Cerebelum_Crus1_R'	Efficiency	0.364	0.005	0.379	0.004	7.055	0.008	7.653	0.050	4					-1.959
Cerebelum_Crus2_L'	Efficiency	0.248	0.007	0.264	0.006	4.893	0.028	7.653	0.139	12					-1.481
Cerebelum_Crus2_R'	Efficiency	0.432	0.003	0.442	0.003	4.699	0.031	7.653	0.155	0					-1.421
Cerebelum_3_L'	Efficiency	0.074	0.009	0.096	0.010	2.874	0.091	7.653	0.369	12					-0.898
Cerebelum_3_R'	Efficiency	0.018	0.005	0.032	0.006	3.942	0.048	7.653	0.237	20					-1.182
Cerebelum_4_5_L'	Efficiency	0.303	0.005	0.317	0.004	4.836	0.029	7.653	0.135	6					-1.494
Cerebelum_4_5_R'	Efficiency	0.401	0.005	0.412	0.003	3.896	0.050	7.653	0.220	0					-1.226
Cerebelum_6_L'	Efficiency	0.404	0.004	0.414	0.003	5.018	0.026	7.653	0.122	2					-1.547
Cerebelum_6_R'	Efficiency	0.302	0.007	0.318	0.005	5.170	0.024	7.653	0.112	8					-1.590
Cerebelum_7b_L'	Efficiency	0.287	0.005	0.302	0.004	7.198	0.008	7.653	0.040	6					-2.053
Cerebelum_7b_R'	Efficiency	0.394	0.003	0.403	0.003	4.078	0.045	7.653	0.220	0					-1.226
Cerebelum_8_L'	Efficiency	0.329	0.004	0.337	0.003	3.118	0.079	7.653	0.352	4					-0.930
Cerebelum_8_R'	Efficiency	0.352	0.004	0.365	0.003	6.696	0.010	7.653	0.047	4					-1.989
Cerebelum_9_L'	Efficiency	0.280	0.004	0.288	0.003	3.460	0.064	7.653	0.255	4					-1.137
Cerebelum_9_R'	Efficiency	0.362	0.003	0.374	0.003	7.642	0.006	7.653	0.037	0					-2.089
Cerebelum_10_L'	Efficiency	0.318	0.003	0.330	0.003	10.023	0.002	7.653	0.012	2	2.699	8.151			-2.521
Cerebelum_10_R'	Efficiency	0.229	0.005	0.242	0.004	5.789	0.017	7.653	0.110	8					-1.597
Vermis_1_2'	Efficiency	0.029	0.006	0.018	0.004	2.099	0.149	7.653	0.503	16					0.671
Vermis_3'	Efficiency	0.014	0.004	0.024	0.006	2.196	0.140	7.653	0.558	14					-0.586

**Vermis_4_5'**	**Efficiency**	**0.108**	**0.009**	**0.149**	**0.009**	**10.824**	**0.001**	**7.653**	**0.007**	**18**	**8.308**	**4.428**	**-3.315**	**0.001**	**-2.712**
Vermis_6'	Efficiency	0.345	0.008	0.366	0.005	6.370	0.012	7.653	0.067	0					-1.833
Vermis_7'	Efficiency	0.048	0.007	0.071	0.008	5.948	0.015	7.653	0.070	20					-1.811
Vermis_8'	Efficiency	0.204	0.008	0.222	0.007	3.231	0.074	7.653	0.391	8					-0.858
Vermis_9'	Efficiency	0.146	0.009	0.163	0.009	2.001	0.159	7.653	0.559	12					-0.584
Vermis_10'	Efficiency	0.189	0.008	0.209	0.009	3.600	0.059	7.653	0.285	8					-1.068
Precentral_L'	Mean Distan	2.685	0.017	2.660	0.014	0.696	0.407	8.732	0.866	2					0.168
Precentral_R'	Mean Distan	2.624	0.016	2.687	0.014	3.026	0.086	8.732	0.259	2					-1.129
Frontal_Sup_L'	Mean Distan	2.235	0.011	2.204	0.011	3.320	0.070	8.732	0.237	0					1.182
Frontal_Sup_R'	Mean Distan	2.759	0.026	2.631	0.010	1.844	0.212	8.732	0.476	6					0.713
Frontal_Sup_Orb_L'	Mean Distan	2.444	0.015	2.397	0.013	3.997	0.047	8.732	0.180	0					1.340
Frontal_Sup_Orb_R'	Mean Distan	2.608	0.014	2.702	0.019	6.048	0.016	8.732	0.068	2					-1.822
Frontal_Mid_L'	Mean Distan	2.832	0.018	2.928	0.016	1.601	0.215	8.732	0.534	4					-0.622
Frontal_Mid_R'	Mean Distan	2.681	0.016	2.763	0.014	5.462	0.022	8.732	0.095	2					-1.669
Frontal_Mid_Orb_L'	Mean Distan	2.879	0.020	2.823	0.017	1.398	0.240	8.732	0.606	2					0.516
Frontal_Mid_Orb_R'	Mean Distan	3.200	0.029	3.085	0.017	2.285	0.169	8.732	0.374	6					0.889
Frontal_Inf_Oper_L'	Mean Distan	2.901	0.013	3.024	0.011	6.224	0.018	8.732	0.077	4					-1.770
Frontal_Inf_Oper_R'	Mean Distan	3.394	0.038	3.181	0.011	5.677	0.044	8.732	0.077	6					1.770
Frontal_Inf_Tri_L'	Mean Distan	2.868	0.016	2.964	0.015	2.993	0.094	8.732	0.275	4					-1.091
Frontal_Inf_Tri_R'	Mean Distan	3.183	0.037	2.960	0.011	5.380	0.049	8.732	0.097	6					1.660
Frontal_Inf_Orb_L'	Mean Distan	2.850	0.017	2.742	0.016	7.933	0.006	8.732	0.042	2					2.036
Frontal_Inf_Orb_R'	Mean Distan	3.099	0.043	3.094	0.014	0.672	0.436	8.732	0.865	6					0.170
Rolandic_Oper_L'	Mean Distan	2.958	0.014	2.917	0.015	1.888	0.173	8.732	0.529	2					0.629
Rolandic_Oper_R'	Mean Distan	3.302	0.046	3.122	0.008	4.158	0.076	8.732	0.174	6					1.361
Supp_Motor_Area_L'	Mean Distan	2.295	0.010	2.269	0.010	2.403	0.123	8.732	0.369	0					0.898
Supp_Motor_Area_R'	Mean Distan	3.078	0.028	2.819	0.010	5.128	0.053	8.732	0.098	6					1.652
Olfactory_L'	Mean Distan	3.163	0.017	3.233	0.020	2.472	0.120	8.732	0.367	2					-0.902
Olfactory_R'	Mean Distan	3.991	0.044	3.834	0.023	2.756	0.136	8.732	0.324	6					0.987
Frontal_Sup_Medial_L'	Mean Distan	2.229	0.010	2.192	0.010	4.627	0.033	8.732	0.170	0					1.371
Frontal_Sup_Medial_R'	Mean Distan	2.715	0.015	2.611	0.014	5.824	0.022	8.732	0.082	4					1.740
Frontal_Med_Orb_L'	Mean Distan	3.233	0.021	3.104	0.010	2.311	0.167	8.732	0.429	6					0.791
Frontal_Med_Orb_R'	Mean Distan	3.397	0.026	3.266	0.011	3.968	0.082	8.732	0.192	6					1.305
Rectus_L'	Mean Distan	3.433	0.023	3.269	0.019	2.126	0.183	8.732	0.479	6					0.708
Rectus_R'	Mean Distan	3.524	0.027	3.477	0.017	3.229	0.110	8.732	0.277	6					1.087
Insula_L'	Mean Distan	2.621	0.018	2.652	0.013	0.563	0.455	8.732	0.881	2					-0.149
Insula_R'	Mean Distan	2.267	0.009	2.286	0.010	1.349	0.247	8.732	0.648	0					-0.457
Cingulum_Ant_L'	Mean Distan	3.114	0.010	2.861	0.012	6.961	0.030	8.732	0.060	6					1.880
Cingulum_Ant_R'	Mean Distan	2.938	0.021	2.868	0.006	4.085	0.078	8.732	0.197	6					1.290
Cingulum_Mid_L'	Mean Distan	2.914	0.008	2.766	0.012	1.192	0.307	8.732	0.721	6					0.357
Cingulum_Mid_R'	Mean Distan	2.640	0.018	2.561	0.010	3.295	0.079	8.732	0.295	4					1.046
Cingulum_Post_L'	Mean Distan	2.780	0.016	2.693	0.010	0.755	0.410	8.732	0.873	6					0.160
Cingulum_Post_R'	Mean Distan	2.914	0.021	2.778	0.012	4.328	0.071	8.732	0.170	6					1.371
Hippocampus_L'	Mean Distan	2.413	0.013	2.393	0.014	0.564	0.455	8.732	0.905	2					0.120
Hippocampus_R'	Mean Distan	2.933	0.027	2.693	0.016	5.514	0.047	8.732	0.083	6					1.731
ParaHippocampal_L'	Mean Distan	2.694	0.020	2.621	0.017	3.625	0.060	8.732	0.217	2					1.234
ParaHippocampal_R'	Mean Distan	3.237	0.052	3.015	0.016	2.662	0.141	8.732	0.336	6					0.963

Amygdala_L'	Mean Distan	3.176	0.025	3.295	0.034	1.058	0.312	8.732	0.721	4	-0.357
Amygdala_R'	Mean Distan	3.750	0.045	3.496	0.023	1.624	0.238	8.732	0.558	6	0.586
Calcarine_L'	Mean Distan	2.393	0.014	2.339	0.010	3.697	0.058	8.732	0.210	2	1.253
Calcarine_R'	Mean Distan	2.472	0.014	2.427	0.014	2.579	0.112	8.732	0.381	2	0.877
Cuneus_L'	Mean Distan	2.551	0.017	2.455	0.013	7.628	0.007	8.732	0.050	2	1.959
Cuneus_R'	Mean Distan	2.804	0.017	2.640	0.014	8.341	0.007	8.732	0.023	4	2.267
Lingual_L'	Mean Distan	2.313	0.018	2.281	0.011	1.215	0.273	8.732	0.689	2	0.400
Lingual_R'	Mean Distan	2.776	0.043	2.617	0.017	3.231	0.110	8.732	0.250	6	1.149
Occipital_Sup_L'	Mean Distan	2.199	0.009	2.163	0.009	6.311	0.013	8.732	0.068	0	1.822
Occipital_Sup_R'	Mean Distan	2.402	0.013	2.356	0.010	3.658	0.059	8.732	0.225	2	1.212
Occipital_Mid_L'	Mean Distan	2.194	0.011	2.163	0.012	3.714	0.056	8.732	0.215	0	1.239
Occipital_Mid_R'	Mean Distan	2.622	0.016	2.575	0.011	2.589	0.111	8.732	0.369	2	0.898
Occipital_Inf_L'	Mean Distan	2.853	0.020	3.006	0.017	3.499	0.071	8.732	0.240	4	-1.174
Occipital_Inf_R'	Mean Distan	3.175	0.031	3.092	0.021	1.348	0.279	8.732	0.666	6	0.431
Fusiform_L'	Mean Distan	2.751	0.022	2.886	0.020	2.186	0.149	8.732	0.454	4	-0.749
Fusiform_R'	Mean Distan	3.091	0.054	2.866	0.022	4.103	0.077	8.732	0.190	6	1.310
Postcentral_L'	Mean Distan	2.955	0.032	2.840	0.014	0.696	0.428	8.732	0.876	6	0.155
Postcentral_R'	Mean Distan	2.612	0.014	2.541	0.016	1.438	0.240	8.732	0.588	4	0.542
Parietal_Sup_L'	Mean Distan	2.145	0.011	2.120	0.009	2.167	0.143	8.732	0.456	0	0.746
Parietal_Sup_R'	Mean Distan	2.472	0.013	2.449	0.010	1.194	0.283	8.732	0.693	4	0.395
Parietal_Inf_L'	Mean Distan	2.571	0.013	2.544	0.013	1.669	0.198	8.732	0.566	0	0.574
Parietal_Inf_R'	Mean Distan	3.153	0.011	3.115	0.017	0.913	0.347	8.732	0.795	4	0.260
SupraMarginal_L'	Mean Distan	2.719	0.014	2.752	0.015	2.352	0.127	8.732	0.406	0	-0.832
SupraMarginal_R'	Mean Distan	3.364	0.042	3.122	0.014	4.006	0.080	8.732	0.170	6	1.371
Angular_L'	Mean Distan	3.020	0.017	3.158	0.015	4.572	0.040	8.732	0.149	4	-1.445
Angular_R'	Mean Distan	3.194	0.016	3.008	0.015	2.979	0.123	8.732	0.301	6	1.035
Precuneus_L'	Mean Distan	2.160	0.012	2.092	0.009	8.435	0.005	8.732	0.038	2	2.071
Precuneus_R'	Mean Distan	2.312	0.017	2.248	0.008	1.908	0.177	8.732	0.477	4	0.710
Paracentral_Lobule_L'	Mean Distan	2.347	0.012	2.298	0.011	5.827	0.017	8.732	0.102	0	1.636
Paracentral_Lobule_R'	Mean Distan	3.024	0.026	2.779	0.016	3.123	0.115	8.732	0.235	6	1.187
Caudate_L'	Mean Distan	2.386	0.014	2.348	0.014	2.776	0.097	8.732	0.326	0	0.983
Caudate_R'	Mean Distan	2.960	0.020	2.829	0.013	4.695	0.038	8.732	0.104	4	1.628
Putamen_L'	Mean Distan	2.335	0.015	2.302	0.012	1.171	0.282	8.732	0.716	2	0.364
Putamen_R'	Mean Distan	2.469	0.021	2.365	0.010	5.139	0.031	8.732	0.105	4	1.620
Pallidum_L'	Mean Distan	3.069	0.029	3.011	0.020	0.704	0.408	8.732	0.841	4	0.200
Pallidum_R'	Mean Distan	3.062	0.022	2.816	0.020	10.262	0.003	8.732	0.025	4	2.241
Thalamus_L'	Mean Distan	2.394	0.019	2.337	0.016	2.233	0.139	8.732	0.424	2	0.799
Thalamus_R'	Mean Distan	2.599	0.021	2.483	0.016	2.863	0.101	8.732	0.302	4	1.032
Heschl_L'	Mean Distan	3.489	0.025	3.613	0.030	1.091	0.304	8.732	0.706	4	-0.377
Heschl_R'	Mean Distan	3.047	0.022	3.127	0.019	4.815	0.030	8.732	0.129	0	-1.520
Temporal_Sup_L'	Mean Distan	2.894	0.014	2.963	0.020	1.207	0.280	8.732	0.646	4	-0.459
Temporal_Sup_R'	Mean Distan	3.272	0.040	3.116	0.020	3.694	0.091	8.732	0.205	6	1.266
Temporal_Pole_Sup_L'	Mean Distan	2.463	0.014	2.409	0.010	7.795	0.006	8.732	0.033	0	2.127
Temporal_Pole_Sup_R'	Mean Distan	3.601	0.045	3.330	0.031	6.344	0.036	8.732	0.057	6	1.905
Temporal_Mid_L'	Mean Distan	2.766	0.019	2.895	0.019	3.009	0.093	8.732	0.282	4	-1.076
Temporal_Mid_R'	Mean Distan	3.192	0.039	3.034	0.017	2.876	0.128	8.732	0.290	6	1.057

Temporal_Pole_Mid_L'	Mean Distan	2.679	0.016	2.651	0.011	1.087	0.300	8.732	0.720	2					0.359
Temporal_Pole_Mid_R'	Mean Distan	3.237	0.034	2.959	0.022	8.494	0.019	8.732	0.028	6					2.192
Temporal_Inf_L'	Mean Distan	2.672	0.017	2.886	0.016	10.969	0.002	8.732	0.010	4					-2.575
Temporal_Inf_R'	Mean Distan	2.282	0.014	2.316	0.016	1.885	0.172	8.732	0.504	0					-0.668
Cerebelum_Crus1_L'	Mean Distan	2.886	0.023	2.959	0.033	0.471	0.498	8.732	0.927	4					-0.092
Cerebelum_Crus1_R'	Mean Distan	3.623	0.071	3.130	0.033	11.077	0.010	8.732	0.010	6					2.575
Cerebelum_Crus2_L'	Mean Distan	2.656	0.024	2.692	0.022	0.712	0.400	8.732	0.863	0					-0.172
Cerebelum_Crus2_R'	Mean Distan	2.678	0.022	2.611	0.020	3.019	0.084	8.732	0.295	0					1.046
Cerebelum_3_L'	Mean Distan	2.971	0.031	2.897	0.032	2.100	0.149	8.732	0.469	0					0.724
Cerebelum_3_R'	Mean Distan	3.991	0.064	3.604	0.036	3.417	0.074	8.732	0.262	4					1.121
Cerebelum_4_5_L'	Mean Distan	3.208	0.027	3.343	0.024	2.699	0.111	8.732	0.416	4					-0.814
Cerebelum_4_5_R'	Mean Distan	2.763	0.022	2.703	0.024	2.383	0.124	8.732	0.427	0					0.794
Cerebelum_6_L'	Mean Distan	2.825	0.036	2.709	0.018	3.781	0.055	8.732	0.197	2					1.290
Cerebelum_6_R'	Mean Distan	3.073	0.052	3.001	0.030	0.607	0.442	8.732	0.878	4					0.153
Cerebelum_7b_L'	Mean Distan	3.249	0.040	3.187	0.024	1.551	0.216	8.732	0.594	2					0.533
Cerebelum_7b_R'	Mean Distan	2.872	0.029	2.818	0.022	1.606	0.207	8.732	0.604	0					0.518
Cerebelum_8_L'	Mean Distan	3.953	0.095	3.709	0.051	0.191	0.673	8.732	0.978	6					0.027
Cerebelum_8_R'	Mean Distan	2.605	0.023	2.542	0.021	3.177	0.076	8.732	0.265	0					1.114
Cerebelum_9_L'	Mean Distan	4.664	0.113	4.043	0.040	1.785	0.218	8.732	0.538	6					0.617
Cerebelum_9_R'	Mean Distan	3.117	0.026	3.048	0.030	2.596	0.109	8.732	0.379	0					0.880
Cerebelum_10_L'	Mean Distan	4.759	0.098	4.082	0.042	3.966	0.082	8.732	0.202	6					1.276
Cerebelum_10_R'	Mean Distan	3.277	0.029	3.192	0.028	3.342	0.069	8.732	0.250	0					1.149
Vermis_1_2'	Mean Distan	5.183	0.096	4.653	0.048	1.551	0.248	8.732	0.604	6					0.518
Vermis_3'	Mean Distan	3.450	0.037	3.388	0.033	1.658	0.200	8.732	0.578	0					0.557
Vermis_4_5'	Mean Distan	2.713	0.021	2.650	0.022	2.582	0.110	8.732	0.374	0					0.889
Vermis_6'	Mean Distan	3.727	0.059	3.542	0.032	2.366	0.128	8.732	0.457	2					0.743
Vermis_7'	Mean Distan	4.012	0.054	3.744	0.047	1.737	0.197	8.732	0.568	4					0.572
Vermis_8'	Mean Distan	2.959	0.028	2.885	0.026	2.410	0.122	8.732	0.437	0					0.777
Vermis_9'	Mean Distan	2.983	0.033	2.943	0.037	0.637	0.426	8.732	0.851	0					0.187
Vermis_10'	Mean Distan	5.151	0.068	4.362	0.085	1.016	0.343	8.732	0.750	6					0.319
Precentral_L'	Betweennes	126.685	8.726	105.634	7.413	3.049	0.082	7.825	0.312	4					1.011
Precentral_R'	Betweennes	96.072	6.856	80.950	6.243	2.423	0.121	7.825	0.536	12					0.619
Frontal_Sup_L'	Betweennes	322.345	12.062	377.509	20.532	5.673	0.018	7.825	0.119	2					-1.561
Frontal_Sup_R'	Betweennes	730.202	29.701	681.622	19.785	1.914	0.168	7.825	0.561	20					0.581
Frontal_Sup_Orb_L'	Betweennes	149.103	11.572	169.857	13.143	1.484	0.224	7.825	0.669	4					-0.427
**Frontal_Sup_Orb_R'**	**Betweennes**	**359.623**	**20.011**	**270.877**	**15.845**	**10.939**	**0.001**	**7.825**	**0.005**	**10**	**10.604**	**9.306**	**3.483**	**0.001**	**2.806**
Frontal_Mid_L'	Betweennes	137.884	10.511	165.688	19.570	1.019	0.314	7.825	0.743	10					-0.328
Frontal_Mid_R'	Betweennes	124.807	12.642	97.931	7.972	2.939	0.088	7.825	0.322	14					0.990
Frontal_Mid_Orb_L'	Betweennes	33.692	4.451	40.093	5.780	0.824	0.365	7.825	0.937	14					-0.080
Frontal_Mid_Orb_R'	Betweennes	50.832	6.911	36.123	4.048	3.886	0.050	7.825	0.254	14					1.141
Frontal_Inf_Oper_L'	Betweennes	114.978	8.640	99.450	5.546	2.183	0.141	7.825	0.508	10					0.663
Frontal_Inf_Oper_R'	Betweennes	46.152	3.380	57.954	4.099	5.701	0.018	7.825	0.090	6					-1.695
Frontal_Inf_Tri_L'	Betweennes	71.408	5.232	78.637	6.264	0.842	0.360	7.825	0.835	2					-0.209
Frontal_Inf_Tri_R'	Betweennes	71.454	6.279	60.483	3.726	1.960	0.163	7.825	0.524	4					0.637
Frontal_Inf_Orb_L'	Betweennes	64.961	7.016	89.305	9.272	4.439	0.036	7.825	0.149	0					-1.445
Frontal_Inf_Orb_R'	Betweennes	77.716	9.696	106.798	12.233	3.526	0.062	7.825	0.282	10					-1.076

Rolandic_Oper_L'	Betweennes	33.936	2.033	39.746	2.320	3.603	0.059	7.825	0.257	0			-1.133
Rolandic_Oper_R'	Betweennes	40.813	2.808	48.077	2.932	3.555	0.061	7.825	0.290	0			-1.057
Supp_Motor_Area_L'	Betweennes	157.210	7.071	172.777	9.231	1.965	0.162	7.825	0.551	0			-0.596
Supp_Motor_Area_R'	Betweennes	244.871	15.942	193.330	14.154	5.057	0.025	7.825	0.112	14			1.590
Olfactory_L'	Betweennes	53.928	6.369	35.389	4.941	5.650	0.018	7.825	0.097	16			1.660
Olfactory_R'	Betweennes	13.657	1.668	8.008	1.039	7.628	0.006	7.825	0.042	6			2.036
Frontal_Sup_Medial_L'	Betweennes	514.979	23.636	457.285	20.584	2.942	0.088	7.825	0.341	10			0.953
Frontal_Sup_Medial_R'	Betweennes	213.814	10.785	261.953	13.966	6.344	0.012	7.825	0.052	8			-1.945
Frontal_Med_Orb_L'	Betweennes	55.184	3.979	50.066	3.890	0.832	0.363	7.825	0.876	0			0.155
Frontal_Med_Orb_R'	Betweennes	70.648	5.063	88.146	6.726	4.387	0.037	7.825	0.220	4			-1.226
Rectus_L'	Betweennes	71.824	5.594	78.394	5.375	0.931	0.335	7.825	0.928	2			-0.090
Rectus_R'	Betweennes	81.306	8.460	93.852	8.684	1.007	0.317	7.825	0.868	18			-0.166
Insula_L'	Betweennes	211.850	17.723	152.986	11.007	8.519	0.004	7.825	0.028	10	0.311	6.029	2.192
Insula_R'	Betweennes	300.427	16.230	282.118	14.213	0.878	0.350	7.825	0.861	2			0.175
Cingulum_Ant_L'	Betweennes	239.529	12.236	268.216	14.381	2.812	0.095	7.825	0.442	14			-0.768
Cingulum_Ant_R'	Betweennes	138.888	6.088	151.697	6.656	2.115	0.147	7.825	0.551	0			-0.596
Cingulum_Mid_L'	Betweennes	70.706	5.309	55.837	3.756	6.490	0.011	7.825	0.053	4			1.931
Cingulum_Mid_R'	Betweennes	169.791	11.176	134.204	7.906	7.774	0.006	7.825	0.023	8			2.267
Cingulum_Post_L'	Betweennes	236.826	23.969	199.820	16.660	1.967	0.162	7.825	0.631	8			0.480
Cingulum_Post_R'	Betweennes	86.690	7.973	125.360	23.419	2.174	0.142	7.825	0.563	14			-0.579
Hippocampus_L'	Betweennes	337.840	30.181	275.666	25.921	2.086	0.150	7.825	0.546	20			0.604
Hippocampus_R'	Betweennes	259.772	20.570	233.650	12.363	1.825	0.178	7.825	0.639	2			0.469
ParaHippocampal_L'	Betweennes	102.278	17.763	68.147	6.705	3.646	0.057	7.825	0.180	2			1.340
ParaHippocampal_R'	Betweennes	109.612	11.179	122.193	10.004	0.499	0.481	7.825	0.983	18			-0.021
Amygdala_L'	Betweennes	21.353	3.261	30.807	5.984	2.508	0.115	7.825	0.533	6			-0.624
Amygdala_R'	Betweennes	23.461	4.426	13.953	2.961	3.556	0.061	7.825	0.306	14			1.025
Calcarine_L'	Betweennes	286.345	20.776	314.819	22.460	0.706	0.402	7.825	0.953	0			-0.059
Calcarine_R'	Betweennes	166.116	11.353	203.386	16.417	2.953	0.087	7.825	0.406	0			-0.832
Cuneus_L'	Betweennes	84.019	10.432	152.103	18.875	9.485	0.002	7.825	0.008	18	0.852	4.460	-2.638
Cuneus_R'	Betweennes	94.475	9.370	121.859	13.314	2.734	0.100	7.825	0.481	4			-0.705
Lingual_L'	Betweennes	1028.456	67.335	1154.760	75.199	1.366	0.244	7.825	0.800	4			-0.254
Lingual_R'	Betweennes	1071.965	91.059	1321.214	107.519	3.407	0.066	7.825	0.347	10			-0.940
Occipital_Sup_L'	Betweennes	240.557	18.866	274.803	25.355	1.218	0.271	7.825	0.771	4			-0.291
Occipital_Sup_R'	Betweennes	344.931	29.038	399.989	33.092	1.335	0.249	7.825	0.730	16			-0.346
Occipital_Mid_L'	Betweennes	241.504	16.272	292.688	18.777	5.326	0.022	7.825	0.110	0			-1.597
Occipital_Mid_R'	Betweennes	205.963	23.973	167.004	14.477	2.171	0.142	7.825	0.481	20			0.705
Occipital_Inf_L'	Betweennes	88.738	9.538	67.672	7.601	3.329	0.069	7.825	0.341	0			0.953
Occipital_Inf_R'	Betweennes	80.219	14.474	41.668	8.014	4.599	0.033	7.825	0.150	4			1.439
Fusiform_L'	Betweennes	239.905	44.914	446.775	69.846	6.871	0.009	7.825	0.053	12			-1.931
Fusiform_R'	Betweennes	378.618	27.825	327.365	22.307	2.028	0.156	7.825	0.633	0			0.478
Postcentral_L'	Betweennes	228.115	14.898	250.953	20.064	0.638	0.425	7.825	0.945	6			-0.069
Postcentral_R'	Betweennes	346.756	17.597	325.313	15.258	0.631	0.428	7.825	0.955	4			0.057
Parietal_Sup_L'	Betweennes	795.334	41.328	693.765	37.393	3.428	0.065	7.825	0.295	20			1.046
Parietal_Sup_R'	Betweennes	644.012	35.959	698.634	36.711	1.341	0.248	7.825	0.751	14			-0.317
Parietal_Inf_L'	Betweennes	43.404	4.874	35.084	3.480	2.299	0.131	7.825	0.461	4			0.738
Parietal_Inf_R'	Betweennes	11.272	1.075	14.465	1.660	2.968	0.086	7.825	0.396	10			-0.849

SupraMarginal_L'	Betweennes	23.697	3.181	17.095	2.100	4.106	0.044	7.825	0.202	4					1.276
SupraMarginal_R'	Betweennes	25.371	2.178	20.861	2.132	2.691	0.102	7.825	0.432	2					0.785
Angular_L'	Betweennes	16.054	1.752	19.927	2.861	2.018	0.157	7.825	0.519	12					-0.645
Angular_R'	Betweennes	87.726	6.519	107.019	10.440	2.502	0.115	7.825	0.396	12					-0.849
Precuneus_L'	Betweennes	989.860	38.666	1108.418	38.734	4.424	0.036	7.825	0.200	4					-1.281
Precuneus_R'	Betweennes	846.059	38.958	762.631	35.542	2.410	0.122	7.825	0.513	2					0.655
Paracentral_Lobule_L'	Betweennes	77.208	6.711	89.099	8.079	1.125	0.290	7.825	0.881	0					-0.149
Paracentral_Lobule_R'	Betweennes	67.535	6.755	81.352	7.261	1.684	0.196	7.825	0.751	20					-0.317
Caudate_L'	Betweennes	108.028	11.263	139.704	13.297	2.877	0.091	7.825	0.311	10					-1.014
**Caudate_R'**	**Betweennes**	**83.233**	**8.242**	**144.370**	**14.234**	**13.275**	**0.000**	**7.825**	**0.000**	**8**	**24.475**	**8.858**	**-3.702**	**0.000**	**-Inf**
Putamen_L'	Betweennes	768.128	34.532	815.609	42.245	0.583	0.446	7.825	0.937	8					-0.080
Putamen_R'	Betweennes	903.971	39.041	989.886	41.991	2.252	0.135	7.825	0.479	8					-0.708
Pallidum_L'	Betweennes	60.025	7.037	46.440	4.640	2.302	0.131	7.825	0.528	0					0.632
Pallidum_R'	Betweennes	17.565	4.405	11.637	3.239	1.690	0.195	7.825	0.683	20					0.409
Thalamus_L'	Betweennes	489.540	35.368	566.429	40.864	1.797	0.181	7.825	0.579	2					-0.554
Thalamus_R'	Betweennes	410.521	51.759	525.408	69.202	2.164	0.143	7.825	0.553	12					-0.594
Heschl_L'	Betweennes	0.000	0.000	0.285	0.137	4.425	0.036	7.825	0.155	16					-1.421
Heschl_R'	Betweennes	0.398	0.100	0.191	0.046	3.120	0.079	7.825	0.207	0					1.262
Temporal_Sup_L'	Betweennes	203.563	13.804	237.235	17.176	3.439	0.065	7.825	0.377	16					-0.883
Temporal_Sup_R'	Betweennes	252.065	12.690	270.444	12.666	1.543	0.215	7.825	0.691	16					-0.397
Temporal_Pole_Sup_L'	Betweennes	59.644	6.676	85.958	10.722	4.645	0.032	7.825	0.189	18					-1.315
Temporal_Pole_Sup_R'	Betweennes	45.133	6.062	34.996	3.528	2.228	0.137	7.825	0.556	12					0.589
Temporal_Mid_L'	Betweennes	185.561	12.099	204.889	14.409	1.656	0.199	7.825	0.648	0					-0.457
Temporal_Mid_R'	Betweennes	144.806	14.787	125.513	8.182	1.165	0.282	7.825	0.811	0					0.239
Temporal_Pole_Mid_L'	Betweennes	184.138	14.433	145.728	13.284	4.012	0.046	7.825	0.235	14					1.187
Temporal_Pole_Mid_R'	Betweennes	94.846	8.149	72.537	5.423	4.787	0.030	7.825	0.145	0					1.457
**Temporal_Inf_L'**	**Betweennes**	**314.046**	**19.667**	**229.371**	**17.269**	**12.360**	**0.001**	**7.825**	**0.000**	**0**	**8.771**	**2.102**	**3.238**	**0.001**	**Inf**
Temporal_Inf_R'	Betweennes	194.243	18.310	152.759	12.762	2.821	0.094	7.825	0.396	20					0.849
Cerebelum_Crus1_L'	Betweennes	1146.052	84.589	1270.830	81.212	1.183	0.278	7.825	0.841	6					-0.200
Cerebelum_Crus1_R'	Betweennes	620.200	48.108	823.819	74.207	6.016	0.015	7.825	0.117	6					-1.568
Cerebelum_Crus2_L'	Betweennes	388.794	54.297	252.079	21.972	4.331	0.038	7.825	0.200	8					1.281
Cerebelum_Crus2_R'	Betweennes	552.656	49.810	373.517	39.908	7.658	0.006	7.825	0.047	10					1.989
Cerebelum_3_L'	Betweennes	42.493	10.519	86.825	21.039	3.222	0.074	7.825	0.234	2					-1.191
Cerebelum_3_R'	Betweennes	22.048	12.571	10.356	3.257	1.509	0.220	7.825	0.826	4					0.219
Cerebelum_4_5_L'	Betweennes	372.977	50.544	289.716	39.778	2.858	0.092	7.825	0.436	8					0.779
Cerebelum_4_5_R'	Betweennes	125.576	21.321	80.444	10.212	3.456	0.064	7.825	0.356	4					0.924
Cerebelum_6_L'	Betweennes	719.810	80.855	585.952	65.080	2.200	0.139	7.825	0.554	6					0.591
Cerebelum_6_R'	Betweennes	481.759	30.414	433.597	29.034	1.051	0.306	7.825	0.883	0					0.147
Cerebelum_7b_L'	Betweennes	82.794	12.950	56.006	7.336	3.294	0.071	7.825	0.382	0					0.874
Cerebelum_7b_R'	Betweennes	71.848	15.144	36.891	8.685	3.369	0.068	7.825	0.402	16					0.837
Cerebelum_8_L'	Betweennes	459.365	47.306	308.244	29.117	6.394	0.012	7.825	0.087	16					1.712
Cerebelum_8_R'	Betweennes	709.117	47.018	826.765	61.496	2.566	0.110	7.825	0.509	12					-0.660
Cerebelum_9_L'	Betweennes	49.919	10.877	29.389	4.866	3.640	0.058	7.825	0.294	0					1.050
Cerebelum_9_R'	Betweennes	38.074	8.562	24.446	4.310	1.687	0.195	7.825	0.818	2					0.230
Cerebelum_10_L'	Betweennes	23.132	2.907	43.083	6.312	9.286	0.003	7.825	0.017	2	0.511	4.146			-2.393
Cerebelum_10_R'	Betweennes	33.147	14.186	17.740	6.284	1.188	0.277	7.825	0.896	20					0.130

Vermis_1_2'	Betweennes	12.555	5.527	4.247	2.528	2.051	0.153	7.825	0.723	12				0.355
Vermis_3'	Betweennes	0.139	0.097	4.311	2.115	4.466	0.036	7.825	0.062	8				-1.868
Vermis_4_5'	Betweennes	282.432	30.721	385.337	41.821	2.969	0.086	7.825	0.466	10				-0.729
Vermis_6'	Betweennes	0.609	0.609	7.572	4.115	2.593	0.109	7.825	0.564	16				-0.577
Vermis_7'	Betweennes	30.730	8.512	64.747	20.349	2.867	0.092	7.825	0.422	14				-0.802
Vermis_8'	Betweennes	96.542	15.219	139.669	18.253	3.067	0.081	7.825	0.436	10				-0.779
Vermis_9'	Betweennes	202.801	58.893	59.113	15.443	5.417	0.021	7.825	0.109	14				1.605
Vermis_10'	Betweennes	84.067	20.185	140.326	40.097	1.870	0.173	7.825	0.728	10				-0.348
Precentral_L'	Clustering C	0.422	0.007	0.443	0.007	3.975	0.047	8.293	0.222	6				-1.221
Precentral_R'	Clustering C	0.422	0.010	0.439	0.010	1.216	0.271	8.293	0.786	16				-0.271
Frontal_Sup_L'	Clustering C	0.385	0.005	0.379	0.004	1.184	0.278	8.293	0.771	2				0.291
Frontal_Sup_R'	Clustering C	0.339	0.004	0.348	0.003	3.124	0.078	8.293	0.307	4				-1.021
Frontal_Sup_Orb_L'	Clustering C	0.427	0.011	0.438	0.010	0.581	0.447	8.293	0.958	6				-0.052
Frontal_Sup_Orb_R'	Clustering C	0.340	0.009	0.365	0.009	4.206	0.041	8.293	0.215	8				-1.239
Frontal_Mid_L'	Clustering C	0.459	0.012	0.429	0.010	3.266	0.072	8.293	0.287	14				1.064
Frontal_Mid_R'	Clustering C	0.505	0.009	0.526	0.010	1.741	0.188	8.293	0.563	4				-0.579
Frontal_Mid_Orb_L'	Clustering C	0.605	0.013	0.657	0.012	10.134	0.002	8.293	0.012	4	1.528	6.394			-2.521
Frontal_Mid_Orb_R'	Clustering C	0.623	0.013	0.664	0.013	5.612	0.019	8.293	0.090	6				-1.695
Frontal_Inf_Oper_L'	Clustering C	0.440	0.009	0.434	0.008	0.390	0.533	8.293	0.988	16				0.015
Frontal_Inf_Oper_R'	Clustering C	0.569	0.007	0.548	0.008	3.442	0.065	8.293	0.295	2				1.046
Frontal_Inf_Tri_L'	Clustering C	0.411	0.012	0.381	0.010	3.509	0.062	8.293	0.262	18				1.121
Frontal_Inf_Tri_R'	Clustering C	0.451	0.008	0.480	0.010	4.658	0.032	8.293	0.139	16				-1.481
Frontal_Inf_Orb_L'	Clustering C	0.501	0.008	0.487	0.007	1.539	0.216	8.293	0.735	0				0.339
Frontal_Inf_Orb_R'	Clustering C	0.545	0.018	0.488	0.016	7.013	0.009	8.293	0.073	18				1.790
Rolandic_Oper_L'	Clustering C	0.610	0.007	0.595	0.007	3.295	0.071	8.293	0.359	0				0.917
Rolandic_Oper_R'	Clustering C	0.542	0.008	0.522	0.007	3.391	0.067	8.293	0.302	2				1.032
Supp_Motor_Area_L'	Clustering C	0.335	0.007	0.353	0.009	2.967	0.086	8.293	0.351	20				-0.933
Supp_Motor_Area_R'	Clustering C	0.362	0.008	0.393	0.009	5.644	0.018	8.293	0.114	14				-1.583
Olfactory_L'	Clustering C	0.485	0.018	0.531	0.022	2.742	0.099	8.293	0.497	6				-0.678
Olfactory_R'	Clustering C	0.559	0.018	0.638	0.018	6.985	0.009	8.293	0.067	2				-1.833
Frontal_Sup_Medial_L'	Clustering C	0.342	0.005	0.353	0.005	1.266	0.262	8.293	0.753	8				-0.315
Frontal_Sup_Medial_R'	Clustering C	0.457	0.006	0.444	0.005	3.016	0.084	8.293	0.311	0				1.014
Frontal_Med_Orb_L'	Clustering C	0.379	0.017	0.419	0.017	3.232	0.073	8.293	0.356	14				-0.924
Frontal_Med_Orb_R'	Clustering C	0.429	0.017	0.386	0.016	4.158	0.043	8.293	0.259	14				1.129
Rectus_L'	Clustering C	0.447	0.011	0.433	0.010	1.072	0.301	8.293	0.907	6				0.117
Rectus_R'	Clustering C	0.307	0.030	0.280	0.027	1.118	0.291	8.293	0.895	18				0.132
Insula_L'	Clustering C	0.381	0.005	0.398	0.005	5.273	0.023	8.293	0.124	2				-1.540
Insula_R'	Clustering C	0.322	0.005	0.338	0.006	3.815	0.052	8.293	0.235	8				-1.187
Cingulum_Ant_L'	Clustering C	0.328	0.010	0.350	0.009	3.092	0.080	8.293	0.384	20				-0.871
Cingulum_Ant_R'	Clustering C	0.336	0.009	0.371	0.013	4.822	0.029	8.293	0.159	18				-1.410
Cingulum_Mid_L'	Clustering C	0.534	0.007	0.560	0.007	7.910	0.005	8.293	0.025	4				-2.241
**Cingulum_Mid_R'**	**Clustering [illegible]**	**0.466**	**0.005**	**0.494**	**0.005**	**16.153**	**7.79837812[illegible]**	**8.293**	**0.000**	**2**	**45.384**	**6.009**	**-3.916**	**0.000**	**-Inf**
Cingulum_Post_L'	Clustering C	0.380	0.006	0.405	0.008	5.747	0.017	8.293	0.088	6				-1.703
Cingulum_Post_R'	Clustering C	0.442	0.008	0.460	0.009	1.941	0.165	8.293	0.636	4				-0.473
Hippocampus_L'	Clustering C	0.278	0.015	0.305	0.018	1.300	0.255	8.293	0.831	16				-0.213
Hippocampus_R'	Clustering C	0.410	0.006	0.418	0.005	0.864	0.353	8.293	0.915	0				-0.107

ParaHippocampal_L'	Clustering C	0.282	0.026	0.358	0.026	4.006	0.046	8.293	0.250	20	-1.149
ParaHippocampal_R'	Clustering C	0.459	0.014	0.480	0.014	1.092	0.297	8.293	0.863	6	-0.172
Amygdala_L'	Clustering C	0.295	0.036	0.211	0.030	2.954	0.087	8.293	0.447	20	0.760
Amygdala_R'	Clustering C	0.423	0.033	0.516	0.035	3.924	0.049	8.293	0.285	10	-1.068
Calcarine_L'	Clustering C	0.447	0.014	0.460	0.014	1.122	0.291	8.293	0.781	14	-0.278
Calcarine_R'	Clustering C	0.554	0.014	0.506	0.014	5.879	0.016	8.293	0.082	16	1.740
Cuneus_L'	Clustering C	0.588	0.010	0.568	0.009	2.325	0.129	8.293	0.462	2	0.735
Cuneus_R'	Clustering C	0.574	0.014	0.595	0.014	0.719	0.397	8.293	0.912	10	-0.111
Lingual_L'	Clustering C	0.397	0.006	0.391	0.006	0.642	0.424	8.293	0.925	2	0.094
Lingual_R'	Clustering C	0.336	0.007	0.350	0.007	2.220	0.137	8.293	0.551	6	-0.596
Occipital_Sup_L'	Clustering C	0.387	0.011	0.407	0.011	2.456	0.118	8.293	0.439	20	-0.774
Occipital_Sup_R'	Clustering C	0.389	0.006	0.391	0.006	0.398	0.529	8.293	0.980	6	-0.025
Occipital_Mid_L'	Clustering C	0.385	0.007	0.369	0.006	3.127	0.078	8.293	0.289	8	1.061
Occipital_Mid_R'	Clustering C	0.528	0.009	0.511	0.008	2.053	0.153	8.293	0.487	2	0.694
Occipital_Inf_L'	Clustering C	0.628	0.017	0.682	0.016	5.517	0.020	8.293	0.082	18	-1.740
Occipital_Inf_R'	Clustering C	0.658	0.012	0.670	0.010	0.747	0.388	8.293	0.917	2	-0.105
Fusiform_L'	Clustering C	0.568	0.014	0.523	0.011	6.803	0.010	8.293	0.040	4	2.053
Fusiform_R'	Clustering C	0.435	0.008	0.455	0.010	2.030	0.156	8.293	0.579	6	-0.554
Postcentral_L'	Clustering C	0.310	0.007	0.320	0.007	0.721	0.397	8.293	0.942	18	-0.073
Postcentral_R'	Clustering C	0.274	0.007	0.263	0.007	1.160	0.283	8.293	0.826	20	0.219
Parietal_Sup_L'	Clustering C	0.285	0.007	0.294	0.006	1.305	0.254	8.293	0.778	20	-0.282
Parietal_Sup_R'	Clustering C	0.332	0.005	0.343	0.004	2.911	0.089	8.293	0.392	6	-0.855
Parietal_Inf_L'	Clustering C	0.489	0.012	0.467	0.009	1.857	0.174	8.293	0.571	18	0.567
Parietal_Inf_R'	Clustering C	0.638	0.015	0.617	0.016	0.994	0.320	8.293	0.890	10	0.139
SupraMarginal_L'	Clustering C	0.580	0.010	0.612	0.010	5.596	0.019	8.293	0.109	6	-1.605
SupraMarginal_R'	Clustering C	0.551	0.009	0.581	0.009	7.082	0.008	8.293	0.050	4	-1.959
Angular_L'	Clustering C	0.657	0.013	0.679	0.013	0.830	0.363	8.293	0.886	6	-0.143
Angular_R'	Clustering C	0.392	0.009	0.424	0.011	5.811	0.017	8.293	0.083	16	-1.731
Precuneus_L'	Clustering C	0.229	0.004	0.218	0.003	6.026	0.015	8.293	0.104	14	1.628
Precuneus_R'	Clustering C	0.248	0.003	0.258	0.003	6.649	0.011	8.293	0.050	8	-1.959
Paracentral_Lobule_L'	Clustering C	0.465	0.007	0.478	0.006	1.760	0.186	8.293	0.713	2	-0.368
Paracentral_Lobule_R'	Clustering C	0.489	0.009	0.508	0.009	1.465	0.227	8.293	0.748	8	-0.321
Caudate_L'	Clustering C	0.431	0.016	0.408	0.015	1.321	0.251	8.293	0.795	12	0.260
Caudate_R'	Clustering C	0.444	0.008	0.426	0.007	3.543	0.061	8.293	0.289	0	1.061
Putamen_L'	Clustering C	0.199	0.004	0.206	0.005	2.331	0.128	8.293	0.487	18	-0.694
Putamen_R'	Clustering C	0.292	0.003	0.301	0.004	2.837	0.093	8.293	0.354	2	-0.927
Pallidum_L'	Clustering C	0.546	0.012	0.528	0.011	1.038	0.309	8.293	0.915	0	0.107
Pallidum_R'	Clustering C	0.632	0.029	0.684	0.026	2.098	0.149	8.293	0.624	8	-0.490
Thalamus_L'	Clustering C	0.279	0.005	0.297	0.008	3.763	0.054	8.293	0.237	6	-1.182
Thalamus_R'	Clustering C	0.274	0.012	0.283	0.010	0.326	0.568	8.293	0.985	18	-0.019
Heschl_L'	Clustering C	0.664	0.040	0.620	0.041	0.910	0.341	8.293	0.967	6	0.042
Heschl_R'	Clustering C	0.811	0.031	0.886	0.025	3.972	0.047	8.293	0.289	0	-1.061
Temporal_Sup_L'	Clustering C	0.330	0.013	0.306	0.010	2.322	0.129	8.293	0.553	20	0.594
Temporal_Sup_R'	Clustering C	0.294	0.013	0.271	0.010	1.853	0.175	8.293	0.678	18	0.415
Temporal_Pole_Sup_L'	Clustering C	0.349	0.018	0.382	0.016	1.644	0.201	8.293	0.780	12	-0.280
Temporal_Pole_Sup_R'	Clustering C	0.450	0.016	0.498	0.015	4.987	0.026	8.293	0.189	6	-1.315

Temporal_Mid_L'	Clustering C	0.383	0.005	0.395	0.005	3.380	0.067	8.293	0.217	4			-1.234
Temporal_Mid_R'	Clustering C	0.364	0.009	0.348	0.009	1.460	0.228	8.293	0.656	18			0.445
Temporal_Pole_Mid_L'	Clustering C	0.339	0.012	0.357	0.012	2.147	0.144	8.293	0.623	14			-0.492
Temporal_Pole_Mid_R'	Clustering C	0.338	0.010	0.312	0.009	3.951	0.048	8.293	0.255	14			1.137
Temporal_Inf_L'	Clustering C	0.354	0.008	0.385	0.009	8.204	0.005	8.293	0.025	18			-2.241
Temporal_Inf_R'	Clustering C	0.388	0.010	0.405	0.011	1.026	0.312	8.293	0.810	18			-0.241
Cerebelum_Crus1_L'	Clustering C	0.357	0.011	0.333	0.010	3.341	0.069	8.293	0.319	8			0.997
Cerebelum_Crus1_R'	Clustering C	0.395	0.015	0.370	0.015	1.718	0.191	8.293	0.688	8			0.402
Cerebelum_Crus2_L'	Clustering C	0.481	0.016	0.515	0.015	2.355	0.126	8.293	0.539	6			-0.614
Cerebelum_Crus2_R'	Clustering C	0.324	0.021	0.380	0.022	2.783	0.097	8.293	0.426	18			-0.797
Cerebelum_3_L'	Clustering C	0.008	0.008	0.040	0.016	3.086	0.080	8.293	0.459	20			-0.740
Cerebelum_3_R'	Clustering C	0.005	0.005	0.035	0.016	3.899	0.049	8.293	0.297	18			-1.043
Cerebelum_4_5_L'	Clustering C	0.413	0.019	0.443	0.019	2.114	0.147	8.293	0.583	6			-0.550
Cerebelum_4_5_R'	Clustering C	0.459	0.022	0.513	0.021	3.354	0.068	8.293	0.412	2			-0.820
Cerebelum_6_L'	Clustering C	0.447	0.013	0.420	0.009	2.688	0.102	8.293	0.477	2			0.710
Cerebelum_6_R'	Clustering C	0.327	0.013	0.365	0.013	3.058	0.082	8.293	0.444	4			-0.765
Cerebelum_7b_L'	Clustering C	0.476	0.031	0.544	0.026	3.407	0.066	8.293	0.339	16			-0.956
Cerebelum_7b_R'	Clustering C	0.464	0.035	0.525	0.035	1.566	0.212	8.293	0.788	16			-0.269
Cerebelum_8_L'	Clustering C	0.410	0.009	0.450	0.012	7.874	0.005	8.293	0.037	2			-2.089
Cerebelum_8_R'	Clustering C	0.252	0.010	0.235	0.008	1.656	0.199	8.293	0.693	12			0.395
Cerebelum_9_L'	Clustering C	0.735	0.022	0.773	0.019	2.203	0.139	8.293	0.603	2			-0.521
Cerebelum_9_R'	Clustering C	0.691	0.030	0.566	0.034	7.111	0.008	8.293	0.052	6			1.945
Cerebelum_10_L'	Clustering C	0.539	0.038	0.446	0.037	2.017	0.157	8.293	0.673	18			0.422
Cerebelum_10_R'	Clustering C	0.728	0.027	0.625	0.032	5.555	0.019	8.293	0.132	6			1.507
Vermis_1_2'	Clustering C	0.000	0.000	0.016	0.011	2.797	0.096	8.293	0.376	16			-0.886
Vermis_3'	Clustering C	0.201	0.033	0.146	0.027	1.783	0.183	8.293	0.725	2			0.352
Vermis_4_5'	Clustering C	0.235	0.012	0.290	0.012	11.202	0.001	8.293	0.008	4	2.561	3.062	-2.638
Vermis_6'	Clustering C	0.033	0.016	0.004	0.003	2.996	0.085	8.293	0.437	14			0.777
Vermis_7'	Clustering C	0.165	0.025	0.252	0.030	5.496	0.020	8.293	0.109	6			-1.605
Vermis_8'	Clustering C	0.300	0.032	0.209	0.027	5.102	0.025	8.293	0.192	8			1.305
Vermis_9'	Clustering C	0.593	0.016	0.542	0.017	3.654	0.057	8.293	0.344	0			0.946
Vermis_10'	Clustering C	0.570	0.019	0.527	0.019	2.115	0.147	8.293	0.686	0			0.404
Precentral_L'	Modularity	2.398	0.108	2.624	0.107	2.617	0.107	8.991	0.726	0			-0.350
Precentral_R'	Modularity	3.098	0.182	3.712	0.202	6.358	0.012	8.991	0.129	10			-1.520
Frontal_Sup_L'	Modularity	3.415	0.180	2.984	0.162	4.161	0.042	8.991	0.362	8			0.911
Frontal_Sup_R'	Modularity	3.195	0.177	3.712	0.200	4.548	0.034	8.991	0.311	10			-1.014
Frontal_Sup_Orb_L'	Modularity	3.488	0.175	3.016	0.160	4.011	0.046	8.991	0.394	8			0.852
Frontal_Sup_Orb_R'	Modularity	2.992	0.129	2.608	0.116	4.836	0.029	8.991	0.252	2			1.145
Frontal_Mid_L'	Modularity	4.455	0.295	3.952	0.245	2.081	0.150	8.991	0.825	20			0.221
Frontal_Mid_R'	Modularity	3.065	0.182	3.640	0.201	5.762	0.017	8.991	0.157	10			-1.415
Frontal_Mid_Orb_L'	Modularity	3.415	0.168	3.008	0.156	3.283	0.071	8.991	0.546	8			0.604
Frontal_Mid_Orb_R'	Modularity	4.016	0.260	3.536	0.212	1.974	0.161	8.991	0.850	16			0.189
Frontal_Inf_Oper_L'	Modularity	2.350	0.108	2.616	0.107	3.557	0.060	8.991	0.524	0			-0.637
Frontal_Inf_Oper_R'	Modularity	3.114	0.182	3.616	0.202	4.587	0.033	8.991	0.277	10			-1.087
Frontal_Inf_Tri_L'	Modularity	3.341	0.172	3.024	0.156	2.158	0.143	8.991	0.805	8			0.247
Frontal_Inf_Tri_R'	Modularity	3.089	0.183	3.560	0.199	3.832	0.051	8.991	0.392	10			-0.855

Frontal_Inf_Orb_L'	Modularity	3.382	0.172	3.000	0.157	2.761	0.098	8.991	0.653	8	0.450
Frontal_Inf_Orb_R'	Modularity	4.130	0.276	3.504	0.214	3.397	0.067	8.991	0.491	16	0.689
Rolandic_Oper_L'	Modularity	2.398	0.108	2.608	0.106	2.381	0.124	8.991	0.760	0	-0.306
Rolandic_Oper_R'	Modularity	3.114	0.199	3.656	0.215	2.810	0.095	8.991	0.611	14	-0.509
Supp_Motor_Area_L'	Modularity	3.447	0.201	2.944	0.157	3.867	0.050	8.991	0.392	8	0.855
Supp_Motor_Area_R'	Modularity	3.187	0.179	3.704	0.195	4.722	0.031	8.991	0.306	10	-1.025
Olfactory_L'	Modularity	4.878	0.310	3.984	0.239	5.400	0.021	8.991	0.215	20	1.239
Olfactory_R'	Modularity	3.683	0.228	4.432	0.257	4.192	0.042	8.991	0.399	18	-0.843
Frontal_Sup_Medial_L'	Modularity	3.455	0.193	3.032	0.167	2.821	0.094	8.991	0.668	8	0.429
Frontal_Sup_Medial_R'	Modularity	3.220	0.175	2.840	0.151	2.909	0.089	8.991	0.664	8	0.434
Frontal_Med_Orb_L'	Modularity	3.431	0.184	2.864	0.154	5.954	0.015	8.991	0.139	8	1.481
Frontal_Med_Orb_R'	Modularity	3.455	0.190	3.008	0.165	3.598	0.059	8.991	0.489	8	0.692
Rectus_L'	Modularity	4.691	0.310	4.176	0.245	2.279	0.132	8.991	0.798	20	0.256
Rectus_R'	Modularity	4.041	0.236	3.376	0.203	3.840	0.051	8.991	0.474	12	0.716
Insula_L'	Modularity	2.398	0.108	2.624	0.106	2.688	0.102	8.991	0.701	0	-0.384
Insula_R'	Modularity	3.935	0.252	3.432	0.202	2.388	0.124	8.991	0.723	16	0.355
Cingulum_Ant_L'	Modularity	3.480	0.191	3.032	0.165	3.245	0.073	8.991	0.546	8	0.604
Cingulum_Ant_R'	Modularity	3.398	0.181	2.896	0.151	4.785	0.030	8.991	0.264	8	1.118
Cingulum_Mid_L'	Modularity	3.797	0.255	3.352	0.201	2.409	0.122	8.991	0.745	16	0.326
Cingulum_Mid_R'	Modularity	2.829	0.137	2.600	0.105	2.215	0.138	8.991	0.803	2	0.249
Cingulum_Post_L'	Modularity	4.057	0.301	3.432	0.220	3.476	0.063	8.991	0.528	14	0.632
Cingulum_Post_R'	Modularity	2.992	0.140	2.584	0.112	5.257	0.023	8.991	0.217	2	1.234
Hippocampus_L'	Modularity	3.911	0.236	3.168	0.190	5.700	0.018	8.991	0.170	14	1.371
Hippocampus_R'	Modularity	3.577	0.214	3.096	0.187	3.307	0.070	8.991	0.543	10	0.609
ParaHippocampal_L'	Modularity	4.089	0.285	3.240	0.190	6.201	0.013	8.991	0.122	14	1.547
ParaHippocampal_R'	Modularity	4.650	0.281	4.080	0.249	2.869	0.092	8.991	0.608	20	0.513
Amygdala_L'	Modularity	2.374	0.107	2.688	0.107	4.977	0.027	8.991	0.230	0	-1.199
Amygdala_R'	Modularity	4.772	0.230	3.872	0.238	7.554	0.006	8.991	0.067	18	1.833
Calcarine_L'	Modularity	2.374	0.105	2.712	0.108	5.633	0.018	8.991	0.177	0	-1.350
Calcarine_R'	Modularity	3.569	0.212	3.088	0.188	2.967	0.086	8.991	0.609	10	0.511
Cuneus_L'	Modularity	4.065	0.267	3.472	0.227	3.184	0.076	8.991	0.574	14	0.562
Cuneus_R'	Modularity	3.528	0.213	3.168	0.183	1.773	0.184	8.991	0.898	10	0.128
Lingual_L'	Modularity	2.374	0.107	2.720	0.106	5.875	0.016	8.991	0.124	0	-1.540
Lingual_R'	Modularity	4.390	0.223	3.728	0.223	4.605	0.033	8.991	0.285	18	1.068
Occipital_Sup_L'	Modularity	2.797	0.125	2.504	0.115	2.448	0.119	8.991	0.746	2	0.324
Occipital_Sup_R'	Modularity	3.829	0.248	3.248	0.195	3.552	0.061	8.991	0.479	16	0.708
Occipital_Mid_L'	Modularity	2.390	0.106	2.680	0.108	4.086	0.044	8.991	0.387	0	-0.865
Occipital_Mid_R'	Modularity	4.276	0.222	3.560	0.227	5.209	0.023	8.991	0.180	18	1.340
Occipital_Inf_L'	Modularity	2.374	0.105	2.728	0.109	6.147	0.014	8.991	0.142	0	-1.469
Occipital_Inf_R'	Modularity	4.463	0.229	3.752	0.222	5.221	0.023	8.991	0.204	18	1.271
Fusiform_L'	Modularity	2.390	0.106	2.672	0.108	3.934	0.048	8.991	0.416	0	-0.814
Fusiform_R'	Modularity	3.967	0.254	3.240	0.193	5.902	0.016	8.991	0.135	16	1.494
Postcentral_L'	Modularity	2.390	0.108	2.608	0.106	2.493	0.116	8.991	0.743	0	-0.328
Postcentral_R'	Modularity	4.285	0.257	3.544	0.213	4.432	0.036	8.991	0.262	18	1.121
Parietal_Sup_L'	Modularity	2.423	0.107	2.600	0.108	1.655	0.200	8.991	0.917	0	-0.105
Parietal_Sup_R'	Modularity	3.870	0.266	3.128	0.209	4.987	0.026	8.991	0.194	16	1.300

Parietal_Inf_L'	Modularity	2.390	0.108	2.624	0.107	2.808	0.095	8.991	0.678	0	-0.415
Parietal_Inf_R'	Modularity	4.236	0.257	3.424	0.204	5.824	0.017	8.991	0.137	18	1.487
SupraMarginal_L'	Modularity	2.390	0.108	2.608	0.106	2.493	0.116	8.991	0.756	0	-0.310
SupraMarginal_R'	Modularity	4.366	0.261	3.472	0.216	6.203	0.013	8.991	0.102	18	1.636
Angular_L'	Modularity	2.390	0.108	2.656	0.108	3.516	0.062	8.991	0.519	0	-0.645
Angular_R'	Modularity	3.837	0.263	3.072	0.191	5.503	0.020	8.991	0.139	16	1.481
Precuneus_L'	Modularity	3.919	0.282	3.208	0.195	5.067	0.025	8.991	0.252	14	1.145
Precuneus_R'	Modularity	2.764	0.134	2.448	0.108	3.874	0.050	8.991	0.406	2	0.832
Paracentral_Lobule_L'	Modularity	3.927	0.257	3.320	0.210	3.695	0.056	8.991	0.474	16	0.716
Paracentral_Lobule_R'	Modularity	3.252	0.186	3.664	0.193	2.950	0.087	8.991	0.639	10	-0.469
Caudate_L'	Modularity	3.431	0.172	2.944	0.156	4.305	0.039	8.991	0.352	8	0.930
Caudate_R'	Modularity	3.057	0.182	3.552	0.199	4.330	0.038	8.991	0.334	10	-0.966
Putamen_L'	Modularity	3.756	0.224	3.296	0.190	2.985	0.085	8.991	0.614	14	0.504
Putamen_R'	Modularity	3.886	0.261	3.232	0.195	4.077	0.045	8.991	0.357	16	0.921
Pallidum_L'	Modularity	3.569	0.183	3.016	0.148	5.980	0.015	8.991	0.122	8	1.547
Pallidum_R'	Modularity	4.106	0.277	3.336	0.205	5.427	0.021	8.991	0.170	16	1.371
Thalamus_L'	Modularity	3.699	0.238	3.216	0.195	2.732	0.100	8.991	0.674	14	0.420
Thalamus_R'	Modularity	3.976	0.289	3.448	0.214	2.621	0.107	8.991	0.686	16	0.404
Heschl_L'	Modularity	3.041	0.171	2.624	0.121	4.478	0.035	8.991	0.321	6	0.993
Heschl_R'	Modularity	4.374	0.269	3.680	0.223	4.355	0.038	8.991	0.349	16	0.937
Temporal_Sup_L'	Modularity	2.390	0.108	2.648	0.107	3.356	0.068	8.991	0.558	0	-0.586
Temporal_Sup_R'	Modularity	4.480	0.252	3.592	0.231	6.835	0.009	8.991	0.077	18	1.770
Temporal_Pole_Sup_L'	Modularity	2.325	0.105	2.680	0.108	6.092	0.014	8.991	0.130	0	-1.513
Temporal_Pole_Sup_R'	Modularity	3.585	0.221	3.080	0.183	3.252	0.073	8.991	0.521	10	0.642
Temporal_Mid_L'	Modularity	2.390	0.108	2.680	0.108	4.125	0.043	8.991	0.379	0	-0.880
Temporal_Mid_R'	Modularity	4.276	0.223	3.488	0.225	6.144	0.014	8.991	0.119	18	1.561
Temporal_Pole_Mid_L'	Modularity	2.374	0.105	2.680	0.108	4.626	0.032	8.991	0.285	0	-1.068
Temporal_Pole_Mid_R'	Modularity	3.878	0.251	3.264	0.192	4.224	0.041	8.991	0.334	16	0.966
Temporal_Inf_L'	Modularity	2.398	0.107	2.680	0.108	3.886	0.050	8.991	0.412	0	-0.820
Temporal_Inf_R'	Modularity	4.285	0.212	3.736	0.226	3.334	0.069	8.991	0.494	18	0.684
Cerebelum_Crus1_L'	Modularity	4.610	0.250	4.008	0.185	3.475	0.064	8.991	0.521	14	0.642
Cerebelum_Crus1_R'	Modularity	3.317	0.154	2.920	0.141	3.791	0.053	8.991	0.469	6	0.724
Cerebelum_Crus2_L'	Modularity	3.398	0.165	2.976	0.142	3.773	0.053	8.991	0.466	6	0.729
Cerebelum_Crus2_R'	Modularity	3.374	0.155	2.952	0.141	4.317	0.039	8.991	0.362	6	0.911
Cerebelum_3_L'	Modularity	3.268	0.162	2.736	0.141	6.395	0.012	8.991	0.122	6	1.547
Cerebelum_3_R'	Modularity	2.821	0.114	2.504	0.105	4.613	0.033	8.991	0.260	0	1.125
Cerebelum_4_5_L'	Modularity	3.439	0.163	3.024	0.139	3.918	0.049	8.991	0.429	6	0.791
Cerebelum_4_5_R'	Modularity	4.374	0.244	3.688	0.191	5.380	0.021	8.991	0.204	14	1.271
Cerebelum_6_L'	Modularity	2.756	0.108	2.528	0.107	2.645	0.105	8.991	0.699	0	0.386
Cerebelum_6_R'	Modularity	3.463	0.165	2.928	0.137	6.537	0.011	8.991	0.110	6	1.597
Cerebelum_7b_L'	Modularity	2.789	0.107	2.520	0.105	3.747	0.054	8.991	0.467	0	0.727
Cerebelum_7b_R'	Modularity	3.390	0.165	2.928	0.147	4.564	0.034	8.991	0.327	6	0.980
Cerebelum_8_L'	Modularity	2.780	0.106	2.520	0.105	3.586	0.059	8.991	0.489	0	0.692
Cerebelum_8_R'	Modularity	3.382	0.162	2.968	0.141	3.961	0.048	8.991	0.402	6	0.837
Cerebelum_9_L'	Modularity	2.789	0.107	2.512	0.106	3.911	0.049	8.991	0.432	0	0.785
Cerebelum_9_R'	Modularity	5.252	0.263	4.584	0.246	3.425	0.065	8.991	0.513	20	0.655

Cerebelum_10_L'	Modularity	2.813	0.109	2.520	0.105	4.347	0.038	8.991	0.372	0	0.892
Cerebelum_10_R'	Modularity	2.813	0.112	2.520	0.105	4.202	0.041	8.991	0.372	0	0.892
Vermis_1_2'	Modularity	2.650	0.137	3.160	0.163	4.645	0.032	8.991	0.304	4	-1.028
Vermis_3'	Modularity	2.577	0.143	2.928	0.139	2.186	0.141	8.991	0.801	4	-0.252
Vermis_4_5'	Modularity	2.780	0.106	2.528	0.104	3.338	0.069	8.991	0.539	0	0.614
Vermis_6'	Modularity	2.764	0.110	2.496	0.103	3.639	0.058	8.991	0.477	0	0.710
Vermis_7'	Modularity	2.797	0.109	2.536	0.104	3.452	0.064	8.991	0.506	0	0.665
Vermis_8'	Modularity	2.789	0.107	2.536	0.106	3.323	0.070	8.991	0.529	0	0.629
Vermis_9'	Modularity	2.772	0.109	2.496	0.102	3.825	0.052	8.991	0.426	0	0.797
Vermis_10'	Modularity	2.780	0.106	2.528	0.103	3.322	0.070	8.991	0.556	0	0.589

Significant GT metrics (per-subnetwork me		SIGNIFICANT RESULTS (p<0.05) IN BOLD													
SUB-NETWC	GT METRIC	PE descriptives		Control descriptives		ANOVA results		MTPC results					Post-hoc t-tests		pseudo-z score
		Mean	SE	Mean	SE	F	p	F crit	P corr	tau	AUC	AUCcrit	t	p	
DMN	Characteristic Path Length	33.544	0.739	35.478	1.574	0.911	0.341	5.011	0.890	2					-0.138
**DMN**	**Density**	**0.014**	**0.000**	**0.014**	**0.000**	**9.226**	**0.003**	**6.325**	**0.016**	**4**	**10.961**	**9.712**	**-3.297**	**.001**	**-2.409**
DMN	Mean Betweeness	23.750	0.301	23.285	0.331	1.034	0.310	6.408	0.840	2					0.202
DMN	Mean Clustering Coefficient	5.733	0.093	6.129	0.227	1.731	0.190	4.211	0.332	0					-0.970
DMN	Global Efficiency	0.174	0.002	0.177	0.002	1.005	0.317	6.732	0.788	6					-0.269
DMN	Smallworldness	Inf	0.000	Inf	0.000	6.894	0.009	6.296	0.040	16	.156	1.421			
RCN	Characteristic Path Length	16.874	0.639	18.459	1.177	1.150	0.285	5.013	0.790	2					-0.266
RCN	Density	0.004	0.000	0.004	0.000	2.336	0.128	6.022	0.384	2					0.871
RCN	Mean Betweeness	1.657	0.026	1.591	0.030	2.633	0.106	7.069	0.538	6					0.616
RCN	Mean Clustering Coefficient	1.979	0.059	2.179	0.129	1.337	0.249	4.401	0.520	8					-0.643
RCN	Global Efficiency	0.065	0.001	0.063	0.001	1.373	0.243	7.620	0.706	2					0.377
RCN	Smallworldness	-390.986	9.089	-376.042	8.711	2.194	0.140	7.059	0.534	2					-0.622
TFM1	Characteristic Path Length	70.779	2.524	82.751	3.729	5.972	0.015	5.650	0.044	12	.065	6.968			-2.014
TFM1	Density	0.002	0.000	0.002	0.000	1.907	0.169	6.593	0.546	14					0.604
TFM1	Mean Betweeness	3.368	0.080	3.568	0.091	2.546	0.112	7.135	0.534	4					-0.622
TFM1	Mean Clustering Coefficient	1.535	0.076	1.433	0.080	0.969	0.326	5.687	0.766	14					0.298
TFM1	Global Efficiency	0.029	0.001	0.026	0.001	3.546	0.061	6.392	0.250	14					1.150
TFM1	Smallworldness	-138.920	7.430	-121.452	6.786	2.665	0.104	7.189	0.438	14					-0.776
TFM2	Characteristic Path Length	80.832	2.375	91.853	4.095	4.393	0.037	6.523	0.218	18					-1.232
TFM2	Density	0.008	0.000	0.008	0.000	2.028	0.156	6.659	0.460	0					-0.739
TFM2	Mean Betweeness	3.661	0.121	3.965	0.139	2.222	0.137	6.844	0.658	8					-0.443
TFM2	Mean Clustering Coefficient	2.497	0.053	2.748	0.148	1.741	0.188	4.870	0.360	0					-0.915
TFM2	Global Efficiency	0.113	0.001	0.117	0.001	3.810	0.052	6.860	0.256	0					-1.136
TFM2	Smallworldness	-259.450	8.126	-242.437	9.125	1.964	0.162	6.706	0.676	18					-0.418
TFM3	Characteristic Path Length	39.677	1.561	42.499	1.839	0.946	0.332	6.626	0.844	8					-0.197
TFM3	Density	0.003	0.000	0.003	0.000	1.597	0.208	7.367	0.558	20					-0.586
TFM3	Mean Betweeness	2.571	0.107	2.382	0.107	1.365	0.244	6.739	0.790	4					0.266
TFM3	Mean Clustering Coefficient	3.047	0.098	3.393	0.139	3.253	0.073	4.559	0.154	12					-1.426
TFM3	Global Efficiency	0.065	0.001	0.069	0.001	2.849	0.093	6.642	0.338	12					-0.958
TFM3	Smallworldness	-358.354	11.992	-384.147	12.126	2.370	0.125	7.926	0.530	12					0.628
TFM4	Characteristic Path Length	137.431	5.647	160.796	7.345	5.365	0.021	7.429	0.148	20					-1.447
TFM4	Density	0.004	0.000	0.004	0.000	1.113	0.292	6.470	0.676	20					-0.418
TFM4	Mean Betweeness	6.404	0.280	7.221	0.308	3.448	0.065	7.847	0.338	14					-0.958
TFM4	Mean Clustering Coefficient	4.227	0.093	4.616	0.219	1.874	0.172	4.754	0.348	2					-0.938
TFM4	Global Efficiency	0.100	0.002	0.096	0.002	2.352	0.126	6.320	0.412	8					0.820
TFM4	Smallworldness	-271.424	9.309	-255.191	9.012	2.372	0.125	6.505	0.566	12					-0.574
TFM6	Characteristic Path Length	80.124	0.918	83.474	3.174	0.685	0.409	4.265	0.938	10					-0.078
TFM6	Density	0.023	0.000	0.023	0.000	6.223	0.013	5.754	0.032	4	.237	5.876			-2.144
TFM6	Mean Betweeness	38.524	0.404	37.459	0.466	2.816	0.095	6.369	0.316	2					1.003
TFM6	Mean Clustering Coefficient	6.310	0.098	6.699	0.304	0.833	0.362	3.374	0.578	0					-0.556
TFM6	Global Efficiency	0.239	0.002	0.243	0.002	1.010	0.316	5.765	0.694	6					-0.393
TFM6	Smallworldness	126.758	55.073	Inf	0.000	1.549	0.214	4.375	0.532	14					
TFM7	Characteristic Path Length	163.542	3.100	173.637	6.231	1.749	0.187	5.524	0.584	18					-0.548
TFM7	Density	0.019	0.000	0.020	0.000	9.142	0.003	6.679	0.012	4	4.385	7.123			-2.512
TFM7	Mean Betweeness	45.030	0.893	46.748	0.804	2.269	0.133	7.185	0.530	10					-0.628
TFM7	Mean Clustering Coefficient	6.314	0.103	6.616	0.251	0.653	0.420	3.705	0.650	0					-0.454
TFM7	Global Efficiency	0.262	0.002	0.265	0.002	0.840	0.360	6.103	0.808	2					-0.243
TFM7	Smallworldness	95.285	9.534	75.579	8.056	1.961	0.163	4.223	0.592	14					0.536
TFM8	Characteristic Path Length	15.385	0.451	16.565	0.953	0.749	0.388	4.437	0.888	0					-0.141
TFM8	Density	0.012	0.000	0.012	0.000	5.451	0.020	6.355	0.078	12					-1.762
TFM8	Mean Betweeness	29.633	0.376	28.807	0.408	2.083	0.150	7.131	0.514	0					0.653
TFM8	Mean Clustering Coefficient	5.744	0.092	6.086	0.285	0.687	0.408	3.166	0.694	0					-0.393

TFM8	Global Efficiency	0.157	0.002	0.161	0.002	0.733	0.393	5.736	0.842	16					-0.199
TFM8	Smallworldness	237.576	9.500	203.413	6.917	6.905	0.009	6.237	0.030	12	.191	.845			2.170
TFM9	Characteristic Path Length	39.595	2.339	45.731	2.610	2.633	0.106	6.052	0.330	10					-0.974
TFM9	Density	0.001	0.000	0.001	0.000	0.690	0.407	6.817	0.884	4					0.146
TFM9	Mean Betweeness	0.135	0.012	0.154	0.015	0.882	0.349	5.839	0.848	20					-0.192
TFM9	Mean Clustering Coefficient	0.249	0.062	0.194	0.059	0.957	0.329	5.680	0.624	20					0.490
TFM9	Global Efficiency	0.004	0.001	0.002	0.001	2.261	0.134	6.234	0.496	20					0.681
TFM9	Smallworldness	-128.335	27.854	-76.924	19.292	2.821	0.094	6.277	0.380	20					-0.878
TFM10	Characteristic Path Length	102.367	2.143	109.346	3.605	2.168	0.142	5.535	0.548	12					-0.601
TFM10	Density	0.013	0.000	0.013	0.000	1.613	0.205	5.250	0.422	4					-0.803
TFM10	Mean Betweeness	17.756	0.282	18.573	0.312	3.889	0.050	6.614	0.222	12					-1.221
TFM10	Mean Clustering Coefficient	4.695	0.084	5.071	0.237	1.491	0.223	3.990	0.368	0					-0.900
TFM10	Global Efficiency	0.163	0.002	0.167	0.002	1.669	0.198	6.667	0.590	6					-0.539
TFM10	Smallworldness	233.185	27.662	191.480	7.290	1.668	0.198	7.030	0.848	4					0.192
TFM11	Characteristic Path Length	128.867	2.272	123.606	4.175	1.530	0.217	4.928	0.640	14					0.468
**TFM11**	**Density**	**0.013**	**0.000**	**0.014**	**0.000**	**9.617**	**0.002**	**5.714**	**0.002**	**6**	**22.319**	**10.769**	**-3.272**	**.001**	**-3.090**
TFM11	Mean Betweeness	24.040	0.327	23.299	0.306	2.777	0.097	6.778	0.432	10					0.786
TFM11	Mean Clustering Coefficient	5.500	0.089	5.898	0.281	1.110	0.293	3.172	0.484	0					-0.700
TFM11	Global Efficiency	0.179	0.002	0.183	0.002	2.170	0.142	6.343	0.420	6					-0.806
TFM11	Smallworldness	233.243	11.582	200.872	6.151	5.268	0.023	6.391	0.084	10					1.728
TFM12	Characteristic Path Length	102.739	2.430	110.408	4.027	2.311	0.130	6.213	0.434	18					-0.782
TFM12	Density	0.007	0.000	0.008	0.000	7.722	0.006	6.647	0.020	0	.481	1.924			-2.326
TFM12	Mean Betweeness	5.119	0.142	5.464	0.153	2.789	0.096	7.347	0.376	18					-0.885
TFM12	Mean Clustering Coefficient	3.627	0.077	3.853	0.190	0.668	0.415	3.592	0.704	6					-0.380
TFM12	Global Efficiency	0.102	0.001	0.104	0.001	0.807	0.370	6.359	0.856	6					-0.181
TFM12	Smallworldness	-593.472	19.467	-664.379	25.915	4.296	0.039	6.598	0.178	2					1.347
TFM13	Characteristic Path Length	39.176	0.925	43.116	1.458	4.580	0.033	5.509	0.118	2					-1.563
TFM13	Density	0.008	0.000	0.008	0.000	1.536	0.216	6.017	0.556	18					-0.589
TFM13	Mean Betweeness	21.390	0.347	21.888	0.355	1.181	0.278	7.009	0.874	2					-0.159
TFM13	Mean Clustering Coefficient	7.627	0.179	8.197	0.267	2.162	0.143	4.590	0.266	20					-1.112
TFM13	Global Efficiency	0.173	0.002	0.171	0.002	0.782	0.377	7.036	0.876	2					0.156
TFM13	Smallworldness	Inf	0.000	Inf	0.000	2.997	0.085	7.679	0.620	12					
TFM14	Characteristic Path Length	130.917	3.249	136.564	5.730	0.291	0.590	5.701	0.982	14					-0.023
TFM14	Density	0.010	0.000	0.010	0.000	1.457	0.229	6.252	0.572	4					-0.565
TFM14	Mean Betweeness	12.710	0.490	11.913	0.577	1.921	0.167	8.567	0.684	18					0.407
TFM14	Mean Clustering Coefficient	4.553	0.078	4.826	0.168	1.514	0.220	5.234	0.428	2					-0.793
TFM14	Global Efficiency	0.141	0.002	0.146	0.002	3.975	0.047	6.808	0.240	4					-1.175
TFM14	Smallworldness	Inf	0.000	Inf	0.000	1.511	0.220	8.023	0.880	6					
TFM16	Characteristic Path Length	73.300	3.670	86.085	6.196	2.541	0.112	5.995	0.370	8					-0.896
TFM16	Density	0.005	0.000	0.005	0.000	2.588	0.109	6.668	0.372	0					-0.893
TFM16	Mean Betweeness	8.835	0.256	9.315	0.212	2.112	0.147	6.340	0.568	0					-0.571
TFM16	Mean Clustering Coefficient	2.759	0.129	2.938	0.130	0.525	0.469	5.786	0.826	16					-0.220
TFM16	Global Efficiency	0.074	0.002	0.079	0.002	3.495	0.063	7.586	0.318	2					-0.999
TFM16	Smallworldness	-211.124	7.554	-221.458	8.169	0.765	0.383	7.422	0.916	4					0.105
TFM17	Characteristic Path Length	21.046	1.159	22.956	1.365	0.843	0.360	7.045	0.884	0					-0.146
TFM17	Density	0.010	0.000	0.010	0.000	6.020	0.015	6.097	0.052	4					-1.943
TFM17	Mean Betweeness	16.028	0.268	15.396	0.246	2.936	0.088	7.378	0.384	2					0.871
TFM17	Mean Clustering Coefficient	4.259	0.082	4.645	0.211	2.038	0.155	4.592	0.312	0					-1.011
TFM17	Global Efficiency	0.130	0.001	0.133	0.001	1.277	0.259	6.059	0.726	6					-0.350
TFM17	Smallworldness	385.089	46.513	273.984	48.829	2.634	0.106	7.310	0.658	2					0.443
TFM18	Characteristic Path Length	58.259	1.364	64.083	4.672	0.910	0.341	4.103	0.882	10					-0.148
TFM18	Density	0.006	0.000	0.006	0.000	1.547	0.215	5.446	0.572	0					-0.565
TFM18	Mean Betweeness	2.511	0.059	2.618	0.057	1.666	0.198	6.625	0.694	16					-0.393
TFM18	Mean Clustering Coefficient	3.097	0.104	3.434	0.227	1.126	0.290	3.493	0.572	18					-0.565
TFM18	Global Efficiency	0.056	0.002	0.060	0.001	3.204	0.075	6.869	0.312	18					-1.011
TFM18	Smallworldness	-242.195	7.562	-255.355	6.325	1.469	0.227	7.887	0.716	18					0.364

TFM19	Characteristic Path Length	118.273	3.391	128.981	5.411	2.046	0.154	5.708	0.616	10					-0.502
TFM19	Density	0.008	0.000	0.008	0.000	4.434	0.036	6.811	0.132	8					-1.506
**TFM19**	**Mean Betweeness**	**17.202**	**0.498**	**19.994**	**0.555**	**12.325**	**0.001**	**8.157**	**0.010**	**8**	**14.836**	**4.101**	**-3.742**	**.000**	**-2.576**
TFM19	Mean Clustering Coefficient	4.059	0.074	4.299	0.159	1.175	0.279	4.894	0.536	0					-0.619
TFM19	Global Efficiency	0.134	0.002	0.139	0.002	3.460	0.064	8.015	0.356	4					-0.923
TFM19	Smallworldness	-741.781	66.661	Inf	0.000	2.552	0.111	7.453	0.612	12					
TFM20	Characteristic Path Length	191.483	3.927	206.450	7.442	2.954	0.087	5.270	0.348	20					-0.938
TFM20	Density	0.011	0.000	0.012	0.000	5.450	0.020	7.045	0.106	4					-1.616
TFM20	Mean Betweeness	20.365	0.438	21.417	0.441	2.824	0.094	6.459	0.456	20					-0.745
TFM20	Mean Clustering Coefficient	5.704	0.121	6.034	0.255	0.722	0.396	4.114	0.608	6					-0.513
TFM20	Global Efficiency	0.116	0.002	0.114	0.002	0.605	0.438	6.675	0.882	14					0.148
TFM20	Smallworldness	Inf	0.000	Inf	0.000	1.738	0.189	7.675	0.910	12					
TFM21	Characteristic Path Length	23.895	1.057	25.663	1.399	0.986	0.322	6.186	0.866	0					-0.169
TFM21	Density	0.006	0.000	0.006	0.000	2.589	0.109	6.263	0.356	16					-0.923
TFM21	Mean Betweeness	13.062	0.622	11.298	0.421	5.946	0.015	7.291	0.120	6					1.555
TFM21	Mean Clustering Coefficient	6.323	0.140	6.668	0.205	1.126	0.290	4.970	0.502	6					-0.671
TFM21	Global Efficiency	0.103	0.002	0.106	0.002	0.658	0.418	7.737	0.910	12					-0.113
