# Supplementary material for: Schizophrenia‐like topological changes in the structural connectome of individuals with subclinical psychotic experiences
Source: Hum Brain Mapp. 2015 Apr 2;36(7):2629–43. doi: 10.1002/hbm.22796 (PMC4479544; doi:10.1002/hbm.22796)
Supplement: Supplementary file 2 — Supporting Information [file HBM-36-2629-s002.pdf]

Supplementary material

Drakesmith M. *et al.* "Schizophrenia-like topological changes in the structural connectome of individuals with sub-clinical psychotic experiences". *Human Brain Mapping*

1. Group differences in diffusion-tensor metrics. 2
2. Group effects of head motion 4
3. Effects of specific psychotic experiences 5
4. GT metrics across threshold 8
5. Functional sub-networks. 14

1. Group differences in diffusion-tensor metrics.

Additional analysis of standard diffusion tensor measures revealed some limited differences between the PE and control groups.

Method

MRI data was pre-processed as described in the main text. The RESTORE algorithm [Chang et al., 2005] was used to estimate diffusion tensors for each voxel and were corrected for partial volume effects [Pasternak et al., 2009] Fractional anisotropy (FA), axial diffusivity (AD), radial diffusivity (RD) and mean diffusivity (MD) were calculated from diffusion tensors.

Tract based spatial statistics (TBSS) [Smith et al., 2006] was performed in FSL v5.0.1 (http://www.fmrib.ox.ac.uk/fsl), performed by registering the FA images to the FA_FFMRIB template and then skeletonizing. The RD, AD and MD values were then transformed to the same group skeleton. Group comparisons were performed with correction for multiple comparisons using permutation tests (500 randomizations) and threshold-free cluster enhancement [Smith and Nichols, 2009].

In addition, a comparison of the gross white matter volume was performed, derived from the white matter compartment in the segmented T1-weighted structural.

Results

A small region (59 voxels) in the left fronto-medial white matter was found to have significant effects. FA and AD were significantly lower in the PE group compared to controls while MD was significantly higher in the PE group. Fibre pathways traversing the significant region include those of the genu, the left cingulum and the left anterior thalamic radiations.

The same comparison of gross white matter volume showed no significant differences (F=0.51, p=0.48).

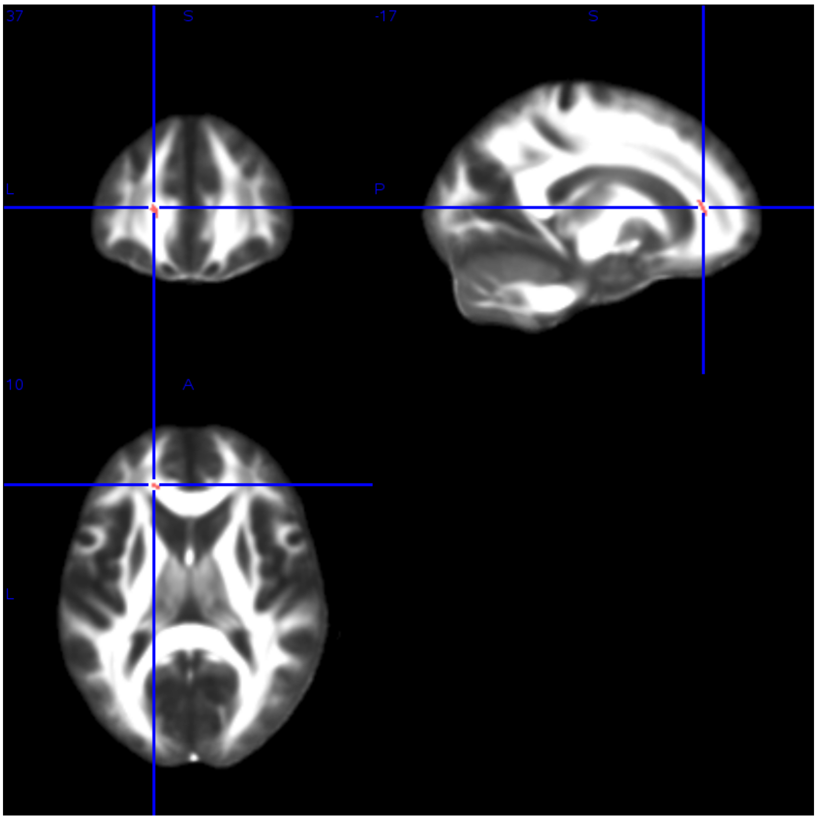

Figure S1.1. TBSS results showing significant increases in FA and AD and decreases in MD (p_{corr}<0.05) in the left fronto-medial white matter.

References

Chang L-C, Jones DK, Pierpaoli C (2005): RESTORE: robust estimation of tensors by outlier rejection. Magn Reson Med 53:1088–95.

Pasternak O, Sochen N, Gur Y, Intrator N, Assaf Y (2009): Free water elimination and mapping from diffusion MRI. Magn Reson Med 62:717–30.

Smith SM, Jenkinson M, Johansen-Berg H, Rueckert D, Nichols TE, Mackay CE, Watkins KE, Ciccarelli O, Cader MZ, Matthews PM, Behrens TEJ (2006): Tract-based spatial statistics: voxelwise analysis of multi-subject diffusion data. Neuroimage 31:1487–505.

Smith SM, Nichols TE (2009): Threshold-free cluster enhancement: addressing problems of smoothing, threshold dependence and localisation in cluster inference. Neuroimage 44:83–98.

2. Group effects of head motion

To ensure group effects observed were not an artefact due to differences in head motion, we quantified head motion as the mean image displacement. Displacement for each DWI was measured by the Euclidian norm of the transform matrix resulting from the rigid body transformations performed during motion correction. Each transform matrix was relative to the mean head position. These mean displacements were compared between the two groups. Box plots showing the distributions of head motion are shown in figure S2.1. An Independent samples *t*-test show no significant differences in head movement between the two groups (t=-1.3, p=0.18).

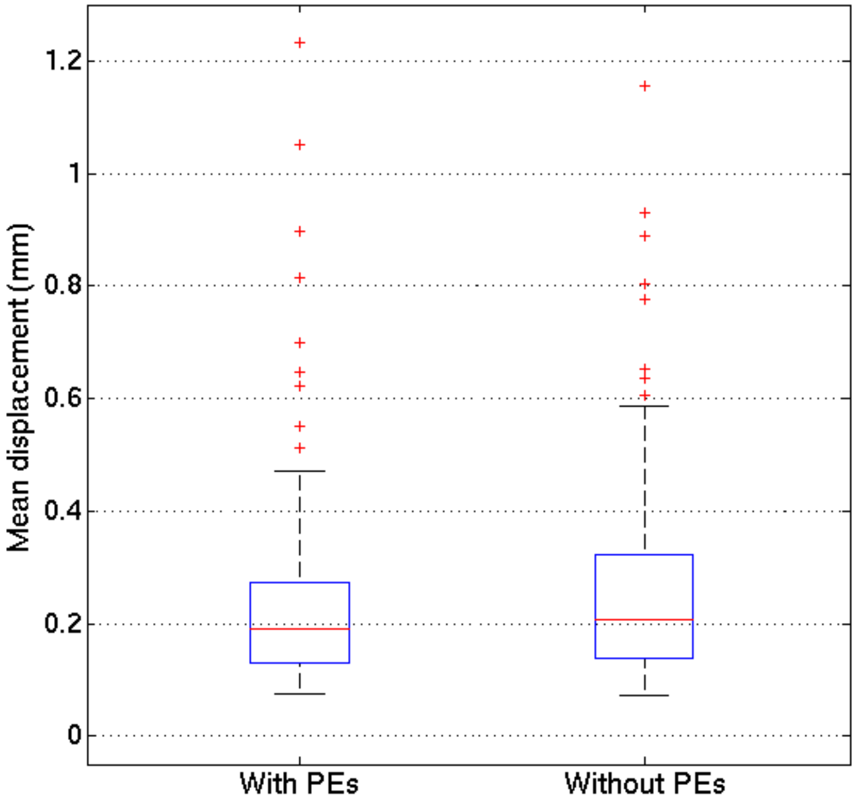

Figure S2.1. Box plot showing mean displacement of head coordinated during the course of the DWI scan for subjects in each group.

3. Effects of specific psychotic experiences

The PLIKS interview consists of several questions probing 7 specific domains of psychosis: unusual experiences, auditory hallucinations, visual hallucinations, delusions, and thought disorder. To assess the impact of different domains of psychosis in the PE group, we tested each item from the PLIKS interview (excluding variables relating to social impact and to drug or alcohol consumption). A χ^2 test was conducted on the response frequencies of each item in each group. The size of the effect was quantified using the $-\log_{10}$ of the resultant *p*-value. Results are shown in figure S3.1 and Table S3.1. The largest effect was seen by items probing auditory hallucinations. The weakest responses were seen for items probing thought disorder and some that probe delusions.

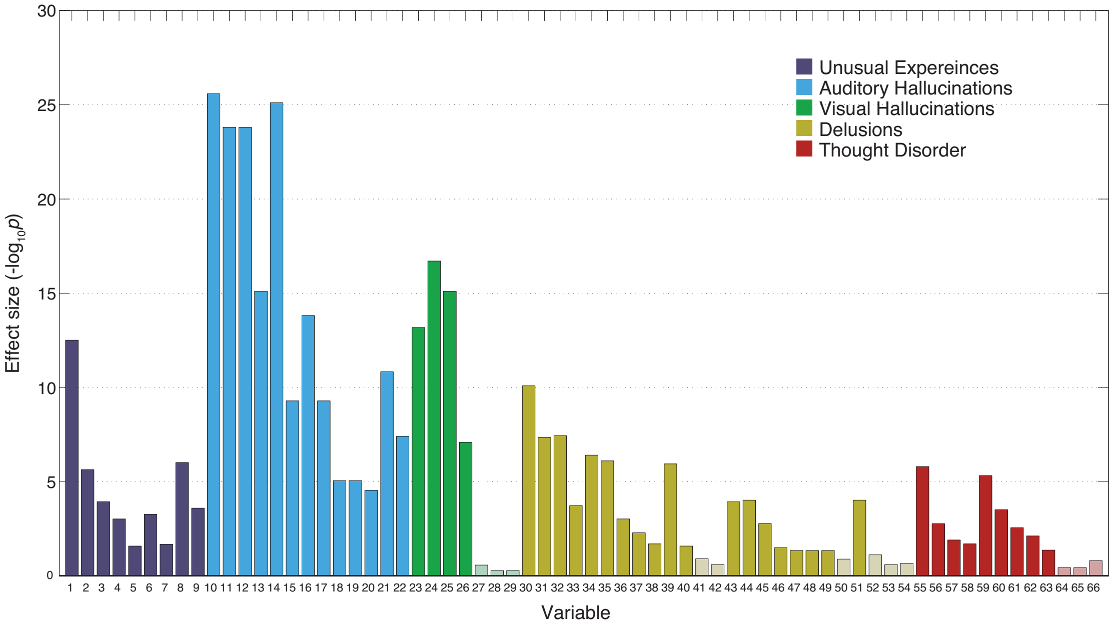

Figure S3.1. χ^2 Effect size ($-\log_{10}$ *p*) for each item on the PLIKS interview. Colour code indicates which domain each item probes. Lighter shades indicate non-significant effects at *p*<0.05.

Table S3.1. Item on the PLIKS interview with corresponding effects tested with χ^2.

Domain / Variable		ALSPAC variable ID	Variable name	Effect
Unusual Experiences	1	FJPL013	YP has felt that things looked/sounded/felt abnormal, or had unusual sensations	χ^2 (5) = 57.605259 p=0.000000
	2	FJPL014	YP has ever felt that the world was unreal, that things around them were like a stage set	χ^2 (5) = 26.004882 p=0.000002
	3	FJPL015	YP has ever felt that they were not a real person, not part of the living world	χ^2 (5) = 18.175822 p=0.000113
	4	FJPL016	YP has ever felt that part of their body did not belong to them, or looked unfamiliar or wrong size	χ^2 (5 = 13.942225 p=0.000939
	5	FJPL017	YP has ever felt that their appearance seemed to change in a way different to ordinary growing up	χ^2 (5) = 7.320447 p=0.025727
	6	FJPL018	YP has ever felt that sometimes they can only see parts of an object, when they should be able to see it all	χ^2 (5) = 15.078869 p=0.000532
	7	FJPL019	YP has ever experienced other unusual sensations, such as things looking/sounding different	χ^2 (5) = 7.766427 p=0.020585
	8	FJPL020	Length of time in a day that YP's unusual experience lasted when at it's worst	χ^2 (9) = 33.475351 p=0.000001
	9	FJPL021	Frequency YP has had unusual experiences, in last 6 months	χ^2 (9) = 21.519662 p=0.000250
Auditory Hallucinations	10	FJPL027	YP has ever heard voices that other people could not hear	χ^2 (5) = 117.814018 p=0.000000
	11	FJPL028	Length of time in a day that voice was present, when at it's worst	χ^2 (9) = 117.814018 p=0.000000
	12	FJPL029	Degree that YP was upset when voice was present, when at it's worst	χ^2 (9) = 117.814018 p=0.000000

	13	FJPL030	Frequency YP has heard the voice, in last 6 months	χ^2 (9) = 76.936243 p=0.000000
	14	FJPL031	Staff rating of auditory hallucinations	χ^2 (5) = 115.638451 p=0.000000
	15	FJPL032	YP felt voices came from inside their head	χ^2 (5) = 42.797975 p=0.000000
	16	FJPL033	YP felt voices came from outside their head	χ^2 (5) = 63.644576 p=0.000000
	17	FJPL034	YP's voices talked directly to them, or told them things	χ^2 (5) = 42.797975 p=0.000000
	18	FJPL035	YP's voices told them to do things (commanded)	χ^2 (5) = 23.315784 p=0.000009
	19	FJPL036	YP's voices talked about what they were doing/feeling/thinking (commentated)	χ^2 (5) = 23.315784 p=0.000009
	20	FJPL037	YP ever hears 2 or more voices talking to each other, or about YP	χ^2 (5) = 20.910995 p=0.000029
	21	FJPL038	YP has heard other noises/voices, sporadic (single word) hallucinations	χ^2 (5) = 49.919485 p=0.000000
	22	FJPL039	YP has heard other noises/voices, elementary (noises such as bangs/bells) hallucinations	χ^2 (7) = 37.338151 p=0.000000
Visual Hallucinations	23	FJPL044	YP has ever seen something or someone that other people could not see	χ^2 (5) = 60.729071 p=0.000000
	24	FJPL045	Staff rating of visual hallucinations	χ^2 (5) = 76.936243 p=0.000000
	25	FJPL046	Length of time in a day that visions were present, when at it's worst	χ^2 (9) = 76.936243 p=0.000000
	26	FJPL047	Frequency YP has seen visions, in last 6 months	χ^2 (9) = 38.684061 p=0.000000
	27	FJPL048	Staff rating of visual illusions	χ^2 (5) = 2.667953 p=0.263428
	28	FJPL049	Length of time in a day that illusions were present, when at their worst	χ^2 (9) = 3.274053 p=0.513054
	29	FJPL050	Frequency YP has seen illusions, in last 6 months	χ^2 (9) = 3.307775 p=0.507701
Delusions	30	FJPL056	YP has ever felt that they were being followed or spied on	χ^2 (5) = 46.481756 p=0.000000
	31	FJPL057	Length of time in a day that YP thought about being followed/spied on, when at it's worst	χ^2 (11) = 42.608109 p=0.000000
	32	FJPL058	Degree that YP was upset when they had thoughts of being followed/spied on, when at it's worst	χ^2 (11) = 43.092933 p=0.000000
	33	FJPL059	Frequency YP has had thoughts of being followed/spied on, in last 6 months	χ^2 (11) = 24.387659 p=0.000183
	34	FJPL060	Staff rating of delusions of being spied on	χ^2 (5) = 29.517322 p=0.000000
	35	FJPL061	YP has ever thought someone was making things hard for them, causing them trouble, trying to hurt them, plotting against them	χ^2 (5) = 28.131158 p=0.000001
	36	FJPL062	Staff rating of delusions of persecution	χ^2 (5) = 13.942225 p=0.000939
	37	FJPL063	Length of time in a day that YP had feelings of persecution, when at it's worst	χ^2 (7) = 12.815213 p=0.005054
	38	FJPL064	Frequency YP has had feelings of persecution, in last 6 months	χ^2 (9) = 11.697712 p=0.019747
	39	FJPL065	YP has ever believed that others have read their thoughts	χ^2 (5) = 27.405089 p=0.000001
	40	FJPL066	Staff rating of delusions of thoughts being read	χ^2 (5) = 7.320447 p=0.025727
	41	FJPL067	Length of time in a day that YP believed that others read their thoughts, when at it's worst	χ^2 (9) = 7.320447 p=0.119892
	42	FJPL068	Frequency YP has believed that others read their thoughts, in last 6 months	χ^2 (7) = 4.131681 p=0.247592
	43	FJPL069	YP has ever thought they were being sent special messages, through TV/Computer/Radio	χ^2 (5) = 18.118523 p=0.000116
	44	FJPL070	Staff rating of delusions of reference	χ^2 (5) = 18.547848 p=0.000094
	45	FJPL071	Length of time in a day that YP thought they were being sent special messages, when at it's worst	χ^2 (9) = 17.381553 p=0.001629
	46	FJPL072	Frequency YP has thought they were being sent special messages, in last 6 months	χ^2 (9) = 10.589602 p=0.031585
	47	FJPL073	YP has ever felt that they were under the control of some special power	χ^2 (5) = 6.248740 p=0.043965

	48	FJPL074	Staff rating of delusions of control	χ^2 (5) = 6.248740 p=0.043965
	49	FJPL075	Length of time in a day that YP thought they were under the control of some special power, when at it's worst	χ^2 (5) = 6.248740 p=0.043965
	50	FJPL076	Frequency YP has thought they were under the control of some special power, in last 6 months	χ^2 (5) = 4.131681 p=0.126712
	51	FJPL077	YP has ever felt that they were very important or had special powers/abilities	χ^2 (5) = 18.547848 p=0.000094
	52	FJPL078	Staff rating of delusions of grandiose ability	χ^2 (5) = 5.185854 p=0.074801
	53	FJPL079	Length of time in a day that YP felt that they were very important or had special powers/abilities, when at it's worst	χ^2 (7) = 4.131681 p=0.247592
	54	FJPL080	Frequency YP has felt that they were very important or had special powers/abilities, in last 6 months	χ^2 (5) = 3.086112 p=0.213727
Though Disorder	55	FJPL084	YP has ever felt that their thoughts were broadcast out loud	χ^2 (5) = 26.757639 p=0.000002
	56	FJPL085	Staff rating of thought broadcasting	χ^2 (5) = 12.815213 p=0.001649
	57	FJPL086	Length of time in a day that YP felt that their thoughts were broadcast out loud, when at it's worst	χ^2 (9) = 12.815213 p=0.012215
	58	FJPL087	Frequency YP has felt that their thoughts were broadcast out loud, in last 6 months	χ^2 (9) = 11.697712 p=0.019747
	59	FJPL088	YP has ever felt that thoughts were put in their mind which were not their own	χ^2 (5) = 24.534139 p=0.000005
	60	FJPL089	Staff rating of thought insertion	χ^2 (5) = 16.225270 p=0.000300
	61	FJPL090	Length of time in a day that YP felt that thoughts were put in their mind, when at it's worst	χ^2 (9) = 16.225270 p=0.002731
	62	FJPL091	Frequency YP has felt that thoughts were put in their mind, in last 6 months	χ^2 (9) = 13.942225 p=0.007482
	63	FJPL092	YP has ever felt that thoughts were taken out of their mind by someone or some special force	χ^2 (3) = 4.131681 p=0.042088
	64	FJPL093	Staff rating of thought withdrawal	χ^2 (5) = 2.049045 p=0.358968
	65	FJPL094	Length of time in a day that YP felt that thoughts were taken out of their mind, when at it's worst	χ^2 (5) = 2.049045 p=0.358968
	66	FJPL095	Frequency YP has felt that thoughts were taken out of their mind, in last 6 months	χ^2 (3) = 2.049045 p=0.152302

4. GT metrics across threshold

These graphs show the mean and standard error of each GT metric across each threshold. Figure S4.1 shows the results of global network metrics. Figure S4.2 shows the mean and standard error of the global efficiency computed at density-based thresholds. Figures S4.3 show the mean and standard error of the node level metrics.

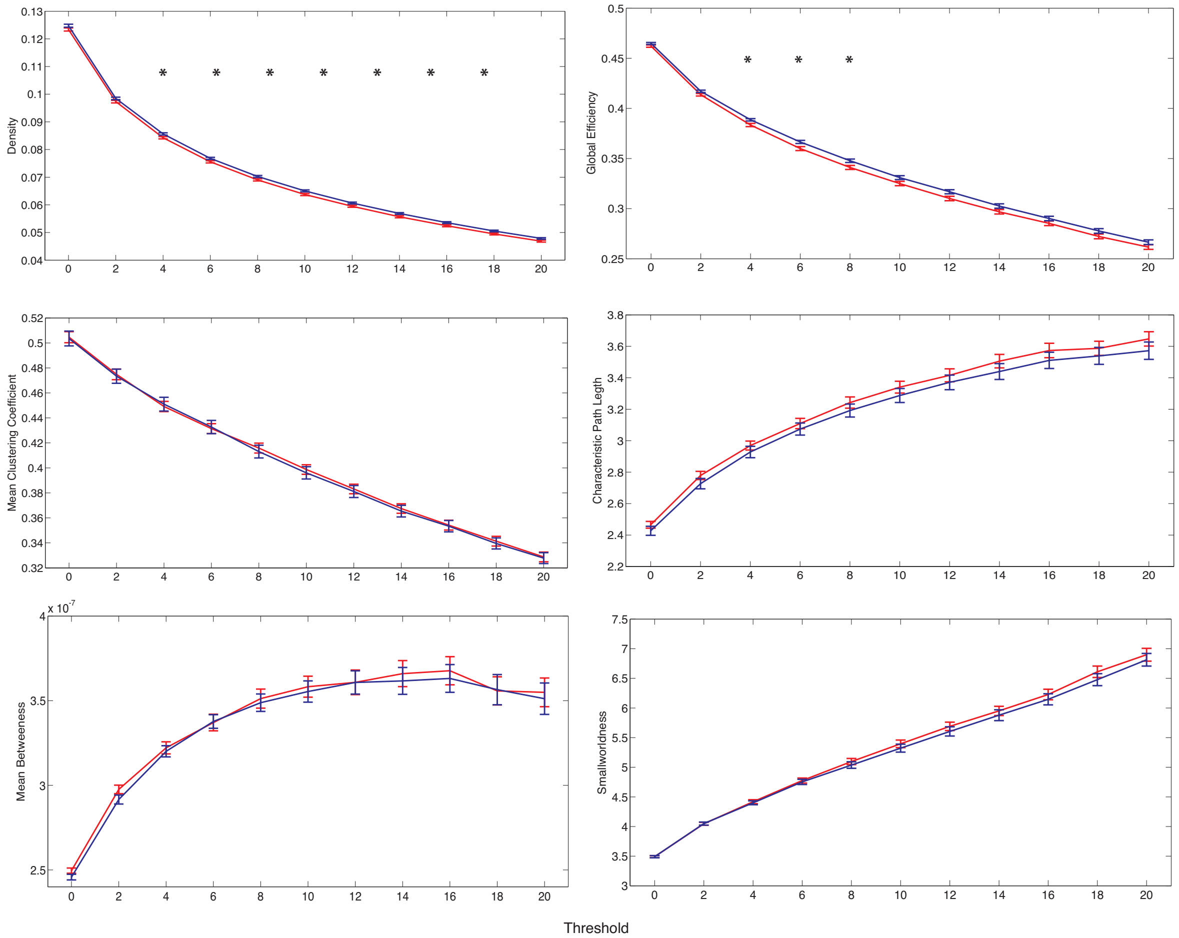

Figure S4.1. Mean and standard error of global network metrics. * indicates results significant at p_{corr}<0.05 following permutation correction for multiple comparisons across thresholds.

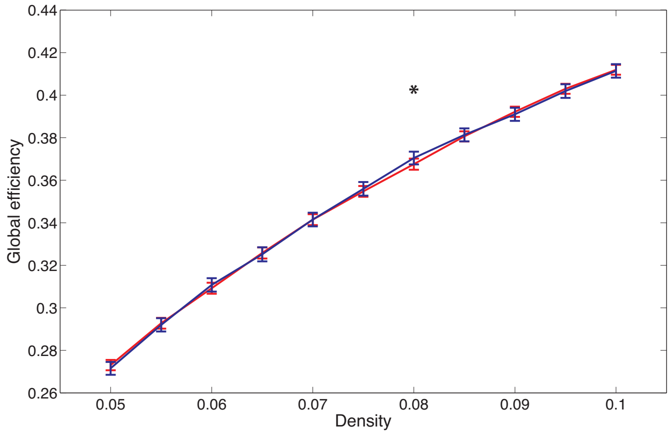

Figure S4.2. Mean and standard error of global efficiency computed across density thresholds. * indicates results significant at p_{corr}<0.05 following permutation correction for multiple comparisons across thresholds.

Figure S4.3 (below). Mean and standard error of local network metrics.

Degree

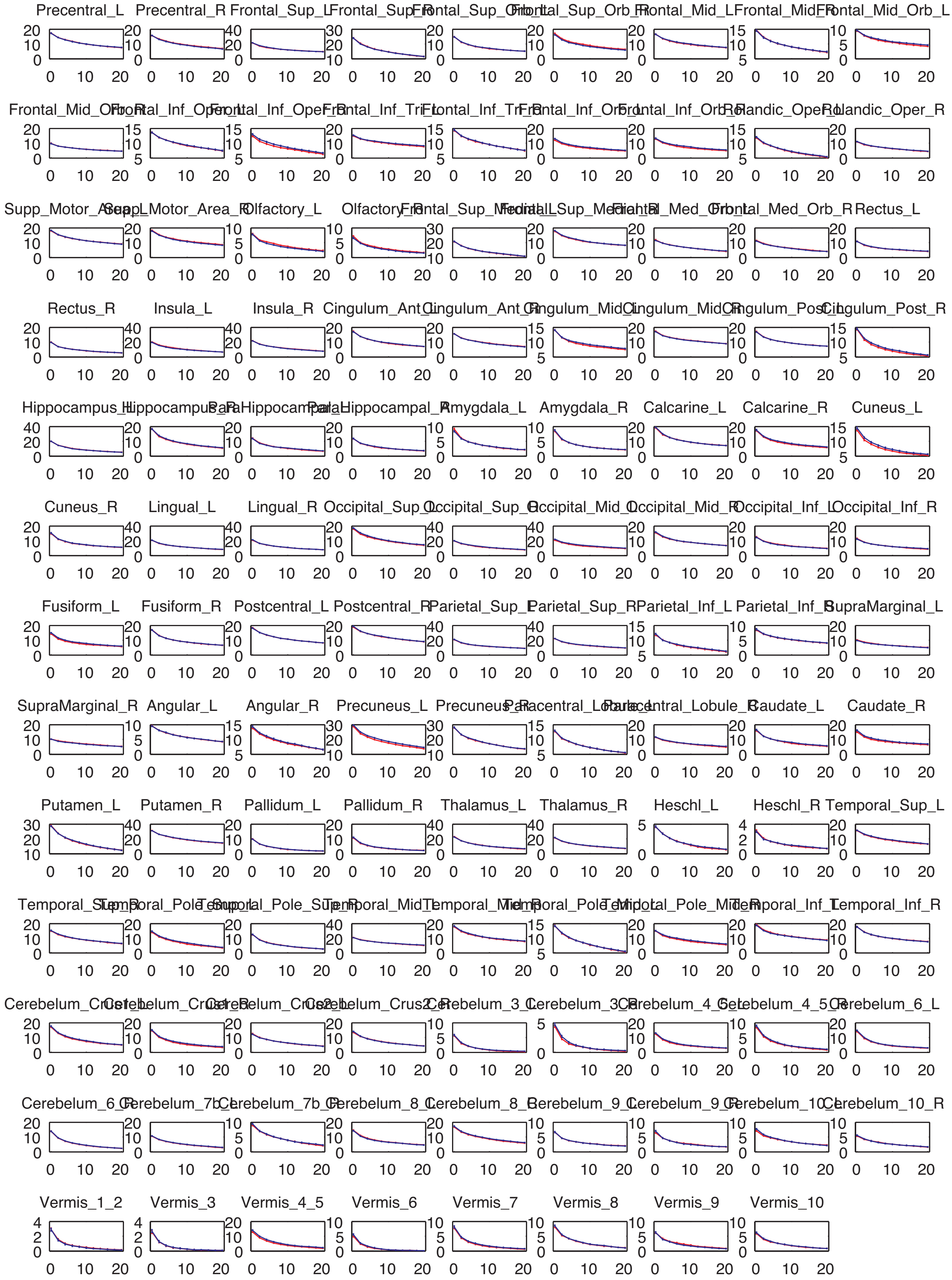

Efficiency

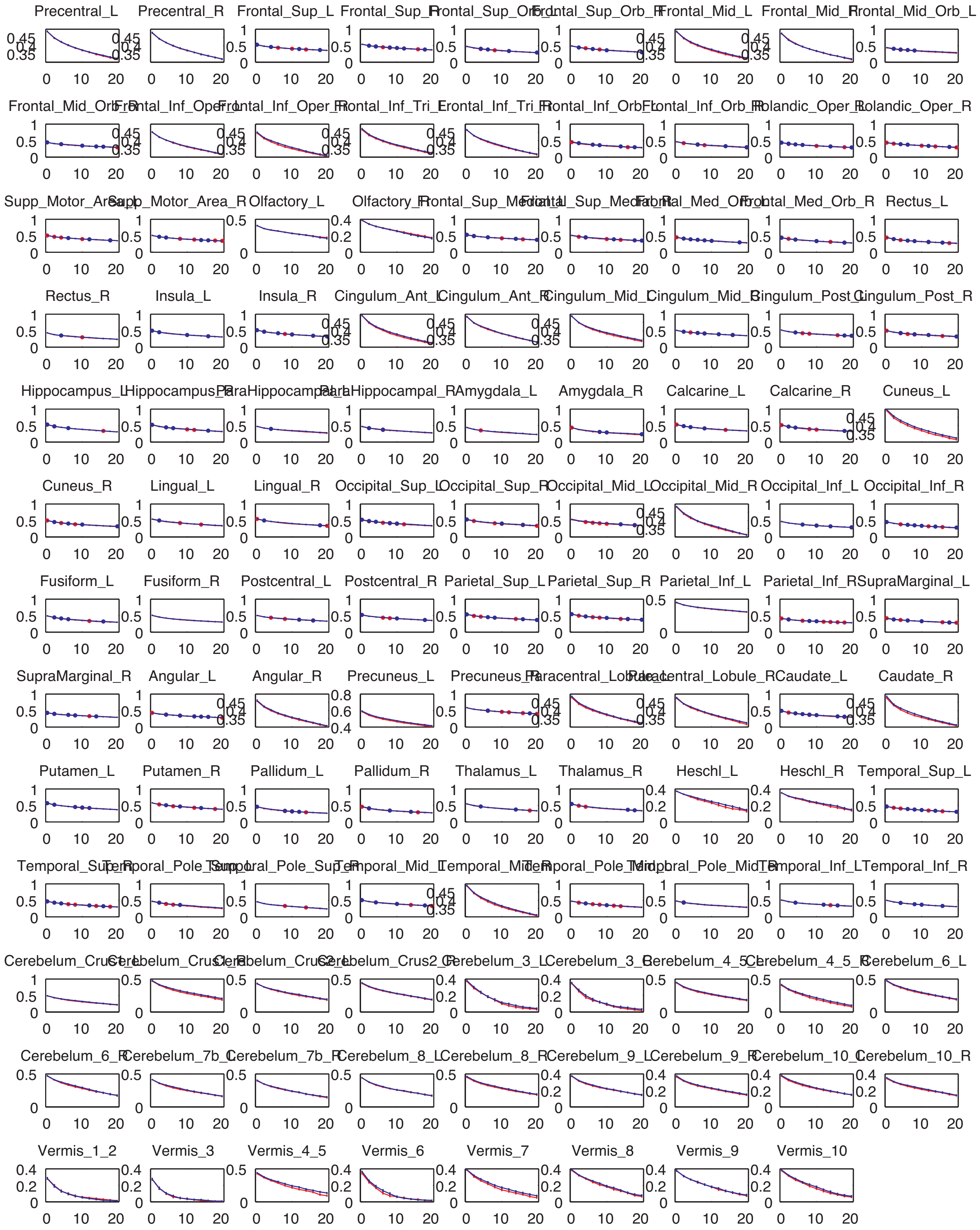
Precentral_L
Precentral_R
Frontal_Sup_L
Frontal_Mid_L
Frontal_Mid_R
Rectus_L
Rectus_R
Insula_L
Insula_R
Calcarine_L
Calcarine_R
Cuneus_L
Cuneus_R
Lingual_L
Lingual_R
Occipital_Inf_L
Occipital_Inf_R
Fusiform_L
Fusiform_R
Postcentral_L
Postcentral_R
Parietal_Sup_L
Parietal_Sup_R
Parietal_Inf_L
SupraMarginal_L
SupraMarginal_R
Angular_L
Angular_R
Precuneus_L
Caudate_L
Caudate_R
Putamen_L
Putamen_R
Pallidum_L
Pallidum_R
Thalamus_L
Thalamus_R
Heschl_L
Heschl_R
Temporal_Sup_L
Vermis_1_2
Vermis_3
Vermis_4_5
Vermis_6
Vermis_7
Vermis_8
Vermis_9
Vermis_10

Betweeness

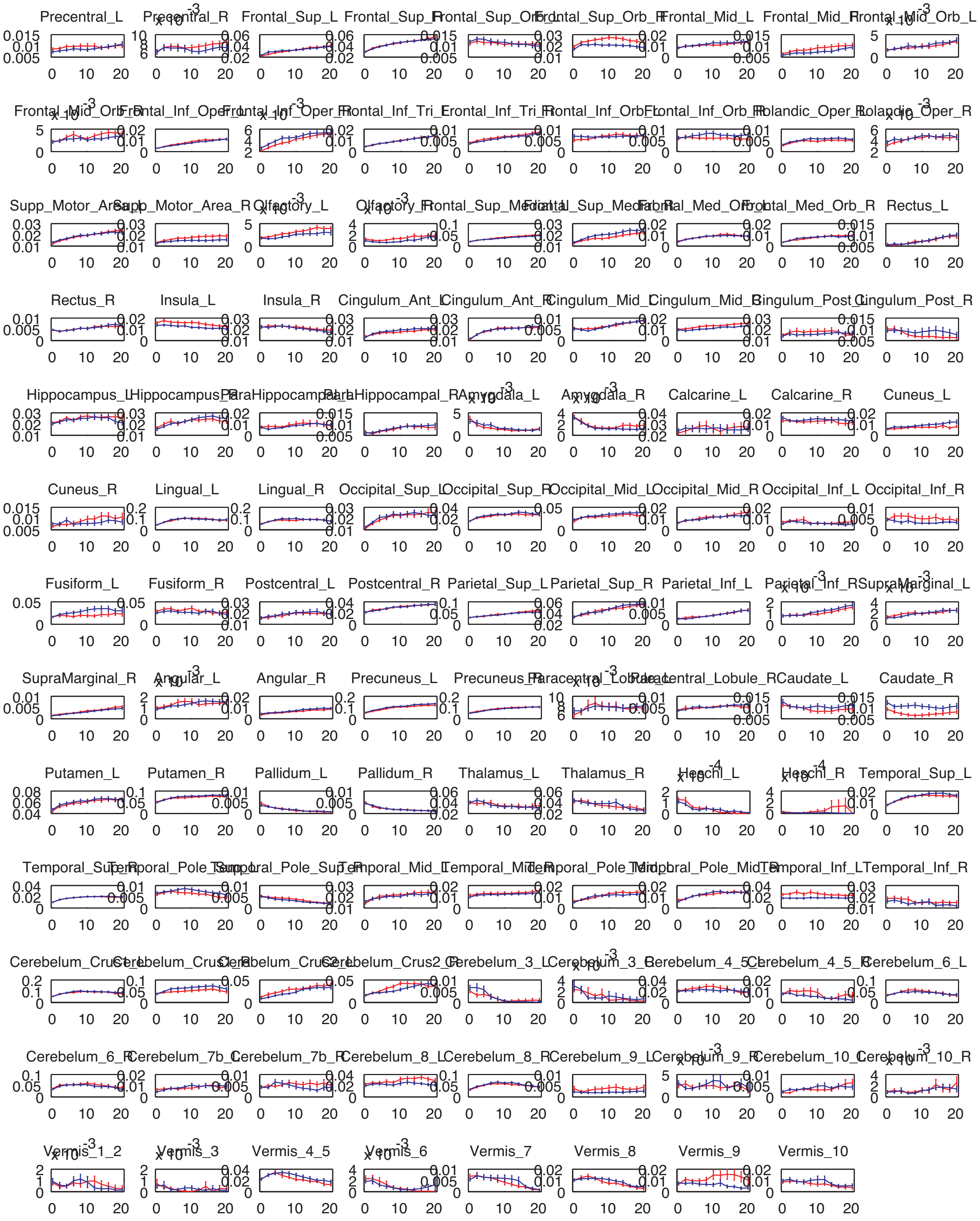

Clustering Coefficient

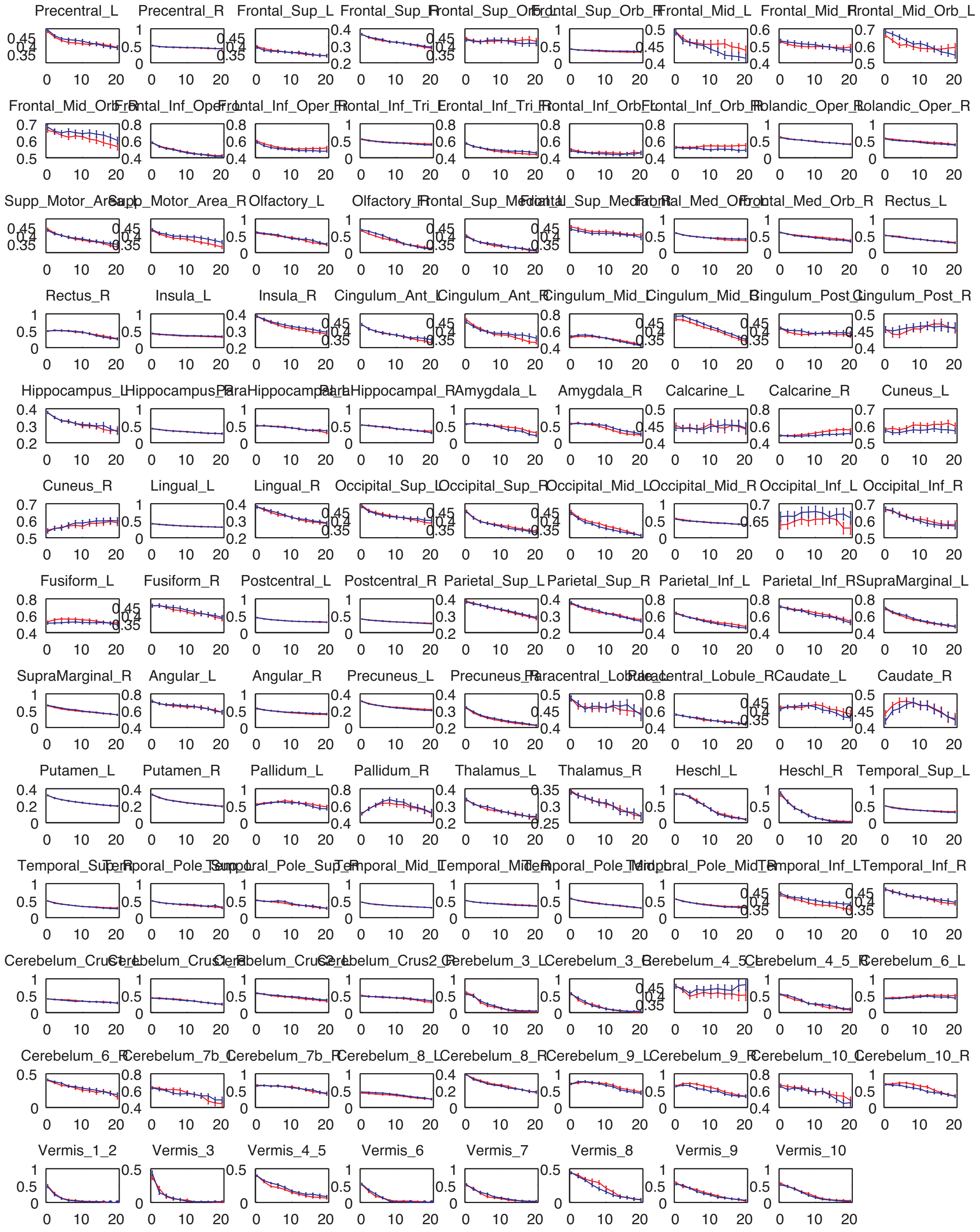

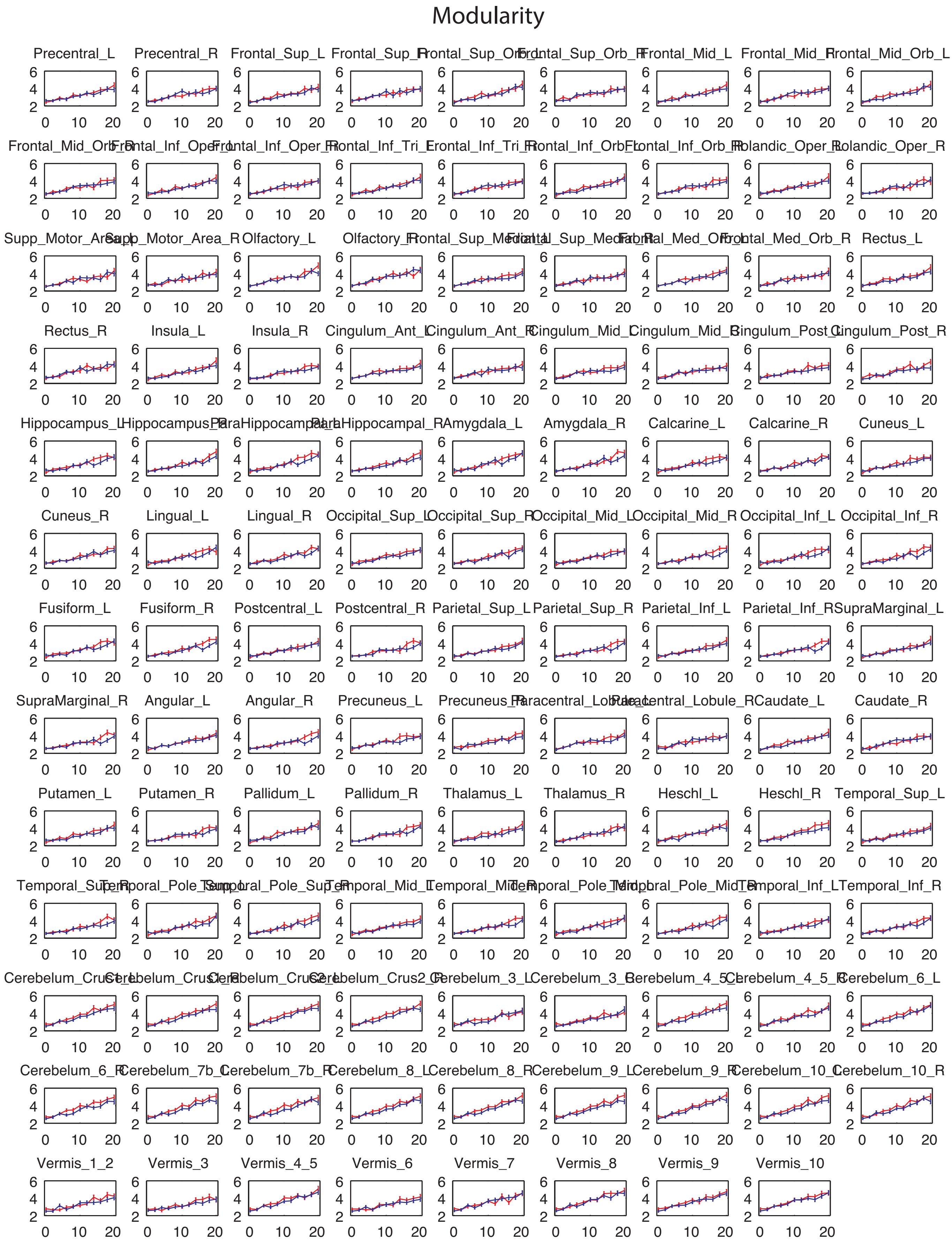
Modularity

5. Functional sub-networks.

All functional sub-networks derived from the networks described in Smith et al., [2012], the default mode network (DMN) and the rich-club network (RCN) for a typical subject are shown in figure S5.1.

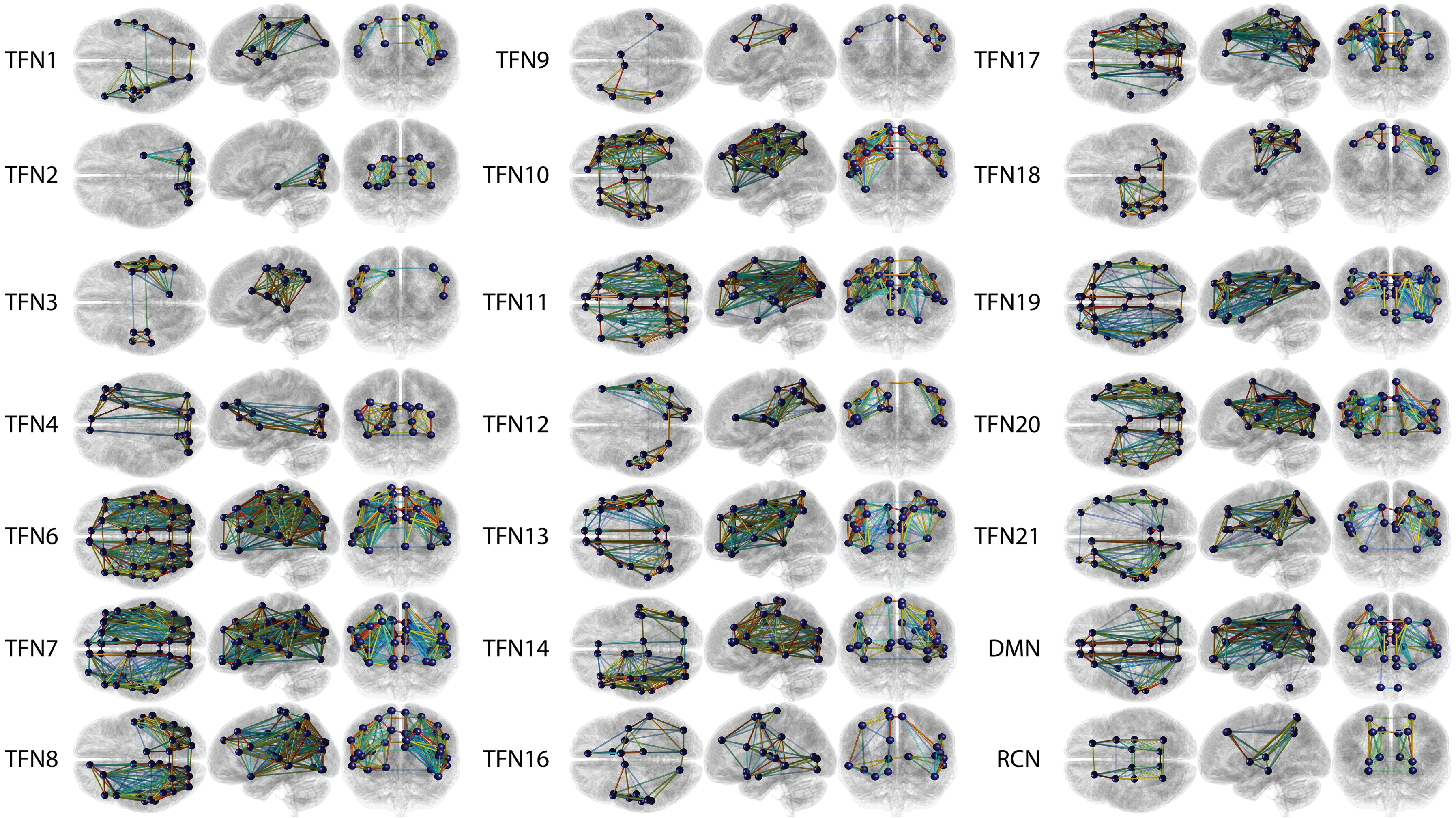

Figure S5.1. Sub-networks derived from TFNs, DNM and RCN for one subject.
